# Enantioselective Organocatalyzed Michael Addition of Isobutyraldehyde to Maleimides in Aqueous Media

**DOI:** 10.3390/molecules27092759

**Published:** 2022-04-25

**Authors:** Jae Ho Shim, Seok Hyun Cheun, Hyeon Soo Kim, Deok-Chan Ha

**Affiliations:** 1 Department of Anatomy, College of Medicine, Korea University, 73, Goryeodae-ro, Seongbuk-gu, Seoul 02481, Korea; anatomykim@korea.ac.kr; 2 Department of Chemistry, College of Science, Korea University, 145 Anam-ro, Seoul 02481, Korea; eamc2@naver.com (S.H.C.); dechha@korea.ac.kr (D.-C.H.)

**Keywords:** organocatalyst, enantioselectivity, Michael addition, aldehydes, succinimides, spironolactone

## Abstract

Thiourea was introduced into (*R,R*)-1,2-diphenylethylenediamine as an organocatalyst to promote the reaction between isobutyraldehydes and maleimides. Enantioselective Michael addition reaction was carried out as an eco-friendly method using water as the solvent. As a result of the reaction between isobutyraldehyde and maleimide, ≥97% yield and 99% enantioselectivity were obtained at a low catalyst loading of 0.01 mol%. The solvent effect can be explained by theoretical calculations that indicate the participation of a transition state, in which the CF_3_ substituent of the catalyst is a hydrogen bond activated by the surrounding water molecules. This discovery enabled the use of low catalyst loading in the organic reactions of chiral substances for pharmaceutical applications. Furthermore, a solvent effect for Michael reaction of the organocatalysts was proposed, and the organic reaction mechanisms were determined through quantum calculations.

## 1. Introduction

Organocatalysis using small organic molecules for the asymmetric transformation of organic compounds is a well-known method for synthesizing organic compounds for various purposes [1,2,3,4,5,6]. In the last two decades, various organocatalysts have emerged as promising solutions to unsolved problems in modern catalysis, providing complementary systems of activation modes; thus, organic catalysts have been classified as one of the main catalyst categories alongside asymmetric, transition metal, and biological catalysts. In general, organocatalysts typically comprise carbon, hydrogen, nitrogen, and sulfur. While the mechanism of organocatalysts is similar to that of metal catalysts, they do not structurally resemble these catalysts that typically comprise metals and ligands. Michael addition is a well-studied organocatalytic transformation that leads to the formation of new C–C bonds in a highly enantioselective manner. In Michael reactions, the addition of α,α-disubstituted aldehydes to maleimides is an attractive modification because the obtained product is considered a valuable synthetic target and a precursor of biologically interesting compounds [7]. Another interesting feature of this transformation is the resulting formation of a quaternary center [8,9,10,11,12]. This reaction is typically promoted by a bifunctional organocatalyst containing a primary amino group and a thiourea motif [13,14,15,16,17,18,19,20].

Asymmetric Michael reactions between maleimides and aldehydes have been studied by several groups. Wang et al. used a primary amine catalyst to promote the Michael addition of aldehydes with *N*-phenylmaleimide [21]. Tao performed a Michael reaction using a small amount of bifunctional thiourea derivative as a catalyst and demonstrated that the use of benzoic acid as an additive resulted in desirable yield and stereoselectivity [22]. Benaglia et al. reported a simple synthesis of a primary amine-containing organocatalyst with a structure similar to that of the Takemoto catalyst and studied Michael addition using the obtained catalyst [23]. Kokotos obtained the (*S*)-configuration product after conducting the Michael addition of various aldehydes and maleimide with *β*-phenylalanine as the catalyst and reported that the (*R*)-product was the result when aspartic acid was used as the catalyst. The use of *β*-phenylalanine and aspartic acid as catalysts resulted in high yields, enantioselectivities, and desirable diastereoselectivities [24]. Chinchilla reported the Michael addition with a monoprotected 1,2-diamine as catalyst and dimethylformamide/H_2_O (2:1) as solvent, which afforded the (*R*)-product. However, the use of chloroform or dichloromethane as the solvent resulted in a switch in selectivity and yielded the (*S*)-product [25]. In our recent study, organocatalysis of various reactions using thiourea catalysts derived from (*R*,*R*)-1,2-diphenylethylenediamine (DPEN) was reported [13,14,15,16,17,18,19,20]. Additionally, the correlation between the reactivity of the organocatalyst and the reaction outcome in water as an eco-friendly reaction solvent was investigated [26,27].

**Figure 1:**
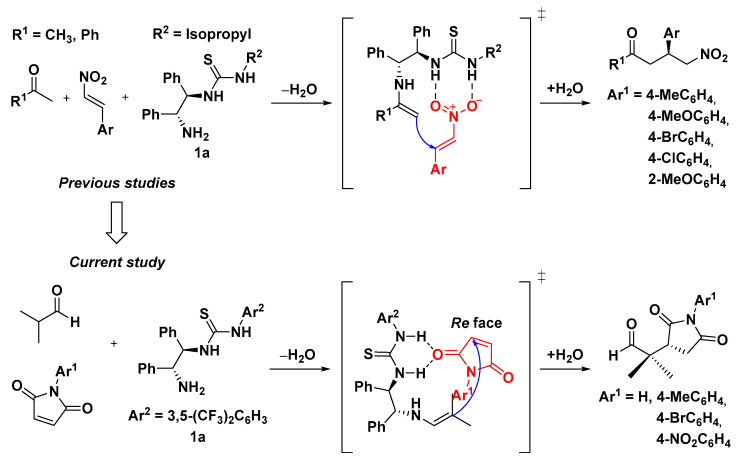
Mechanism of the Michael reaction using a non-covalent organocatalyst in aqueous media.

Similar to those used in previous studies, the basic skeleton of the organocatalyst used in this study comprised DPEN; thiourea was used as an additional skeleton. To synthesize a *γ*-lactone compound, a Michael addition reaction was performed with maleimide and an aldehyde as the electrophile and nucleophile, respectively [26,27,28,29,30,31,32,33,34,35,36]. Here, the correlation between the catalytic reaction and water as solvent was investigated through quantum calculations. Finally, we confirmed the role of water as an eco-friendly solvent in organocatalysis (Figure 1).

## 2. Results and Discussion

### 2.1. Asymmetric Michael Reaction of Maleimide and Aldehydes Using a Thiourea Catalyst

**Figure 2:**
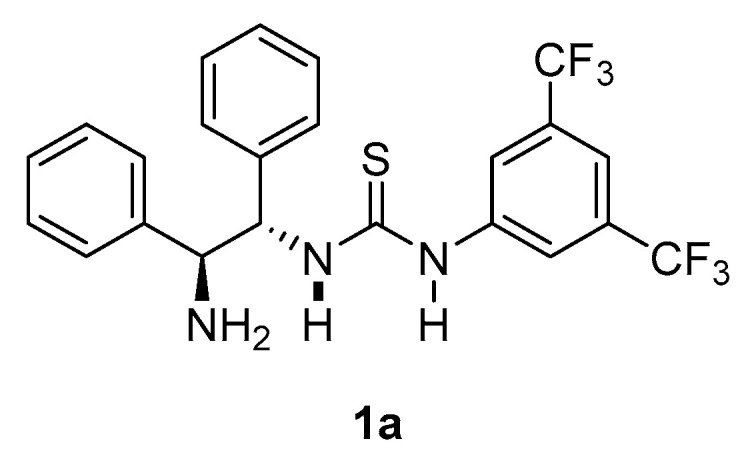
Catalysts based on DPEN and thiourea.

To investigate the effect of the catalyst on the enantioselective Michael reaction of aldehydes and maleimides, reactions between isobutyraldehyde and maleimides were studied. DPEN was used as the basic backbone, and the Michael addition was performed using a catalyst in which thiourea replaced one of the amines. The 3,5-CF_3_ benzyl- (**1a**) substituted thiourea catalysts were both evaluated in the Michael reaction (Figure 2).

To examine the effect of the catalyst, CH_2_Cl_2_ was used as a solvent, and the reaction was performed at room temperature (Table 1). The highest yield and stereoselectivity were obtained using the catalyst substituted with the 3,5-bis(trifluoromethyl) group (**1a**). After confirming that 3,5-bis(trifluoromethyl) (**1a**) exhibited the highest stereoselectivity, an acid additive to further enhance the catalysis was evaluated. In particular, the aldehyde was activated by adding a weak acid additive to promote catalyst function and imine formation. With the addition of benzoic acid, the yield of the reaction increased slightly (entry 4). When the solvent of the reaction was changed to toluene, good yield and stereoselectivity were obtained (entry 6). However, when water was used as the solvent, catalyst **1a** provided the highest reactivity and enantioselectivity with optimal yield and stereoselectivity even in the absence of a weak acid additive (entry 8). In particular, for entry 10 in Table 1, the catalyst loading amount of 0.01 mol% for 12 h resulted in excellent yield and stereoselectivity.

The reaction between isobutyraldehyde and various maleimides was carried out under the optimized conditions determined in Table 1. A reactant substituted with the maleimide *N*-phenyl maleimide was used, electron-withdrawing groups 4-Br, 4-NO_2_, and 4-Me (Table 2, entry 2, 3, 4). Additionally, when the maleimide was not substituted with the phenyl group, the reaction time was shorter than optimal (entry 1). This difference slightly affects the stabilization of maleimide as an electron acceptor when the ketone group of maleimide binds with the thiourea hydrogen of the organocatalyst. So, the thermodynamic energy difference, solvent effect, and mechanism were confirmed through quantum calculations to determine the correlation between the organocatalysts and reactants (Table 2).

### 2.2. Reaction Mechanism Inferred through Expected Transition States

**Figure 3:**
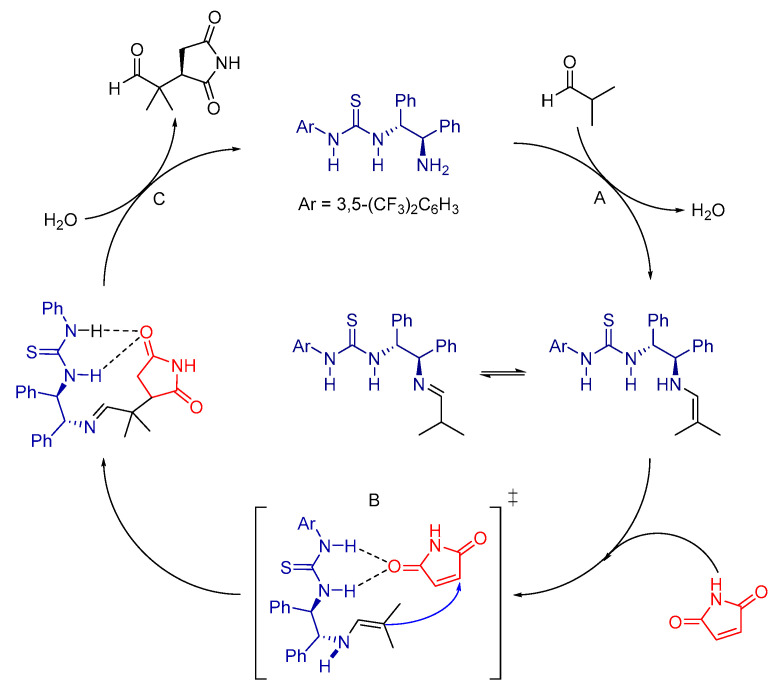
Catalytic cycle of the Michael reaction and proposed transition states.

In step A, the **1a** catalyst reacts with isobutyraldehyde to form an imine, which forms an enamine. Additionally, the **1a** catalyst and maleimide activate the electrophile through hydrogen bonding with the thiourea of the catalyst. As in step B, a transition state including an activated electrophile is formed. Additionally, the reaction is expected to minimize the steric hindrance of maleimide by the re-face attack. Finally, the catalyst and the product are separated through hydrolysis as in step C. Therefore, it was possible to obtain the (*R*)-enantiomer rather than the (*S*)-enantiomer as the main product (Figure 3).

**Figure 4:**
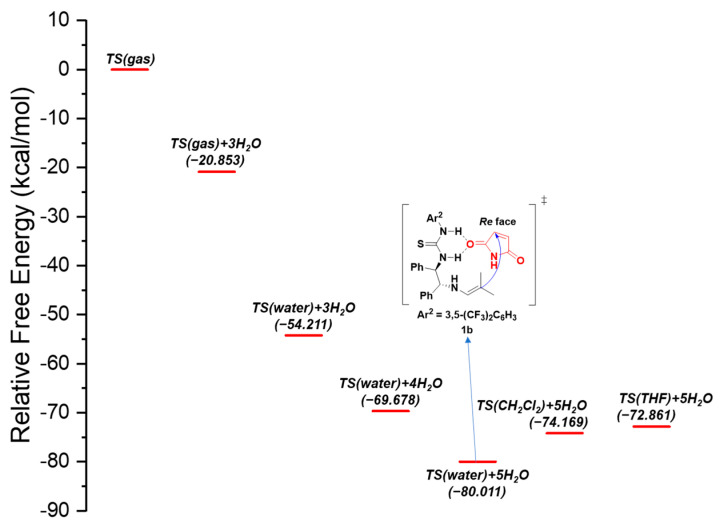
Proposed catalytic transition states based on the B3LYP/6-31G (d, p) method. Calculations were performed using water, toluene, and solvent + nH_2_O conditions. Relative free energy diagram of the (*R*,*R*)-1,2-diphenylethylenediamine (DPEN)–thiourea-catalyzed enantioselective Michael reaction.

To obtain a more accurate prediction of the solvent effect on the catalyst, the relative free energy of the transition states (TS) during the interfacial reaction between the fluorine substituent of trifluorophenyl of catalyst **1b** and water as solvent was calculated in an aqueous binary mixture (H_2_O + solvent, Figure 4) in the following manner. The lowest relative free energy of TS was calculated when water was used as solvent. As shown in Figure 4, the fluorine atom of the 3,5-bis(trifluoromethyl)phenyl group of the catalyst is predicted to interact with the proton of the water solvent through hydrogen bonding. In particular, the relative free energy decreases as the number of hydrogen bonds in water increases. In addition, when water is used as the solvent in the Michael addition reactions, reactivity increases as the stabilization of the relative energy and the hydrophobic effect of the hydration reaction (Figure 4).

**Figure 5:**
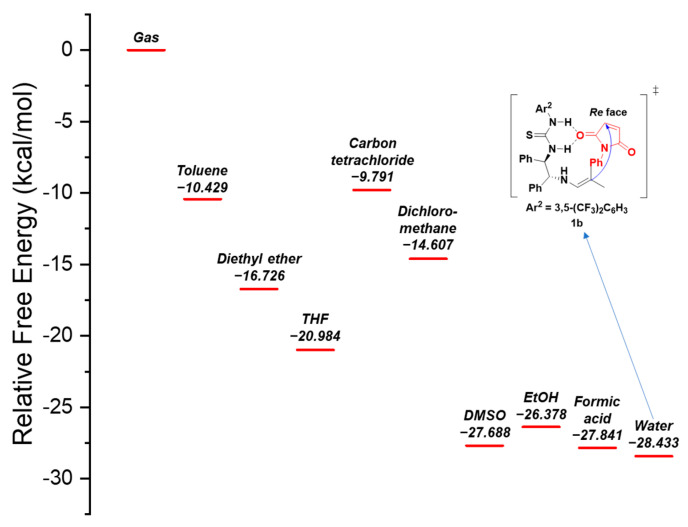
Proposed catalytic transition states based on B3LYP/6-31G (d, p) for the solvent + 5H_2_O condition calculations and the thermal energy diagram of the (*R*,*R*)-1,2-diphenylethylenediamine- (DPEN)–thiourea-catalyzed enantioselective Michael reaction.

Hydrophobic non-polar solvents, such as toluene, afford good yields and stereoselectivities in Michael adduct synthesis [37]. However, the solvent effect on the Michael reaction was confirmed in Figure 4; thus, thermodynamic analysis was performed to determine the factors affecting Michael addition with water. To this end, quantum calculations were performed to predict the relative free energy of the interfacial reaction system in the TS of the catalyst (Figure 5).

A comparison of the actual reaction findings (Table 1) with the quantum calculation results confirmed that the non-polar solvent toluene exhibited the lowest reactivity. In addition, tetrahydrofuran and CH_2_Cl_2_ exhibited similar reactivities in the calculated results. Notably, water showed the best reactivity and stability, with the obtained results exceeding those recorded for polar hydrophilic solvents dimethyl sulfoxide and ethanol and weak acids, such as aqueous formic acid (Figure 5). The optimization structures for DFT calculations mentioned in this article can be found on Supplementary Material page 27–125.

### 2.3. Pharmaceutical Applications Using Gram-Scale Asymmetric Reactions

**Figure 6:**
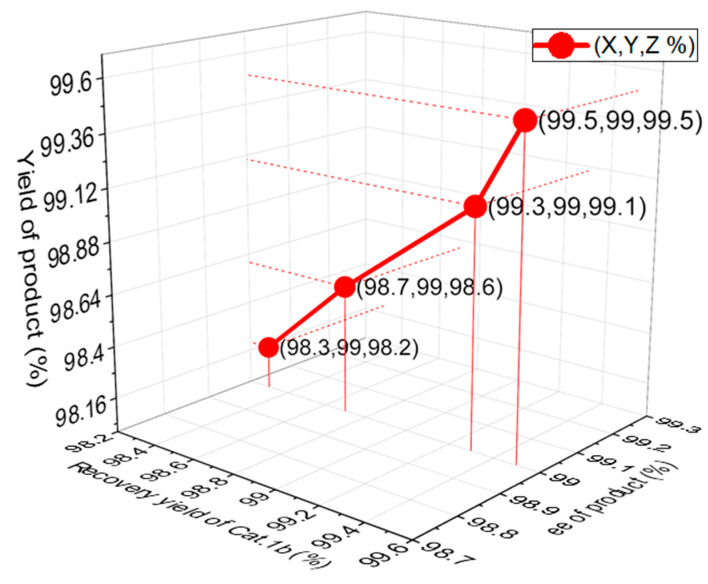
Recycling test for asymmetric Michael addition using chiral (*R*,*R*)-1,2-diphenylethylene-diamine-derived thiourea catalyst (**1b**).

**Figure 7:**
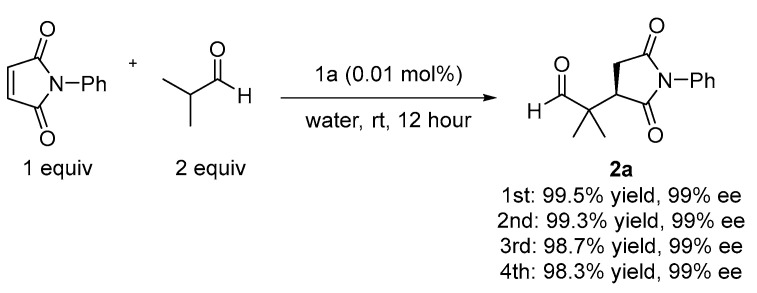
Gram-scale reaction of *N*-phenylmaleimide and isobutyraldehyde and catalyst recycling through catalyst recovery.

Next, catalyst **1b** recycling was evaluated (Figure 6 and Figure 7). The results of four-time tests indicated that the DPEN-derived thiourea catalyst (**1b**) was sufficiently recyclable (99.5–98.3%). In addition, the stereoselectivity of the product (99%) was maintained during the four reuse cycles of the catalyst. To synthesize the spironolactone, a physiologically active material, using the recovered catalyst, a gram-scale reaction was performed to add a cyclohexyl group to an aldehyde, as described below.

**Figure 8:**
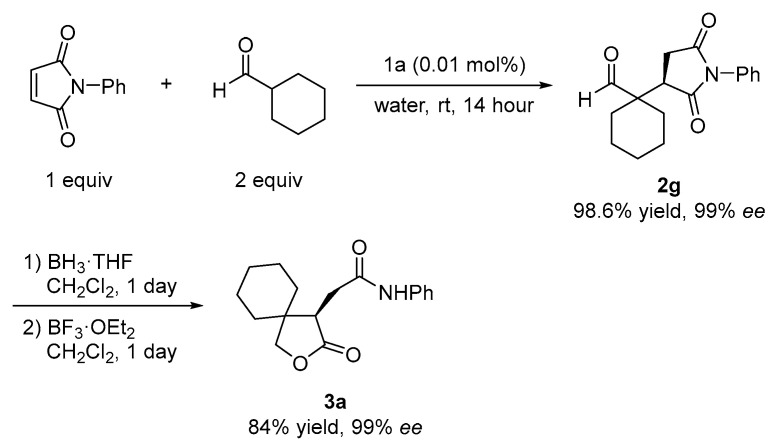
Gram-scale reaction for the application of spironolactone for the Michael reaction of cyclohexanecarboxaldehyde and *N*-phenylmaleimide.

The Michael addition of isobutyraldehyde to *N*-phenylmaleimide (5 g) was studied; notably, when the reaction was performed using cyclohexanecarboxaldehyde instead of isobutyraldehyde, **2g** of the desired product was obtained in 98.6% yield (8.19 g) with 99% enantiomeric excess (*ee*). As summarized in Figure 8, spirolactone **3a** was produced by the reaction of this product with BH_3_·THF and BF_3_·Et_2_O. The spironolactone series are known diuretics with potential medicinal applications (Figure 8) [24].

## 3. Materials and Methods

### 3.1. Instruments and Reagents

Optical rotation was measured using an auto digital polarimeter, and Fourier transform infrared (FT-IR) spectra were recorded using Nicolet 380 FT-IR spectrophotometer (Thermo Electron Corporation). Nuclear magnetic resonance (^1^H NMR and ^13^C NMR) spectra were obtained using Varian Gemini 300 (300, 75 MHz) and Bruker Avance 500 (500, 125 MHz) using tetramethylsilane as internal standard. Low-resolution mass spectrometry profiles were obtained using a JEOL the MStation JMS-700. Chiral high-performance liquid chromatography (HPLC) analysis was performed using a Jasco LC-1500 Series HPLC system. Toluene (CaH_2_), tetrahydrofuran (THF) (Na, benzophenone), and CH_2_Cl_2_ (CaH_2_) reaction solvents were purified before use. The reagents used in this study were obtained from Aldrich, Acros, Alfa, Sigma, Merck, Fluka, TCI, and Lancaster, and purified or dried using a known method when necessary. Merck’s silica gel 60 (230–400 mesh) was used as the stationary phase in column chromatography.

### 3.2. Experimental Method

#### 3.2.1. Synthesis of *N*-Mono-Thiourea Catalyst

**Scheme 1:**
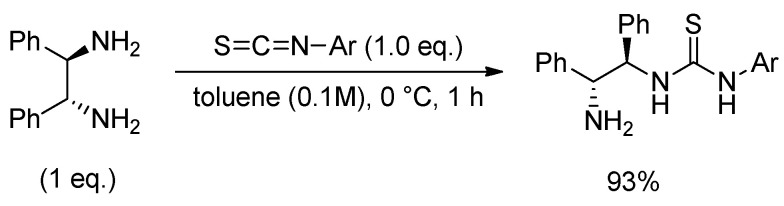
Synthesis of *N*-mono-thiourea catalyst.

DPEN (0.5 g, 0.235 mmol) was dissolved in toluene (2.50 mL). Then, the solution was added with isothiocyanate (0.35 mL, 0.235 mmol) and stirred for 1 h at 0 °C, and the reaction was terminated with distilled water. The mixture was extracted with ethyl acetate (20 mL × 3 times), dehydrated using MgSO_4_, filtered, concentrated under reduced pressure, and purified using column chromatography (SiO_2_, CH_2_Cl_2_: *n*-hexane = 1:2) to isolate the product (Scheme 1).

#### 3.2.2. Asymmetric Michael Reaction of Aldehyde and Maleimide Using a Chiral Thiourea Catalyst

At room temperature, an *N*-mono-thiourea catalyst (0.01 mol%) and *N*-phenylmaleimide (2.88 mmol) were placed in a reaction vessel and then dissolved with water (0.1 mL) in air. Then, aldehyde (2 equiv.) was added to the mixture, which was stirred for 10–13 h. After the reaction was terminated with distilled water, the mixture was extracted with ethyl acetate (0.3 mL × 3 times), dehydrated using MgSO_4_, filtered, concentrated under reduced pressure, and purified using column chromatography (SiO_2_, CH_2_Cl_2_: *n*-hexane = 1:3) to isolate the product.

#### 3.2.3. Gram-Scale Asymmetric Michael Reaction of Aldehyde and Maleimide Using a Chiral Thiourea Catalyst

At room temperature, an *N*-mono-thiourea catalyst (0.01 mol%) and *N*-phenylmaleimide (288.7 mmol) were placed in a reaction vessel and then dissolved with water (10 mL) in air condition. Then, aldehyde (2 equiv.) was added to the mixture and stirred for 10–13 h. After the reaction was terminated with distilled water, the mixture was extracted with ethyl acetate (30 mL × 3 times), dehydrated using MgSO_4_, filtered, concentrated under reduced pressure, and purified using column chromatography (SiO_2_, CH_2_Cl_2_: *n*-hexane = 1:3) to isolate the product. Finally, the column chromatography conditions were changed (SiO_2_, CH_2_Cl_2_: *n*-hexane = 1:3) to isolate the catalyst.

#### 3.2.4. General Procedure of the Racemic Michael Addition

*N*-Phenylmaleimide (0.3 mmol), aldehyde (10 equiv.), and 20 mol% of *DL*-proline were added to toluene (1 mL), and the reaction mixture was stirred at ambient temperature. The reaction conversion was checked by thin-layer chromatography. Upon the completion of the reaction after approximately 12 h, ethyl acetate (0.2 mL) was added to the reaction product. This solution was washed twice with water (2 × 1.0 mL), dried over MgSO_4_ (anhydrous), and concentrated to yield the desired product. The product was purified by chromatography on a silica gel column eluted with a mixed solvent (CH_2_Cl_2_: *n*-hexane = 1:3).

### 3.3. Results of Density Functional Theory Calculations and Discussion

Density functional theory (DFT) calculations were performed using Gaussian 16 and Gauss-View 6.0 programs. DFT calculations were performed to show the mechanisms of substrates and catalysts. The optimized geometry was described using the DFT method with the Becke three-parameter Lee–Yang–Parr (B3LYP) level [37]. Single-point calculations for the optimized geometries were then performed by using the 6-31G (d, p) basic set. After the shapes of reactants and transition states (TS) were fully optimized, the thermodynamic functions and parameters (Gibbs free energy G) of reactants were obtained through vibrational frequency calculation. Additionally, at the same level of theory, the minimum or transition state energy was obtained. Enthalpy correction and entropy with temperature were calculated at 298 K and 1 atm pressure.

## 4. Conclusions

Herein, we report the experimental and theoretical analyses of DPEN-based catalysts for use as chiral bifunctional organocatalysts in asymmetric Michael addition to unsaturated maleimides under neutral conditions. The reaction delivered an enantiomeric excess range of 94–99%. Michael addition using the catalyst substituted with the 3,5-bis(trifluoromethyl) group (Figure 2, **1b**) exhibited relatively high enantioselectivity and diastereoselectivity. Owing to the low catalyst loading of 0.01 mol% and the straightforward synthetic methodology, these catalysts offer certain economic prospects. Notably, the reaction proceeds without metals and additives, and can be performed in air using water as the solvent, making the method environmentally friendly. In addition to presenting a pharmaceutical application using gram-scale asymmetric reactions, this study sought to gain insights into organocatalytic transformations with water as the solvent, making the reaction eco-friendly. The Michael addition reaction provided high yields and good stereoselectivity owing to the hydrogen bonding effect of the fluorine atoms with other hydrophobic groups in the catalyst. This catalytic methodology can be applied to various pharmaceutical syntheses in the future. The development of drugs bearing chiral compounds using these synthetic approaches is underway.

## Figures and Tables

**Table 1:**
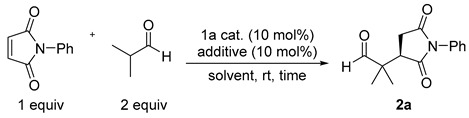
Effect of additive and solvent.

Entry	Time (h)	Additive	Solvent	Yield (%) ^a^	*ee* (%) ^b^
1	24	Trifluoro-acetic acid	CH_2_Cl_2_	78	98
2	24	Acetic acid	CH_2_Cl_2_	83	94
3	24	Salicylic acid	CH_2_Cl_2_	82	94
4	24	Benzoic acid	Toluene	78	99
5	24	Benzoic acid	THF	98	99
6 ^c^	0.67	-	Water	99	99
7 ^d^	6	-	Water	99	99
8 ^e^	12	-	Water	97	99

^a^ Isolated yield. ^b^ Enantiomeric excess (*ee*) values were determined by chiral-phase high-performance liquid chromatography (HPLC) using an OD-H column. Reactions were run with catalyst loading of ^c^ 1, ^d^ 0.1, and ^e^ 0.01 mol%.

**Table 2:**
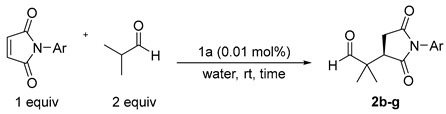
Effect of various types of maleimide on the reaction.

Entry	Ar	Time (h)	Yield (%) ^a^	*ee* (%) ^b^
1	H	10	98	99
2	4-MeC_6_H_4_	12	99	99
3	4-BrC_6_H_4_	12	98	99
4	4-NO_2_C_6_H_4_	12	97	99

^a^ Isolated yield. ^b^ *ee* values were determined by chiral-phase HPLC using OD-H, AD-H, and AS-H columns.

## Data Availability

Not applicable.

## Supplementary Materials

The following are available online at https://www.mdpi.com/article/10.3390/molecules27092759/s1, Compound Characterization Data, Copy of NMR and MASS Spectra, Copy of HPLC Chromatograms, and DFT Calculations for all Calculated Structures of the compounds mentioned in the text. The text is inserted as follows. References [21,22,24,38,39,40,41] are cited in the Supplementary Materials.
