# Supplementary material for: Enantioselective Organocatalyzed Michael Addition of Isobutyraldehyde to Maleimides in Aqueous Media
Source: Molecules. 2022 Apr 25;27(9):2759. doi: 10.3390/molecules27092759 (PMC9101813; doi:10.3390/molecules27092759)
Supplement: Supplementary file 1 [file molecules-27-02759-s001.zip › molecules-1690352-supplementary.pdf]

Enantioselective Organocatalyzed Michael Addition of Isobutyraldehydes to Maleimides in Aqueous Media

Jae Ho Shim [1,*], *Seok Hyun Cheun* [2], *Hyeon Soo Kim* [1], *and Deok-Chan Ha* [2]

1. *Department of Anatomy, College of Medicine, Korea University, 73, Goryeodae-ro, Seoul, 0284, Republic of Korea, E-mail: shimjh3000@korea.ac.kr, anatomykim@korea.ac.kr*
2. *Department of Chemistry, College of Science, Korea University, 145 Anam-ro , Seoul 02841, Korea E-mail: dechha@korea.ac.kr, eamc2@naver.com*

Supporting Information

Table of Contents

1. Compound Characterization Data………………………………………… S-2
2. Copy of HPLC, NMR and MASS Spectra……………………………….... S-7
3. DFT Calculations for all Calculated Structures…………………………. S-27
4. Reference……………………………………………………………….…….S-126

1. Compound Characterization Data

1-[(1*R*,2*R*)-2-Amino-1,2-diphenylethyl]-3-[3,5-bis(trifluoromethyl)phenyl]thiourea(1a)

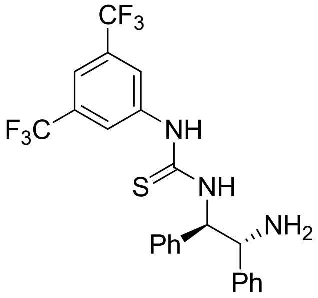

$[\alpha]_D^{25}$ +13.5 (*c* 1.0, CH_3Cl); ^{1}H NMR (500 MHz, DMSO-d_6) δ 8.25 (s, 2H), 7.78 (s, 1H),7.32~7.15 (m, 13H), 5.99 (d, *J* = 3 Hz, 1H), 4.77 (d, *J* = 3 Hz, 1H) ppm; ^{13}C NMR (125 MHz, DMSO-d_6) δ 180.5, 143.2, 142.4, 130.8, 130.5, 128.5, 128.2, 127.6, 127.5, 127.3, 124.7, 122.6, 121.3, 116.0, 63.6, 59.9 ppm; IR (KBr) 3305, 3032, 2963, 1652,1601, 1557, 1383, 1277, 1262, 803, 700 cm^{-1}; HRMS (FAB+) for $C_{23}H_{19}F_6N_3S$ $[M+H]^+$ Calcd: 484.1282, Found: 484.1254;

(*R*)-2-(2,5-Dioxo-1-phenylpyrrolidin-3-yl)-2-methylpropanal (2a)[1,2]

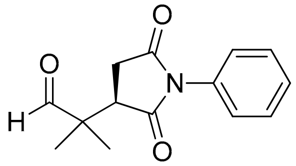

$[\alpha]_D^{25}$ +6.2 (*c* 0.2, CH_2Cl_2); ^{1}H NMR (300 MHz, $CDCl_3$) δ 9.51 (s, 1H), 7.26~7.50 (m, 5H), 3.14 (dd, *J* = 6.0, 12 Hz, 1H), 2.96 (dd, *J* = 9.0, 18 Hz, 1H), 2.60 (dd, *J* = 6.0, 12 Hz, 1H), 1.32 (s, 3H), 1.27 (s, 3H) ppm; ^{13}C NMR (100 MHz, $CDCl_3$) δ 203.0, 177.1, 175.0, 132.0, 129.4, 128.9, 126.7, 48.8, 45.2, 32.1, 20.6, 19.8 ppm; LRMS (EI$^+$) Calcd. for $[C_{14}H_{15}NO_3]^+$: 245, found: 245; HPLC [Chiralcel OD-H, hexane/2-propanol = 75/25, flow rate = 0.7 mL/min, λ = 210nm, retention times: (major) 38.8 min, (minor) 32.3 min].

(*R*)-2-(2,5-Dioxopyrrolidin-3-yl)-2-methylpropanal (2b)[7]

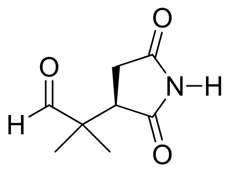

$[\alpha]_D^{21}$ –9.00 (*c* 0.2, CH_2Cl_2); ^1H NMR (300 MHz, $CDCl_3$) δ 9.49 (s, 1H), 8.57 (br. s, 1H), 3.09 (dd, *J* = 6.0, 9.0 Hz, 1H), 2.85 (dd, *J* = 12, 18 Hz, 1H), 2.51 (dd, *J* = 6.0, 18 Hz, 1H), 1.26 (s, 3H), 1.24 (s, 3H) ppm; ^{13}C NMR (100 MHz, DMSO) δ 185.4, 183.3, 182.8, 53.3, 48.8, 38.8, 29.5, 28.4 ppm; LRMS (EI^+) Calcd. for $[C_8H_{11}NO_3]^+$: 169, found: 169; HPLC [Chiralcel AD-H, hexane/2-propanol = 85/15, flow rate = 0.7 mL/min, λ = 210nm, retention times: (major) 25.3 min, (minor) 33.7 min].

(*R*)-2-(2,5-Dioxo-1-p-tolylpyrrolidin-3-yl)-2-methylpropanal (2c)[7]

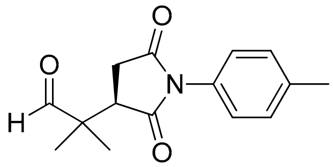

$[\alpha]_D^{21}$ –6.5 (*c* 0.2, CH_2Cl_2); ^1H NMR (300 MHz, $CDCl_3$) δ 9.52 (s, 1H), 7.26 (t, 3H), 7.15 (d , *J* = 9.0 Hz, 2H), 3.14 (dd, *J* = 6.0, 9.0 Hz, 1H), 2.96 (dd, *J* = 9.0, 18 Hz, 1H), 2.56~2.64 (dd, *J* = 6.0, 18 Hz, 1H), 2.37 (s, 3H), 1.31 (s, 3H), 1.28 (s, 3H) ppm; ^{13}C NMR (100 MHz, $CDCl_3$) δ 203.0, 177.2, 175.2, 139.0, 130.1, 129.3, 126.5, 48.7, 45.2, 32.0, 21.4, 20.5, 19.8 ppm; LRMS (EI^+) Calcd. for $[C_{15}H_{17}NO_3]^+$: 259, found: 259; HPLC [Chiralcel OD-H, hexane/2-propanol = 75/25, flow rate = 0.6 mL/min, λ = 210nm, retention times: (major) 37.7 min, (minor) 31.3 min].

(*R*)-2-[1-(4-Bromophenyl)-2,5-dioxopyrrolidin-3-yl]-2-methylpropanal (2d)[3,4]

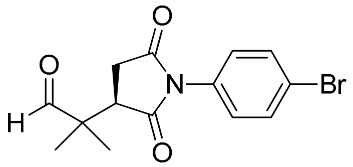

$[\alpha]_D^{20}$ +5.7 (*c* 0.2, CH_2Cl_2); ^{1}H NMR (300 MHz, $CDCl_3$) δ 9.48 (s, 1H), 7.60 (d, *J* = 9.0 Hz, 2H), 7.19 (d, *J* = 6.0 Hz, 2H), 3.11 (dd, *J* = 6.0, 9.0 Hz, 1H), 2.97 (dd, *J* = 9.0, 18 Hz, 1H), 2.60 (dd, *J* = 6.0, 18 Hz, 1H), 1.36 (s, 3H), 1.28 (s, 3H) ppm; ^{13}C NMR (100 MHz, $CDCl_3$) δ 203.0, 176.8, 174.6, 132.6, 131.0, 128.3, 122.8, 48.9, 45.1, 32.2, 20.7, 20.1 ppm; LRMS (EI^+) Calcd. for $[C_{14}H_{14}BrNO_3]^+$: 323, found: 323; HPLC [Chiralcel OD-H, hexane/2-propanol = 75/25, flow rate = 0.6 mL/min, λ = 210nm, retention times: (major) 58.6 min, (minor) 31.5 min].

(*R*)-2-[1-(4-Nitrophenyl)-2,5-dioxopyrrolidin-3-yl]-2-methylpropanal (2e)[1]

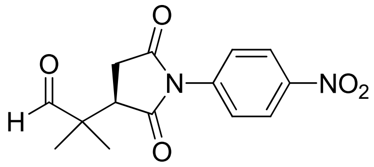

$[\alpha]_D^{20}$ +2.7 (*c* 0.1, CH_2Cl_2); ^{1}H NMR (300 MHz, $CDCl_3$) δ 9.47 (s, 1H), 8.33 (d, *J* = 9.0 Hz, 2H), 7.58 (d, *J* = 9.0 Hz, 2H), 3.13 (dd, *J* = 6.0, 12 Hz, 1H), 3.02 (dd, *J* = 12, 18 Hz, 1H), 2.68 (dd, *J* = 6.0, 18 Hz, 1H), 1.42 (s, 3H), 1.31 (s, 3H) ppm; ^{13}C NMR (100 MHz, $CDCl_3$) δ 203.0, 176.5, 174.1, 147.2, 137.6, 127.3, 124.6, 49.2, 45.2, 32.4, 21.0, 20.5 ppm; LRMS (EI^+) Calcd. for $[C_{14}H_{14}N2O_5]^+$: 290, found: 290; HPLC [Chiralcel OD-H, hexane/2-propanol = 80/20, flow rate = 1.0 mL/min, λ = 210nm, retention times: (major) 72.6 min, (minor) 44.4 min].

(*R*)-1-(2,5-Dioxo-1-phenylpyrrolidin-3-yl)cyclohexanecarbaldehyde (2f)[1,5]

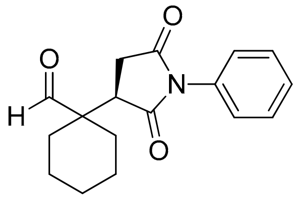

$[\alpha]_D^{20}$ +5.2 (*c* 0.1, CH_2Cl_2); ^{1}H NMR (300 MHz, $CDCl_3$) δ 9.54 (s, 1H), 7.26~7.50 (m, 5H), 3.25 (dd, *J* = 6.0, 9.0 Hz, 1H), 2.88 (dd, *J* = 9.0, 18 Hz, 1H), 2.67 (dd, *J* = 6.0, 18 Hz, 1H), 1.82~2.04 (m, 3H), 1.53~1.64 (m, 7H) ppm; ^{13}C NMR (100 MHz, $CDCl_3$) δ 204.9, 177.4, 175.2, 132.3, 129.5, 129.0, 127.0, 52.6, 43.0, 31.9, 29.0, 28.5, 25.5, 21.8, 21.6 ppm; LRMS (FAB^+) Calcd. for $[C_{17}H_{19}NO_3]^+$: 285, found: 285; HPLC [Chiralcel OD-H, hexane/2-propanol = 75/25, flow rate = 1.0 mL/min, λ = 210nm, retention times: (major) 53.2 min, (minor) 42.4 min].

(*R*)-2-(3-Oxo-2-oxaspiro[4.5]decan-4-yl)-*N*-phenylacetamide (3a)[6]

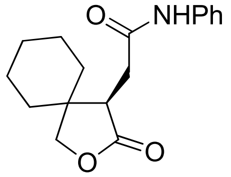

$[\alpha]_D^{20}$ –4.2 (*c* 0.1, CH_2Cl_2); ^{1}H NMR (300 MHz, $CDCl_3$) δ 8.78 (s, 1H), 7.55 (d, *J* = 6.0, Hz, 2H), 7.32 (t, *J* = 6.0 Hz, 2H), 7.10 (t, *J* = 9.0 Hz, 1H), 3.85 (m, 1H), 3.54 (m, 1H), 3.06 (dd, *J* = 3.0, 9.0 Hz, 1H), 2.59 (dd, *J* = 9.0, 15 Hz, 1H), 2.29 (dd, *J* = 3.0, 15 Hz, 1H), 1.60 (m, 4H), 1.39 (m, 4H), 1.25 (m, 2H) ppm; ^{13}C NMR (100 MHz, $CDCl_3$) δ 180.2, 170.0, 138.9, 129.5, 124.7, 120.5, 70.3, 47.3, 46.1, 33.2, 32.0, 28.9, 25.8, 23.1, 19.9 ppm; LRMS (FAB^+) Calcd. for $[C_{17}H_{21}NO_3]^+$: 287, found: 287; HPLC [Chiralcel OD-H, hexane/2-propanol = 90/10, flow rate = 1.0 mL/min, λ = 254nm, retention times: (major) 9.9 min, (minor) 14.9 min].

2. Copy of HPLC, NMR and MASS Spectra

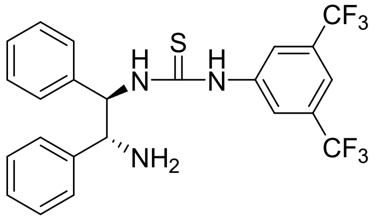

1a

^{1}H NMR

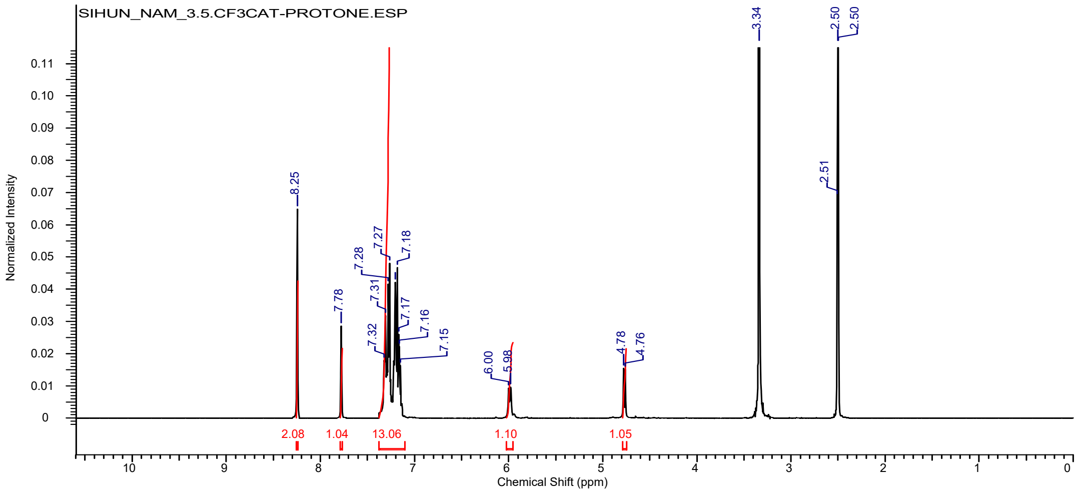

^{13}C NMR

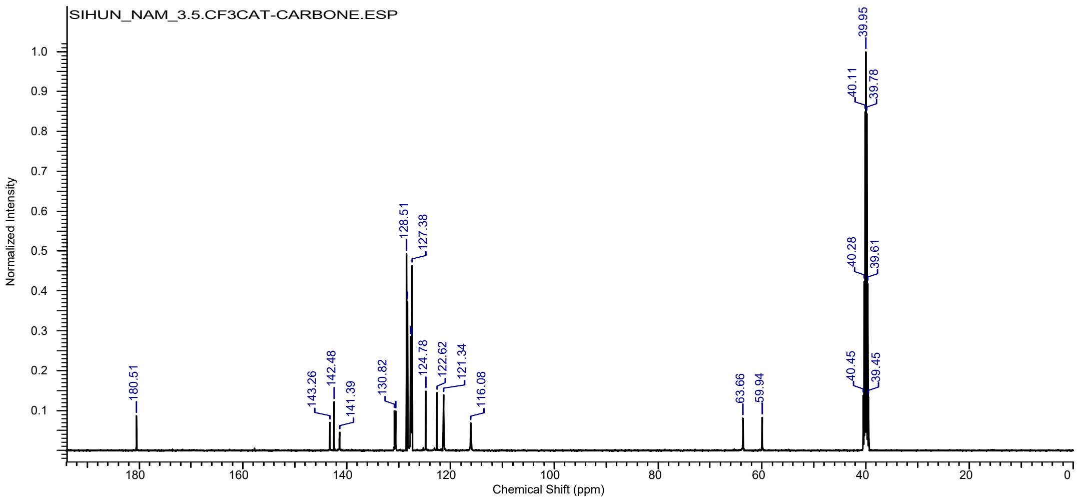

Table 1.

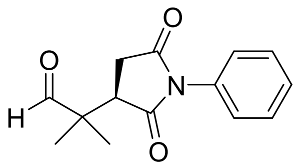

2a

Racemic

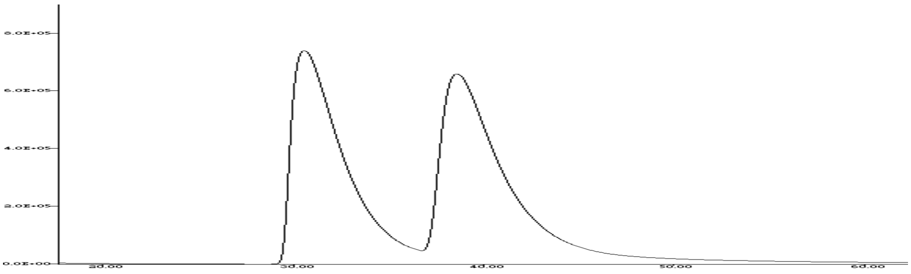

#	Name	RT	Area[uV.Sec]	Quantity
1		30.533	128147734.660	0.000
2		38.567	130363968.160	0.000

Total Area of Peak = 258511702.830 [uV.Sec]

Table 1, entry 1

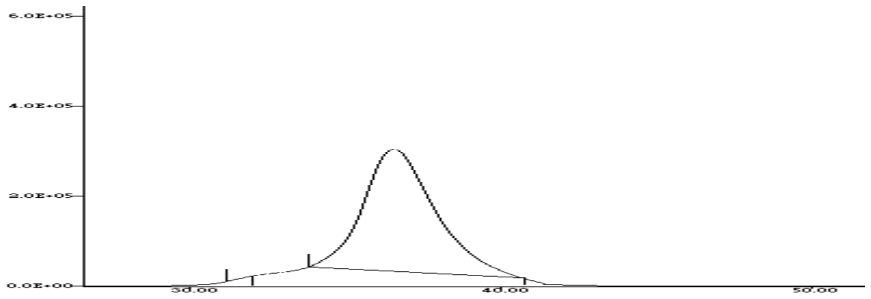

#	Name	RT	Area[uV.Sec]	Quantity
1		32.017	2618451.000	0.000
2		36.525	46704778.000	0.000

Total Area of Peak = 49323229.000 [uV.Sec]

Table 1, entry 2

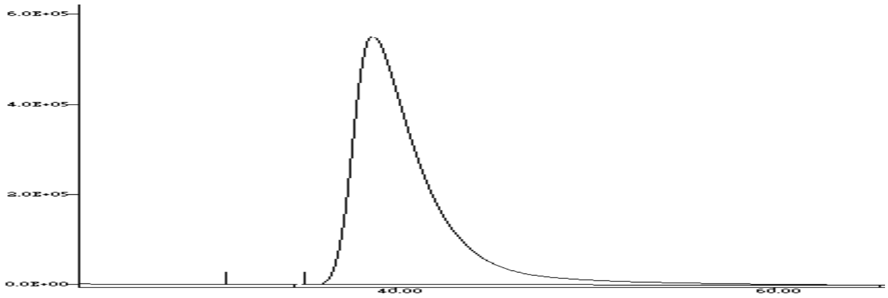

#	Name	RT	Area[uV.Sec]	Quantity
1		32.308	116686.528	0.000
2		38.767	139045145.770	0.000

Total Area of Peak = 139161832.300 [uV.Sec]

Table 1, entry 3

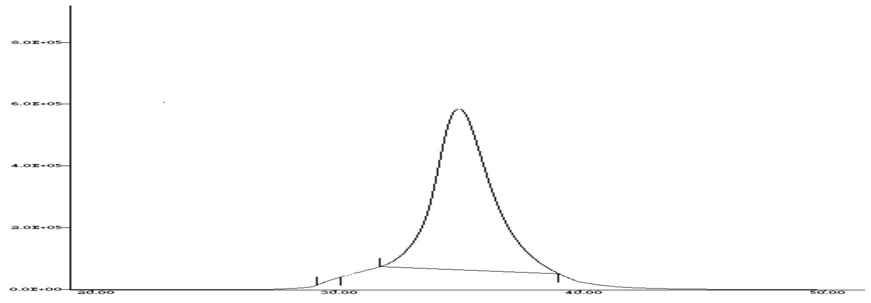

#	Name	RT	Area[uV.Sec]	Quantity
1		30.208	4499914.000	0.000
2		34.992	93686806.678	0.000

Total Area of Peak = 98186720.678 [uV.Sec]

Table 1, entry 4

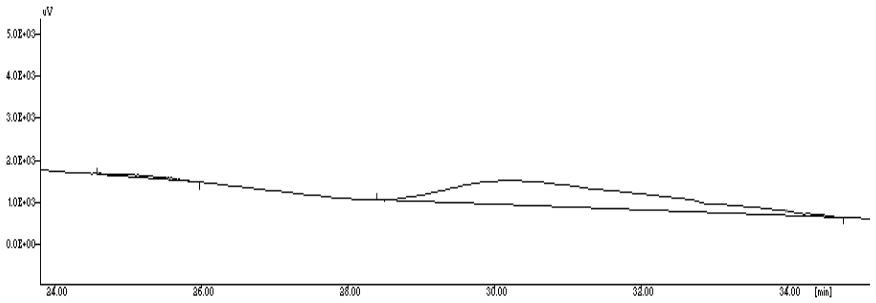

#	Name	RT	Area[uV.Sec]	Quantity
1		24.608	2552.000	0.000
2		30.225	113965.381	0.000

Table 1, entry 5

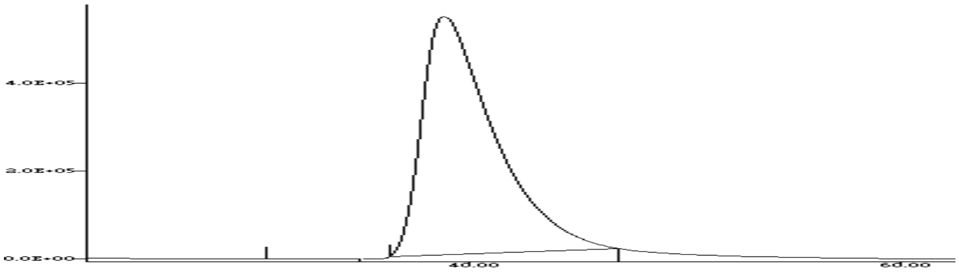

#	Name	RT	Area[uV.Sec]	Quantity
1		32.308	151709.253	0.000
2		38.767127118381.700		0.000

Total Area of Peak = 127270090.950 [uV.Sec]

Table 1, entry 6

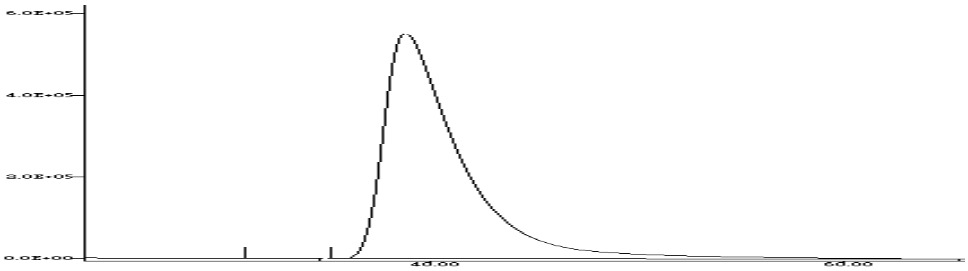

#	Name	RT	Area[uV.Sec]	Quantity
1		32.308	116686.528	0.000
2		38.767139045145.770		0.000

Total Area of Peak = 139161832.300 [uV.Sec]

Table 1, entry 7

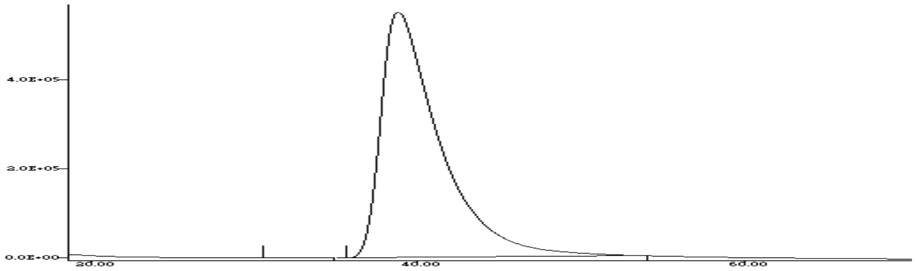

#	Name	RT	Area[uV.Sec]	Quantity
1		32.308	151709.253	0.000
2		38.767134498855.210		0.000

Total Area of Peak = 134650564.460 [uV.Sec]

Table 1, entry 8

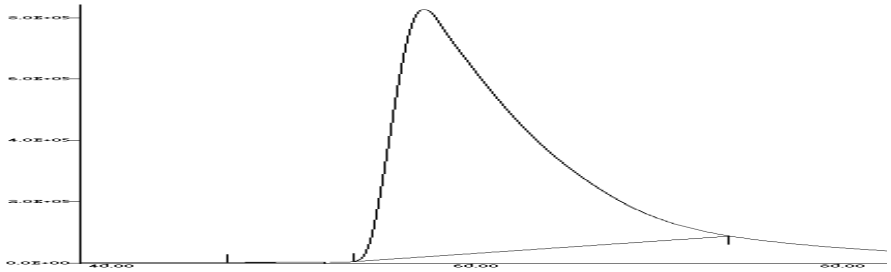

#	Name	RT	Area[uV.Sec]	Quantity
1		49.375	191950.470	0.000
2		57.308384251931.500		0.000

Total Area of Peak = 384443881.970 [uV.Sec]

^{1}H NMR

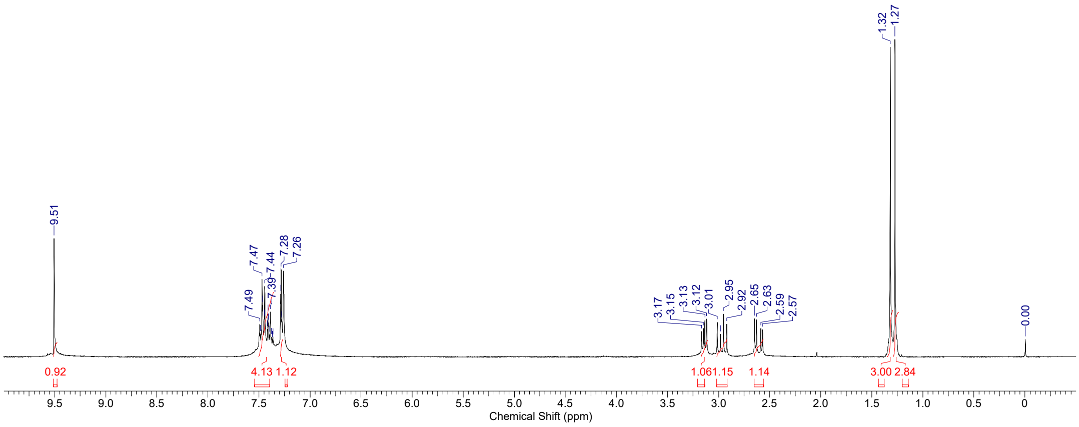

^{13}C NMR

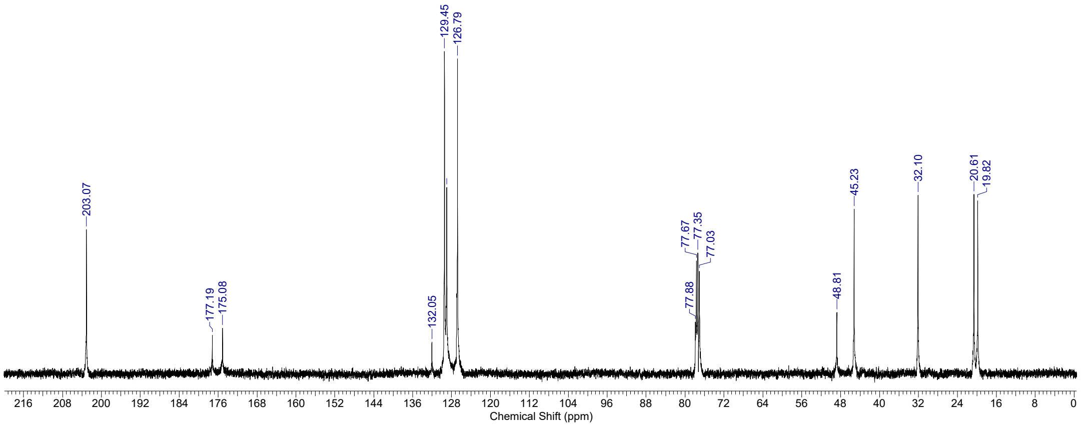

Mass

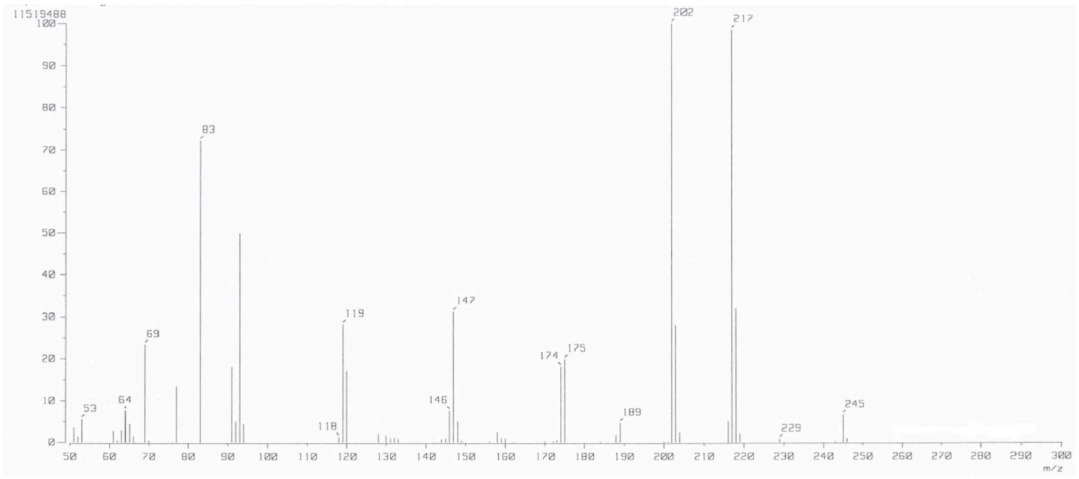

Table 2, entry 1

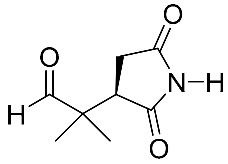

2b

Racemic

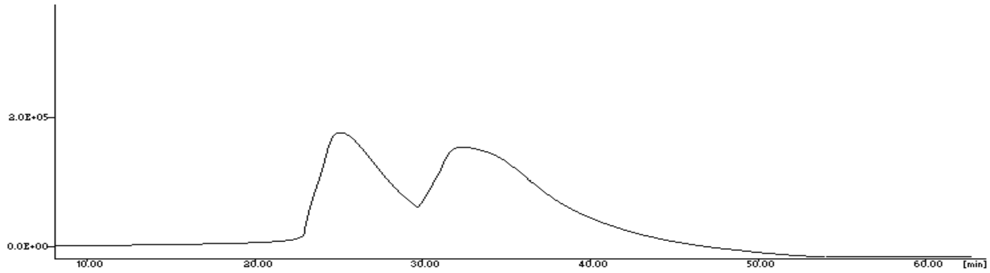

#	Name	RT	Area[uV.Sec]	Quantity
1		25.008	36453687.573	0.000
2		32.308	34838408.000	0.000

Total Area of Peak = 71292095.573 [uV.Sec]

Asymmetric

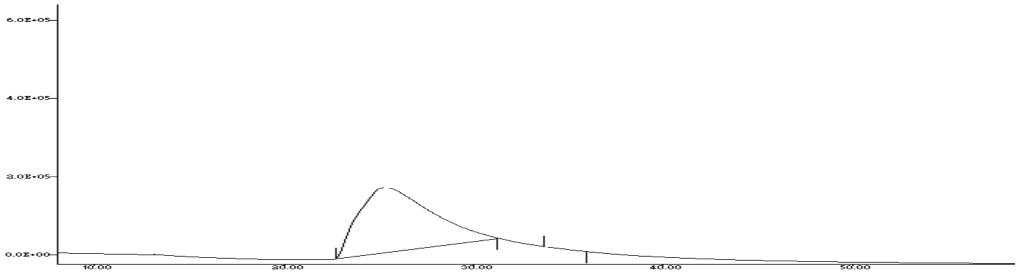

#	Name	RT	Area[uV.Sec]	Quantity
1		25.300	43443193.250	0.000
2		33.742	1792571.000	0.000

Total Area of Peak = 45235764.250 [uV.Sec]

^{1}H NMR

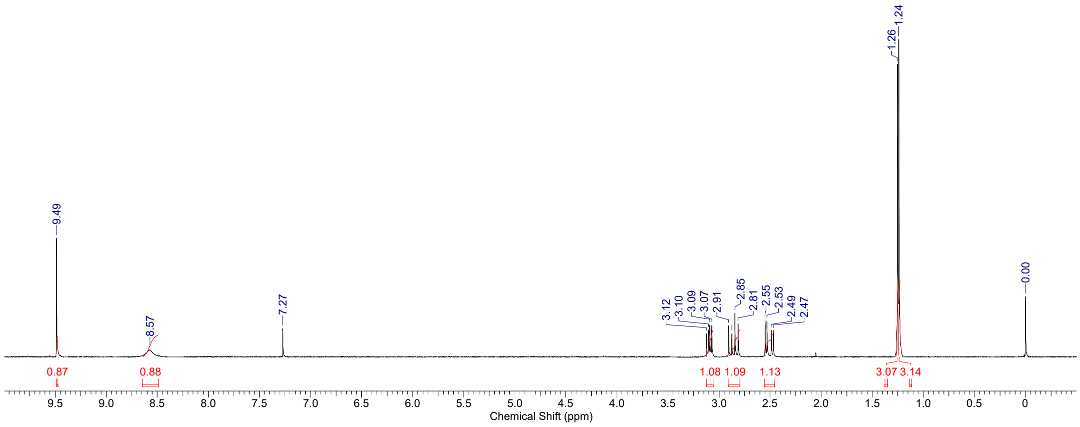

^{13}C NMR

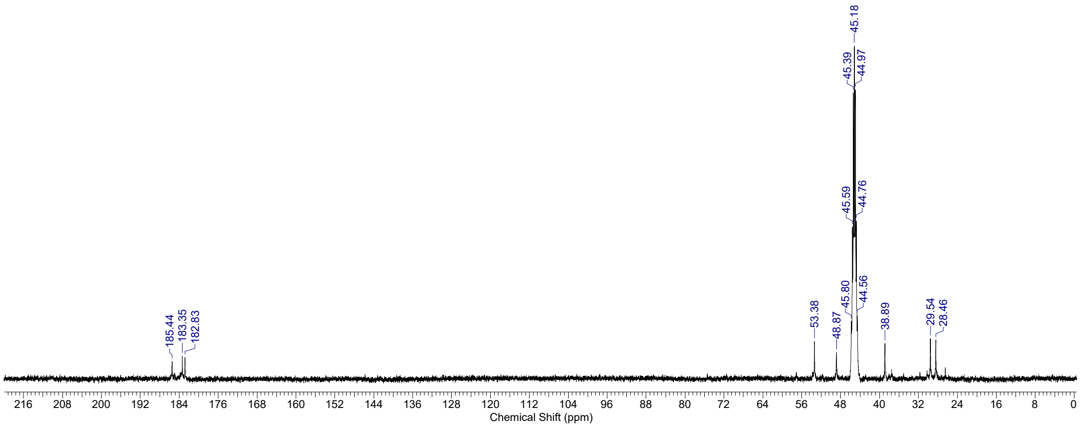

Mass

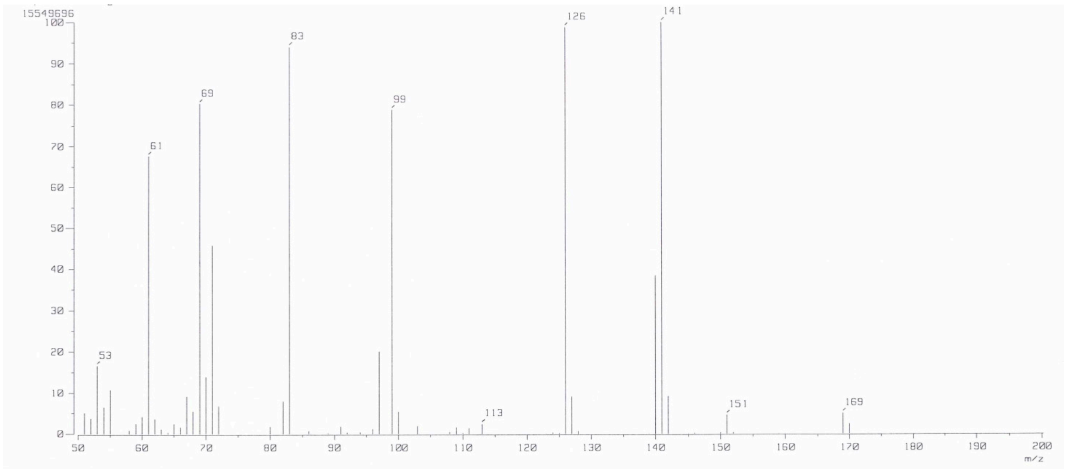

Table 2, entry 2

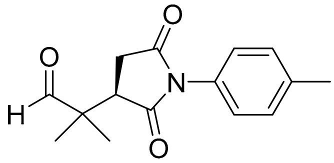

2c

Racemic

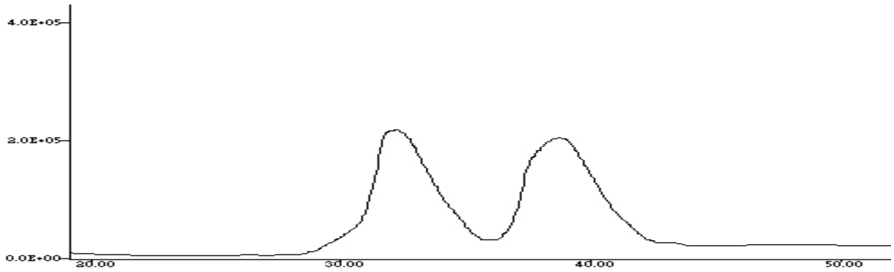

#	Name	RT	Area[uV.Sec]	Quantity
1		31.358	48994375.503	0.000
2		38.958	52721832.569	0.000

Total Area of Peak = 101716208.070 [uV.Sec]

Asymmetric

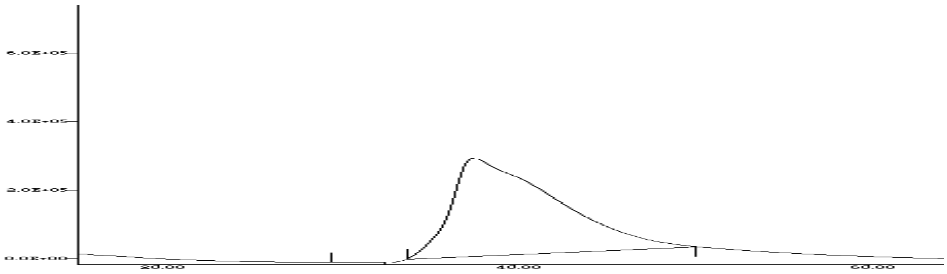

#	Name	RT	Area[uV.Sec]	Quantity
1		31.292	37762.205	0.000
2		37.742	114516169.000	0.000

Total Area of Peak = 114553931.210 [uV.Sec]

^{1}H NMR

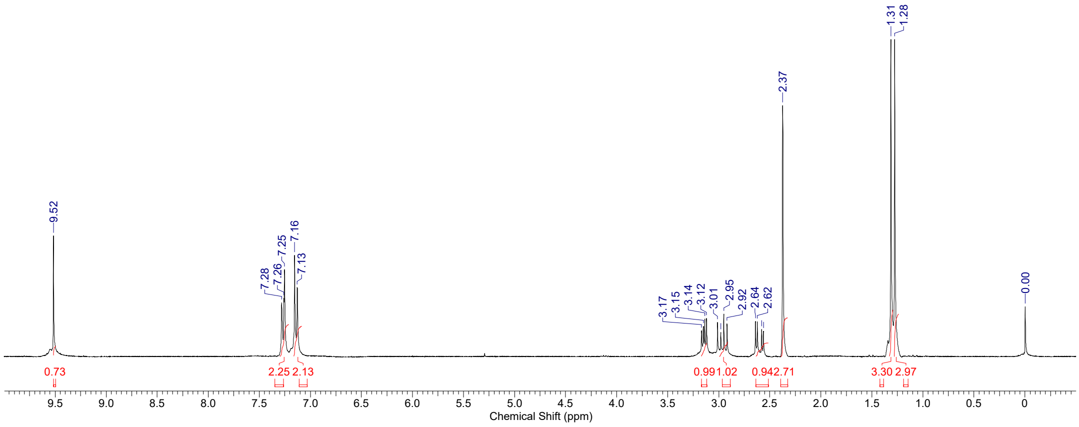

^{13}C NMR

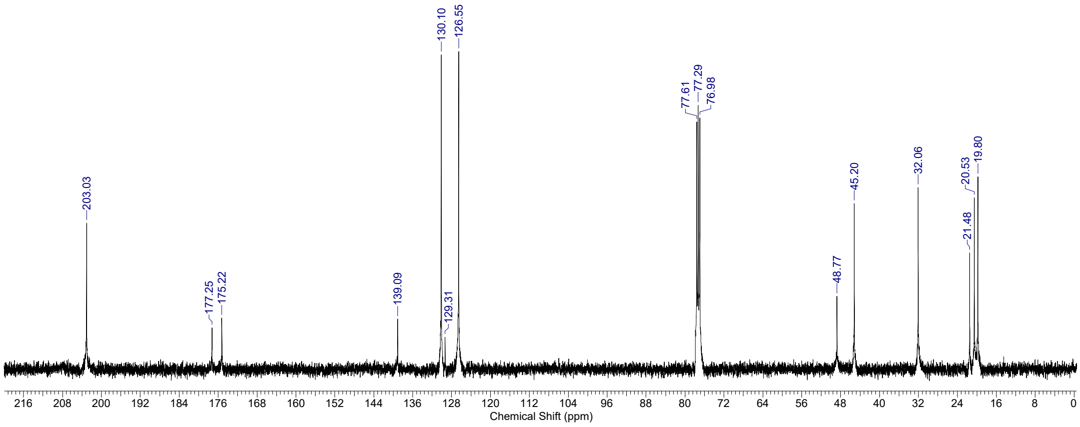

Mass

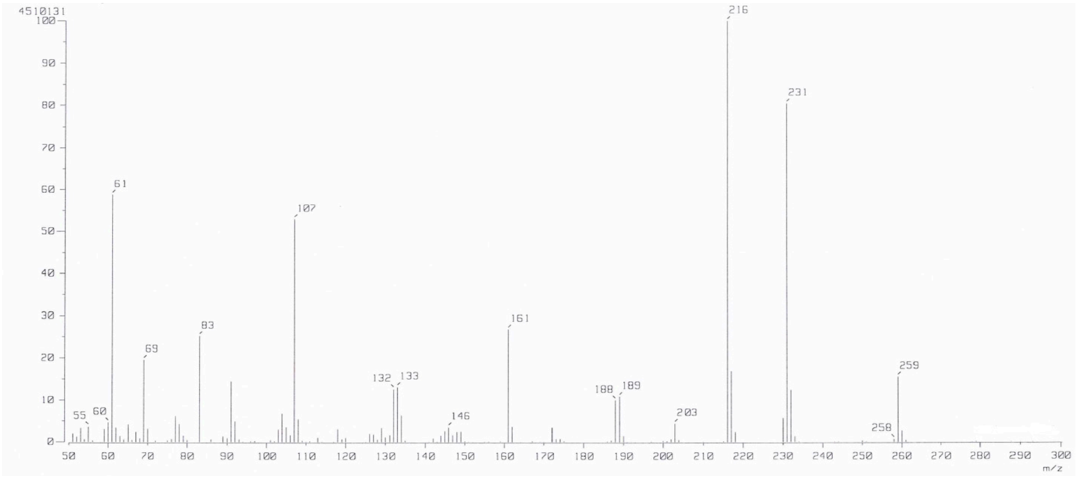

Table 2, entry 3

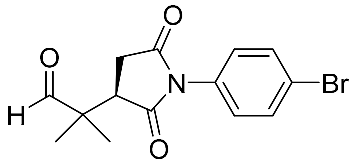

2d

Racemic

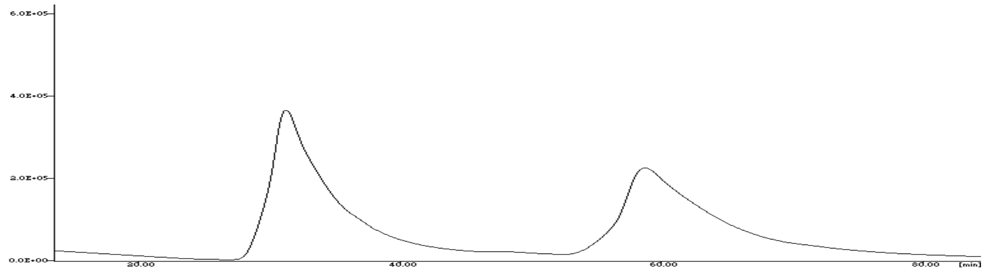

#	Name	RT	Area[uV.Sec]	Quantity
1		31.242	86766751.250	0.000
2		58.717	93286295.875	0.000

Total Area of Peak = 180053047.130 [uV.Sec]

Asymmetic

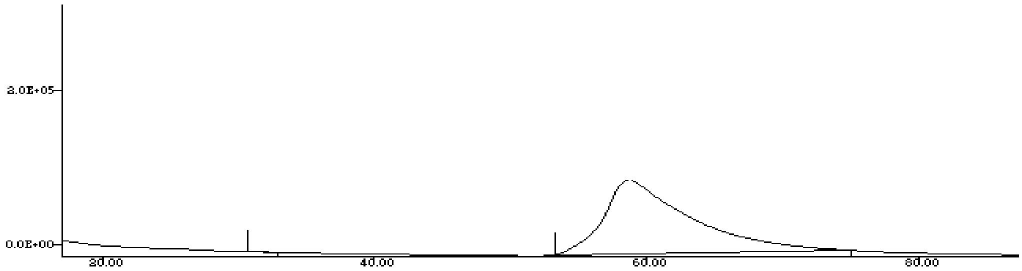

#	Name	RT	Area[uV.Sec]	Quantity
1		31.467	34118.000	0.000
2		58.600	41605038.775	0.000

Total Area of Peak = 41639156.775 [uV.Sec]

^{1}H NMR

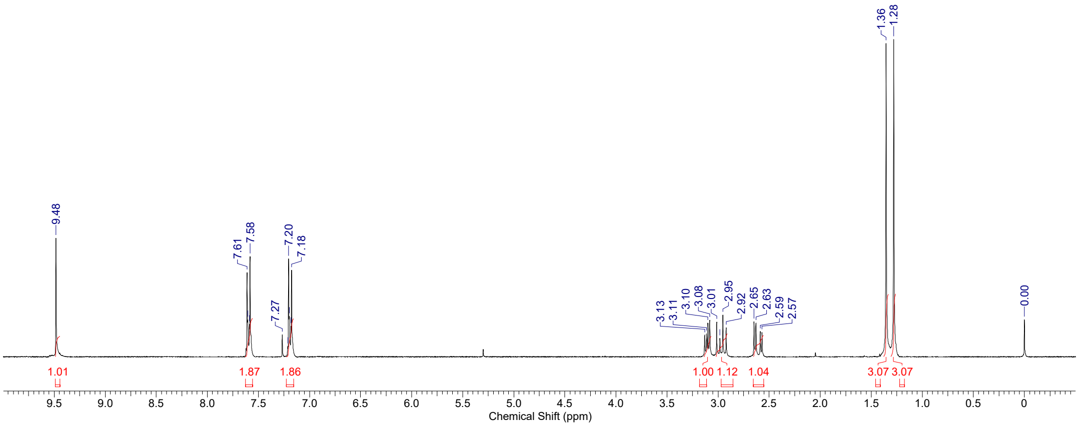

^{13}C NMR

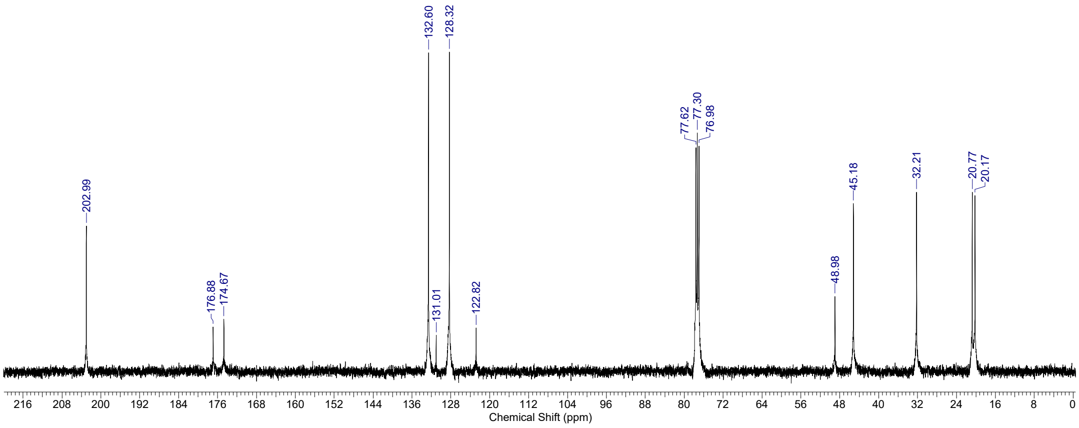

Mass

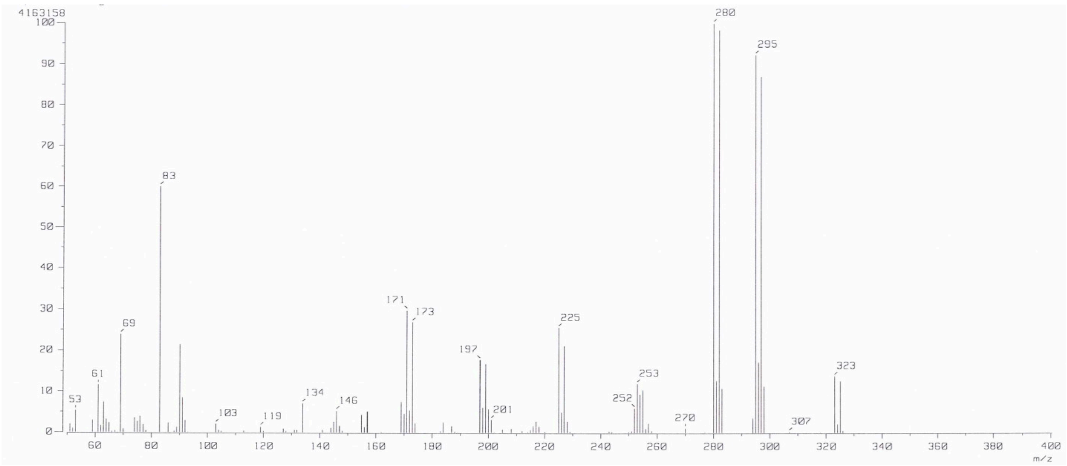

Table 2, entry 4

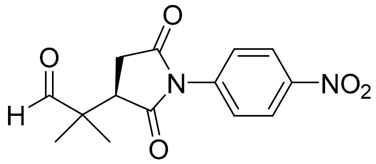

2e

Racemic

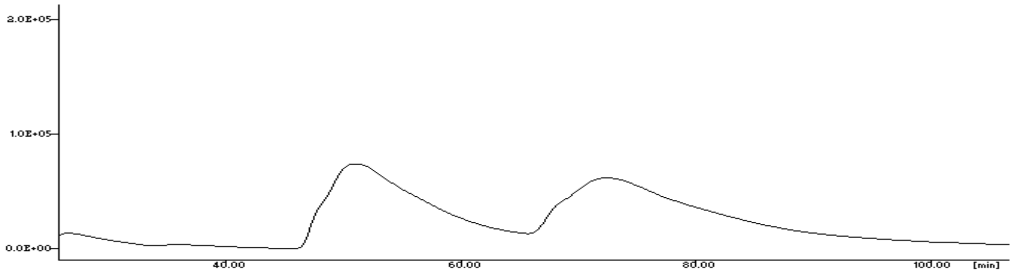

#	Name	RT	Area[uV.Sec]	Quantity
1		50.975	32694061.500	0.000
2		72.325	34471650.877	0.000

Total Area of Peak = 67165712.377 [uV.Sec]

Asymmetric

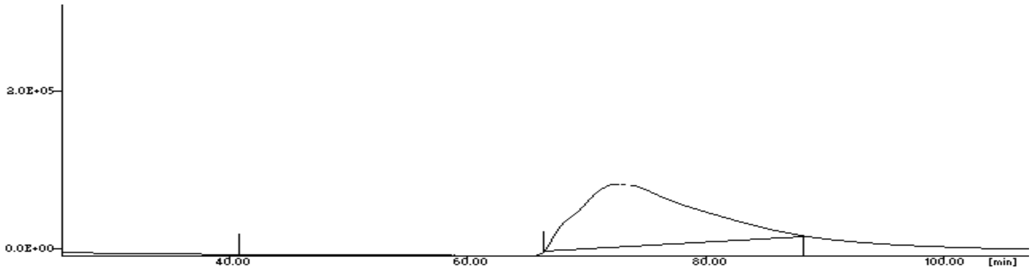

#	Name	RT	Area[uV.Sec]	Quantity
1		44.442	312501.490	0.000
2		72.600	53641693.000	0.000

Total Area of Peak = 53954194.490 [uV.Sec]

^{1}H NMR

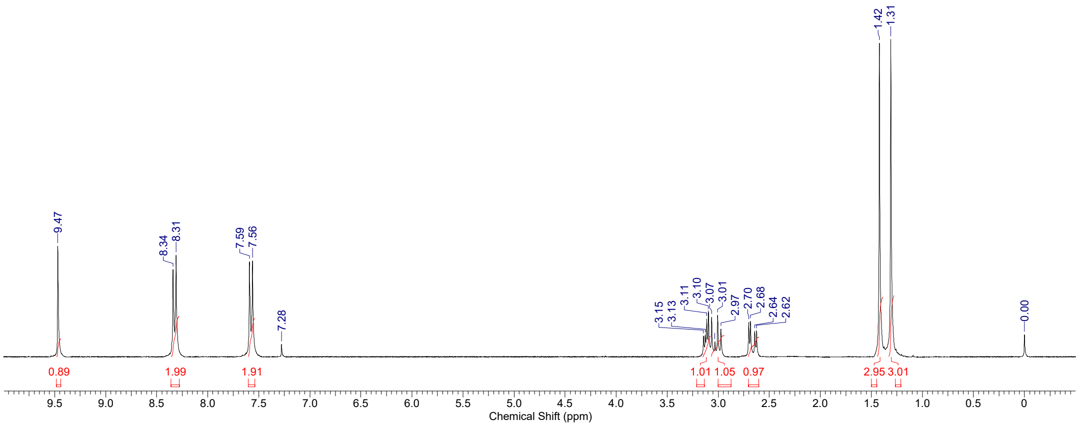

^{13}C NMR

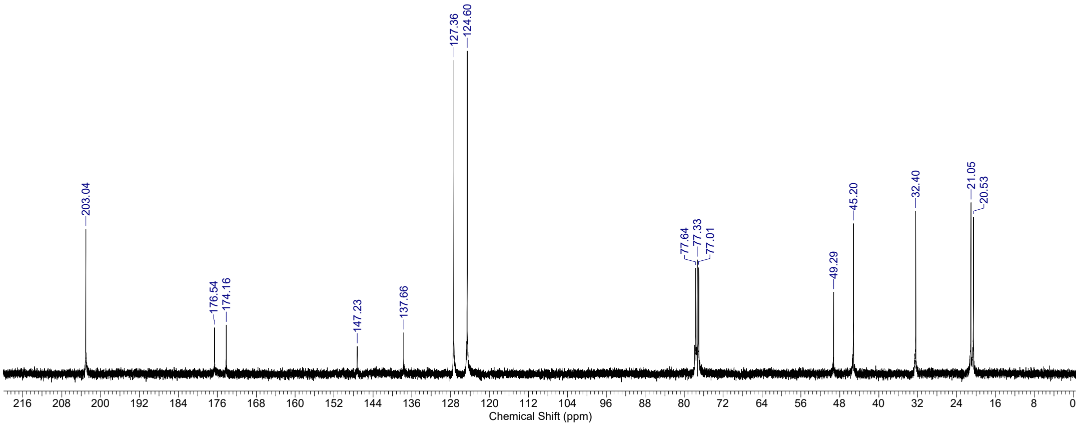

Mass

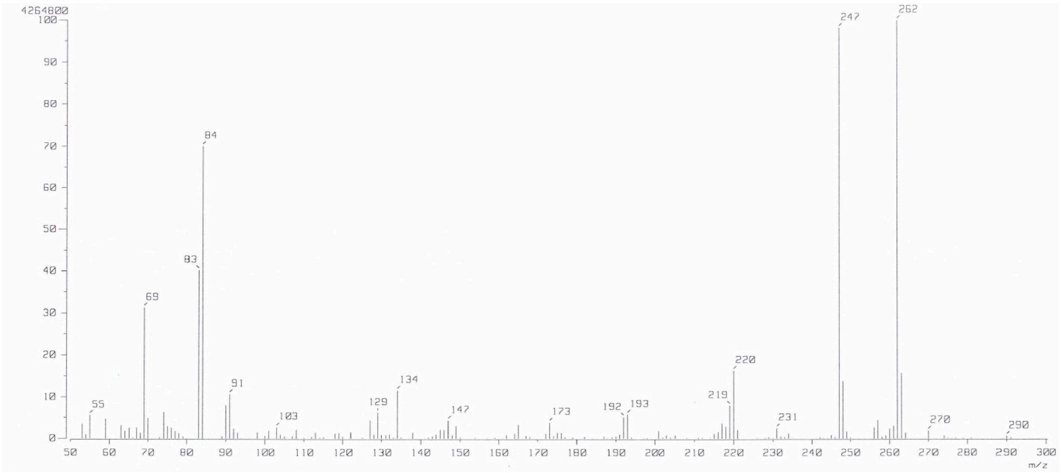

Figure 6. 1st

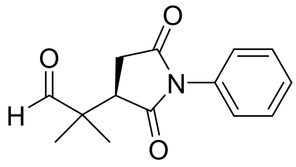

2a

Asymmetric

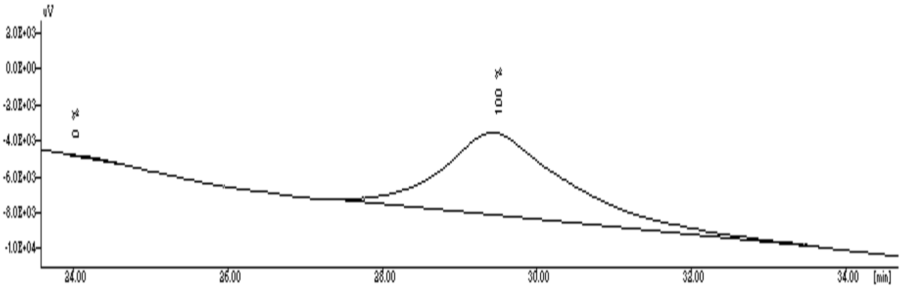

#	Name	RT	Area[uV.Sec]	Quantity
1		25.117	16883.500	0.000
2		30.267	976364.631	0.000

Figure 6. 2nd

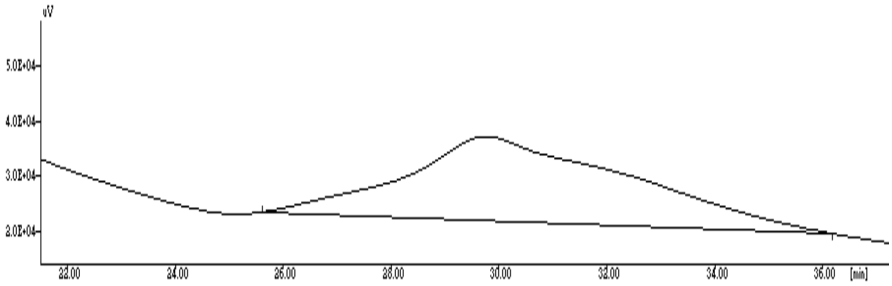

#	Name	RT	Area[uV.Sec]	Quantity
1		29.808	4406342.000	0.000

Figure 6. 3rd

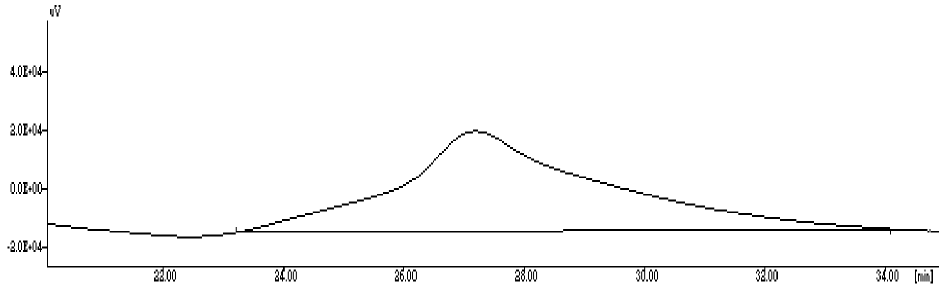

#	Name	RT	Area[uV.Sec]	Quantity
1		27.175	7898467.500	0.000

Figure 6. 4th

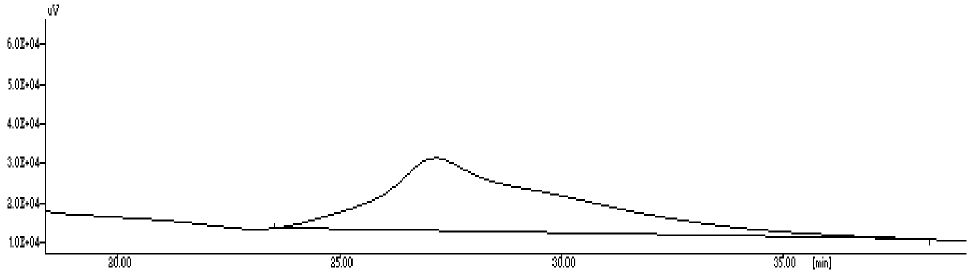

#	Name	RT	Area[uV.Sec]	Quantity
1		29.808	4406342.000	0.000

Figure 7.

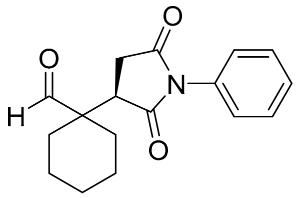

2f

Racemic

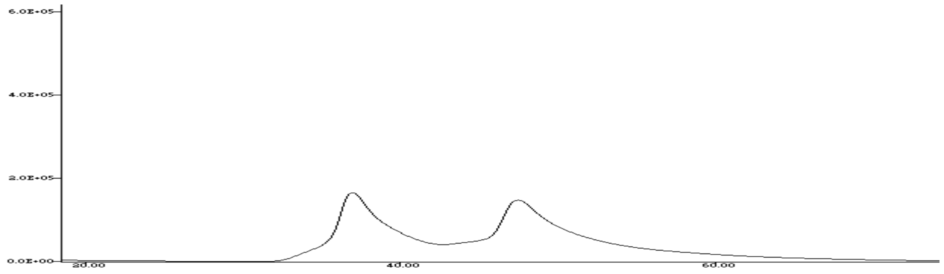

#	Name	RT	Area[uV.Sec]	Quantity
1		36.900	24715311.000	0.000
2		47.400	24489120.000	0.000

Total Area of Peak = 49204431.000 [uV.Sec]

Asymmetric

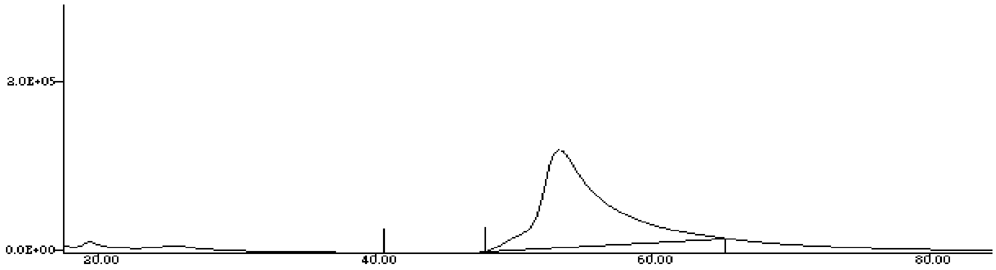

#	Name	RT	Area[uV.Sec]	Quantity
1		42.400	2822.259	0.000
2		53.233	36830060.500	0.000

Total Area of Peak = 36832882.759 [uV.Sec]

^{1}H NMR

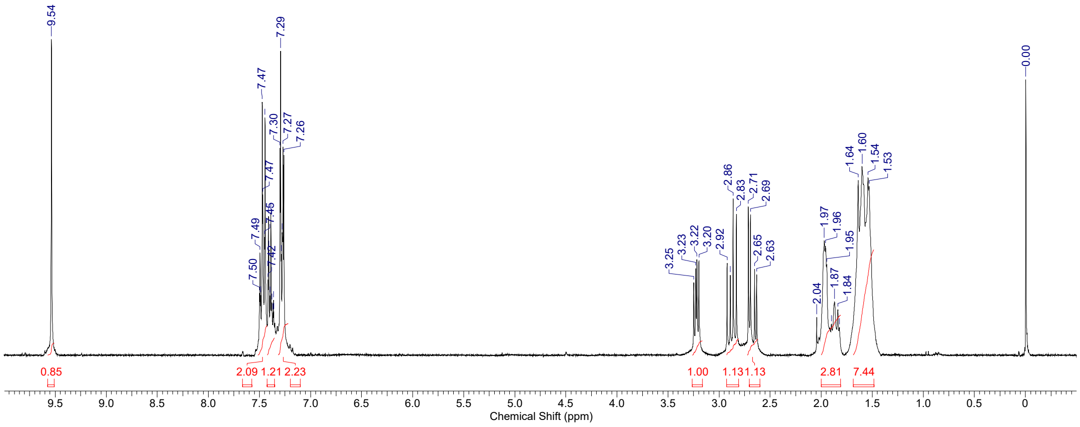
9.54
7.47
7.30
7.29
7.27
7.26
7.47
7.49
7.45
7.42
7.50
3.25
3.23
3.22
3.20
2.92
2.86
2.83
2.71
2.69
2.65
2.63
2.04
1.97
1.96
1.95
1.87
1.84
1.64
1.60
1.54
1.53
0.00
0.85
2.09
1.21
2.23
1.00
1.13
1.13
2.81
7.44
Chemical Shift (ppm)

^{13}C NMR

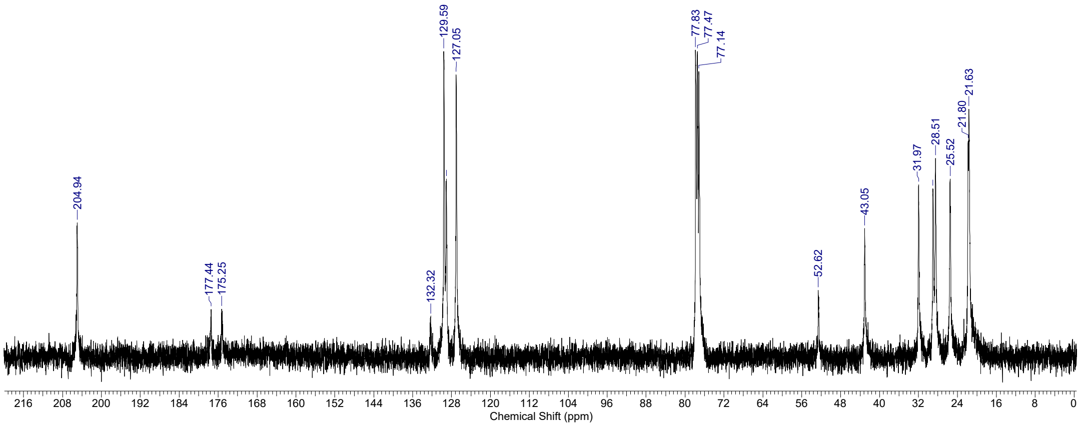
204.94
177.44
175.25
132.32
129.59
127.05
77.83
77.47
77.14
52.62
43.05
31.97
28.51
25.52
21.80
21.63
Chemical Shift (ppm)

Mass

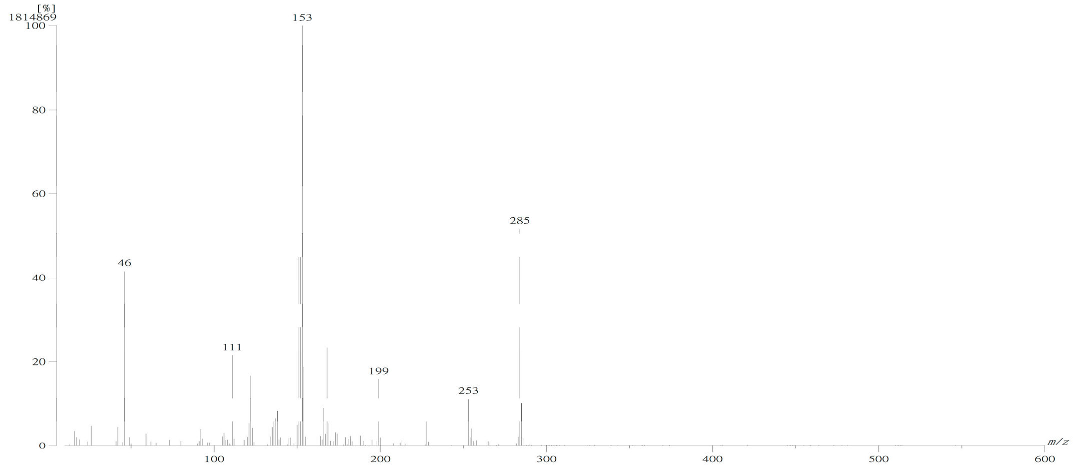
[%]
1814869
153
285
46
111
199
253
m/z

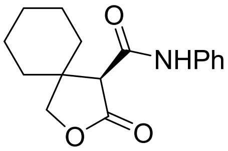

3a

Racemic

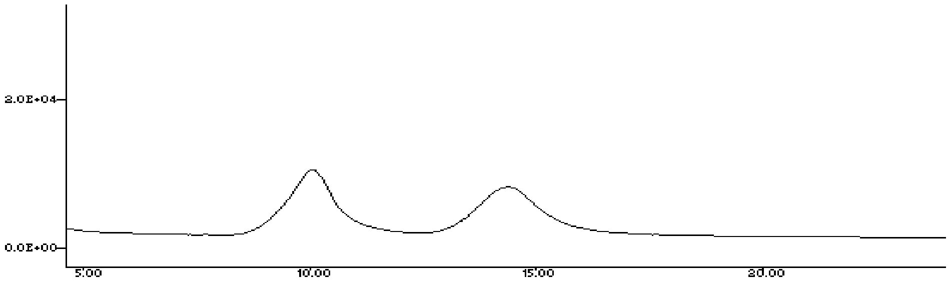

#	Name	RT	Area[uV.Sec]	Quantity
1		10.050	631497.500	0.000
2		14.342	586048.000	0.000

Total Area of Peak = 1217545.500 [uV.Sec]

Asymmetric

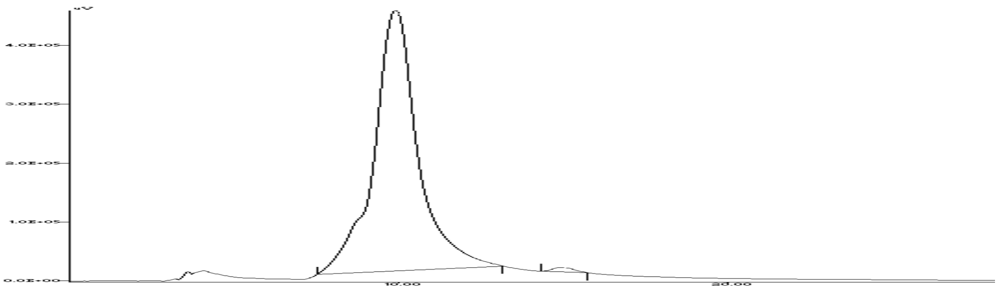

#	Name	RT	Area[uV.Sec]	Quantity
1		9.875	39721216.000	0.000
2		14.933	316721.500	0.000

Total Area of Peak = 40037937.500 [uV.Sec]

^{1}H NMR

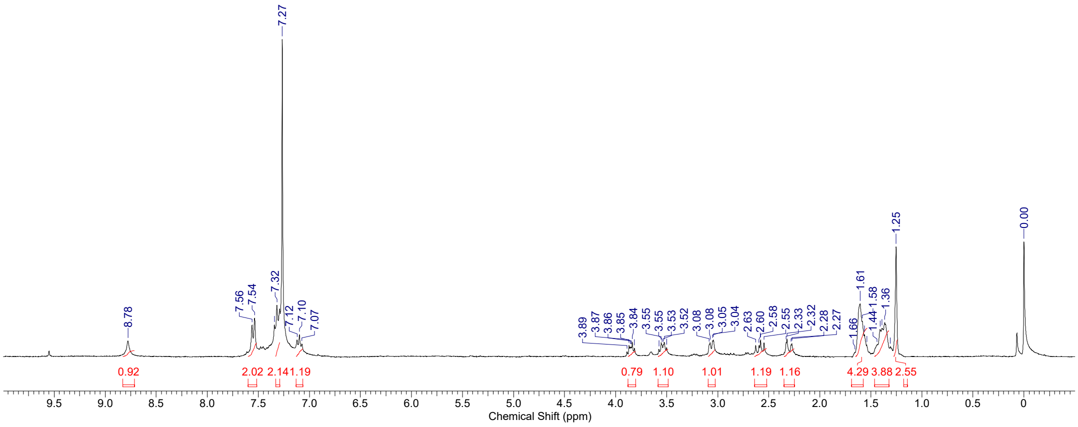

Mass

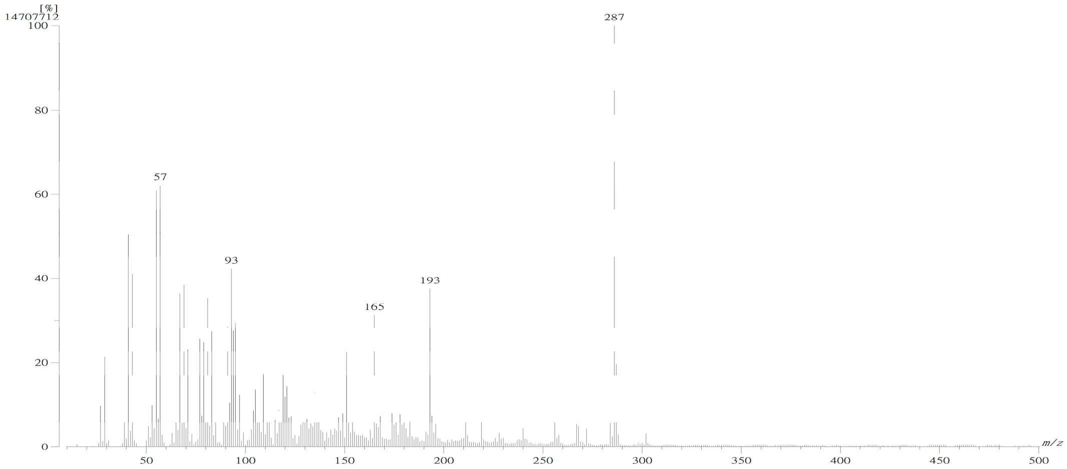

3. DFT Calculations for all Calculated Structures

Figure 2.

Isobutyraldehyde(1)

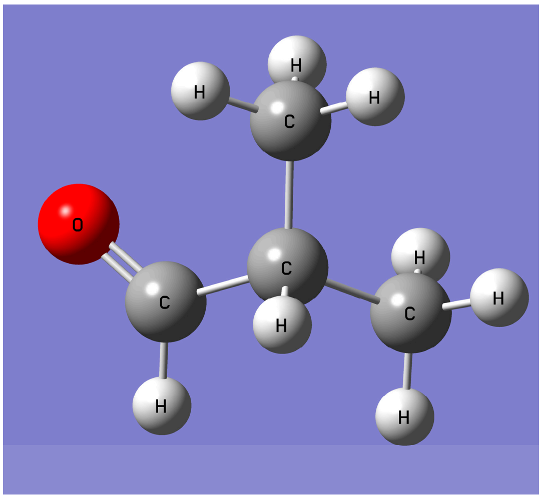

Calculation Type = FREQ

Calculation Method = RB3LYP

Basis Set = 6-31G(d,p)

Charge = 0

Spin = Singlet

Solvation = None

E(RB3LYP) = -231.1863 Hartree

RMS Gradient Norm = 1.149e-06 Hartree/Bohr

Temperature = 298.15 Kelvin

Pressure = 1 atm

Frequencies scaled by = 1

Electronic Energy (EE) = -231.1863 Hartree

Zero-point Energy Correction = 0.113857 Hartree

Thermal Correction to Energy = 0.120107 Hartree

Thermal Correction to Enthalpy = 0.121051 Hartree

Thermal Correction to Free Energy = 0.084517 Hartree

EE + Zero-point Energy = -231.07245 Hartree

EE + Thermal Energy Correction = -231.0662 Hartree

EE + Thermal Enthalpy Correction = -231.06525 Hartree

EE + Thermal Free Energy Correction = -231.10179 Hartree

E (Thermal) = 75.368 kcal/mol

Heat Capacity (Cv) = 20.996 cal/mol-kelvin

Entropy (S) = 76.892 cal/mol-kelvin

Symbolic Z-matrix:

Charge = 0 Multiplicity = 1

O	-1.93772	-0.01911	-0.18662
H	-0.98241	-1.73615	0.18648
C	0.40553	-0.0029	0.40549
C	1.50957	-0.84953	-0.20595
H	0.50964	-0.02487	1.51893
H	1.44892	-0.86091	-1.30238
H	2.49975	-0.45869	0.06243
H	1.46195	-1.89091	0.13935
C	-0.94117	-0.63596	0.10753
C	0.51426	1.43524	-0.06485
H	-0.27004	2.06351	0.37674
H	1.48383	1.86805	0.21441
H	0.421	1.51168	-1.15631

Maleimide(2)

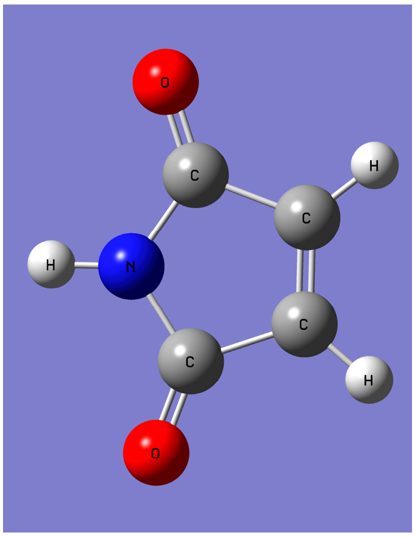

Calculation Type = FREQ

Calculation Method = RB3LYP

Basis Set = 6-31G(d,p)

Charge = 0

Spin = Singlet

Solvation = None

E(RB3LYP) = -357.44052 Hartree

RMS Gradient Norm = 1.933e-06 Hartree/Bohr

Temperature = 298.15 Kelvin

Pressure = 1 atm

Frequencies scaled by = 1

Electronic Energy (EE) = -357.44052 Hartree

Zero-point Energy Correction = 0.068699 Hartree

Thermal Correction to Energy = 0.073931 Hartree

Thermal Correction to Enthalpy = 0.074875 Hartree

Thermal Correction to Free Energy = 0.040319 Hartree

EE + Zero-point Energy = -357.37182 Hartree

EE + Thermal Energy Correction = -357.36659 Hartree

EE + Thermal Enthalpy Correction = -357.36564 Hartree

EE + Thermal Free Energy Correction = -357.4002 Hartree

E (Thermal) = 46.392 kcal/mol

Heat Capacity (Cv) = 19.398 cal/mol-kelvin

Entropy (S) = 72.729 cal/mol-kelvin

Symbolic Z-matrix:

Charge = 0 Multiplicity = 1

C	1.59514	1.16029	0.
C	3.01303	0.66578	0.
C	3.01303	-0.66578	0.
C	1.59514	-1.16029	0.
H	3.84575	1.34874	0.
H	3.84574	-1.34874	0.
O	1.22636	2.33376	0.00001
O	1.22636	-2.33376	-0.00001
N	0.7628	0.	0.
H	-0.2372	0.	0.

1b Cat.(3)

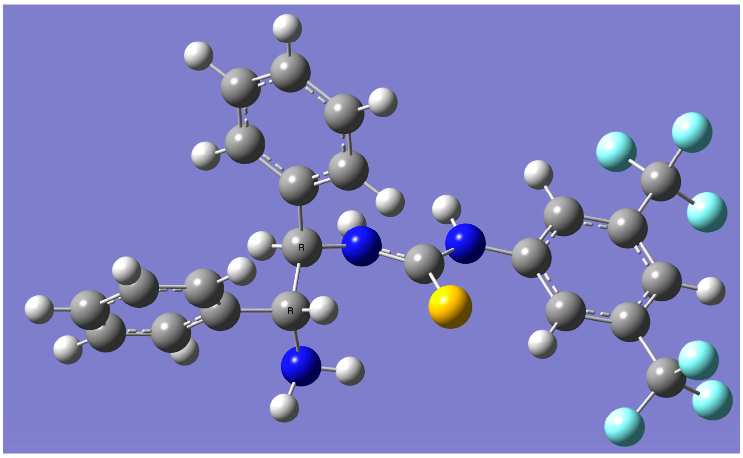

Calculation Type = FREQ

Calculation Method = RB3LYP

Basis Set = 6-31G(d,p)

Charge = 0

Spin = Singlet

Solvation = None

E(RB3LYP) = -2038.4342 Hartree

RMS Gradient Norm = 2.73e-07 Hartree/Bohr

Temperature = 298.15 Kelvin

Pressure = 1 atm

Frequencies scaled by = 1

Electronic Energy (EE) = -2038.4342 Hartree

Zero-point Energy Correction = 0.390498 Hartree

Thermal Correction to Energy = 0.419537 Hartree

Thermal Correction to Enthalpy = 0.420482 Hartree

Thermal Correction to Free Energy = 0.325242 Hartree

EE + Zero-point Energy = -2038.0437 Hartree

EE + Thermal Energy Correction = -2038.0147 Hartree

EE + Thermal Enthalpy Correction = -2038.0138 Hartree

EE + Thermal Free Energy Correction = -2038.109 Hartree

E (Thermal) = 263.264 kcal/mol

Heat Capacity (Cv) = 110.445 cal/mol-kelvin

Entropy (S) = 200.448 cal/mol-kelvin

Symbolic Z-matrix:

Charge = 0 Multiplicity = 1

C	5.35849	1.33787	-1.80781
C	4.82708	1.14638	-0.52483
C	5.69825	0.95831	0.5539
C	7.08048	0.9594	0.3547
C	7.60459	1.1496	-0.92613
C	6.73965	1.33999	-2.00723
H	4.67012	1.50429	-2.62759
H	5.29272	0.8047	1.54752
H	7.74614	0.81433	1.19782
H	8.67773	1.1541	-1.07954
H	7.1414	1.49523	-3.00239
C	3.29335	-1.35884	0.35108
C	4.2752	-2.28379	-0.02492
C	4.73342	-3.24351	0.88009
C	4.21062	-3.28913	2.17354
C	3.23141	-2.36846	2.55692
C	2.77436	-1.40784	1.65364
H	4.69377	-2.24224	-1.0248
H	5.49665	-3.95033	0.57561
H	4.56373	-4.03365	2.87769
H	2.82295	-2.39699	3.56063
H	2.01493	-0.69495	1.95103
C	3.3155	1.12842	-0.32606
C	2.81394	-0.3166	-0.65186
N	1.33778	-0.45595	-0.89719

N	2.68733	2.05148	-1.30251
H	3.24995	-0.54539	-1.62815
H	3.07939	1.35208	0.7202
H	3.151	2.9666	-1.25754
H	1.69517	2.17017	-1.06525
H	1.14702	-1.08786	-1.67474
C	0.25972	0.03025	-0.23611
C	-2.73205	0.98572	0.09707
C	-4.09709	1.1922	0.26486
C	-5.03531	0.22018	-0.05727
C	-4.57426	-0.9948	-0.54801
C	-3.217	-1.22889	-0.71787
C	-2.27072	-0.24091	-0.40093
H	-2.03806	1.77115	0.34469
H	-6.0936	0.41447	0.04014
H	-2.90295	-2.18378	-1.12204
N	-0.92225	-0.55008	-0.67028
S	0.33444	1.23285	1.01256
H	-0.81622	-1.3929	-1.23506
F	-5.79041	-2.88065	0.25434
F	-5.05699	-2.91949	-1.82873
F	-6.75108	-1.57598	-1.24681
F	-3.69111	3.50442	0.54004
F	-5.8118	2.80855	0.3756
F	-4.65299	2.43695	2.21884
C	-5.54204	-2.08849	-0.84265
C	-4.55983	2.48514	0.84774

H_2O

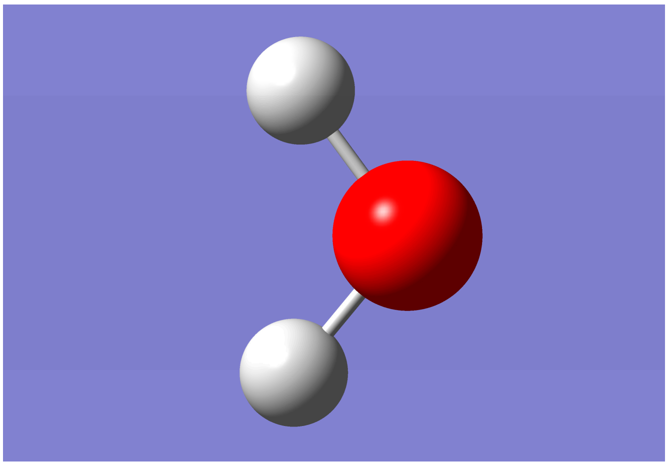

Calculation Type = FREQ

Calculation Method = RB3LYP

Basis Set = 6-31G(d,p)

Charge = 0

Spin = Singlet

Solvation = None

E(RB3LYP) = -75.973965 Hartree

RMS Gradient Norm = 0.000115305 Hartree/Bohr

Temperature = 298.15 Kelvin

Pressure = 1 atm

Frequencies scaled by = 1

Electronic Energy (EE) = -75.973965 Hartree

Zero-point Energy Correction = 0.019746 Hartree

Thermal Correction to Energy = 0.022581 Hartree

Thermal Correction to Enthalpy = 0.023525 Hartree

Thermal Correction to Free Energy = 0.002 Hartree

EE + Zero-point Energy = -75.954218 Hartree

EE + Thermal Energy Correction = -75.951384 Hartree

EE + Thermal Enthalpy Correction = -75.95044 Hartree

EE + Thermal Free Energy Correction = -75.971965 Hartree

E (Thermal) = 14.17 kcal/mol

Heat Capacity (Cv) = 5.999 cal/mol-kelvin

Entropy (S) = 45.304 cal/mol-kelvin

Symbolic Z-matrix:

Charge = 0 Multiplicity = 1

O -0.0579 2.33917 -0.16269

H 0.9021 2.33917 -0.16269

H -0.37836 3.24411 -0.16269

TS

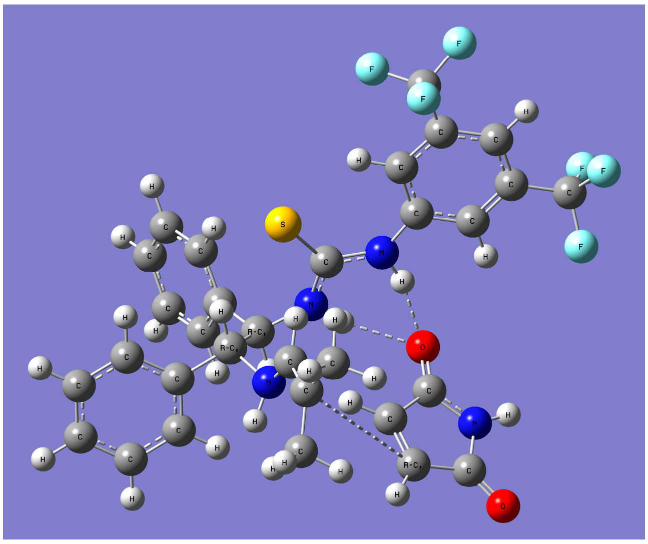

Calculation Type = FREQ

Calculation Method = RB3LYP

Basis Set = 6-31G(d,p)

Charge = 0

Spin = Singlet

Solvation = None

E(RB3LYP) = -2551.0871 Hartree

RMS Gradient Norm = 4.11e-07 Hartree/Bohr

Temperature = 298.15 Kelvin

Pressure = 1 atm

Frequencies scaled by = 1

Electronic Energy (EE) = -2551.0871 Hartree

Zero-point Energy Correction = 0.551515 Hartree

Thermal Correction to Energy = 0.592901 Hartree

Thermal Correction to Enthalpy = 0.593846 Hartree

Thermal Correction to Free Energy = 0.468285 Hartree

EE + Zero-point Energy = -2550.5356 Hartree

EE + Thermal Energy Correction = -2550.4942 Hartree

EE + Thermal Enthalpy Correction = -2550.4933 Hartree

EE + Thermal Free Energy Correction = -2550.6188 Hartree

E (Thermal) = 372.051 kcal/mol

Heat Capacity (Cv) = 153.154 cal/mol-kelvin

Entropy (S) = 264.265 cal/mol-kelvin

Symbolic Z-matrix:

Charge = 0 Multiplicity = 1

C	5.37602	0.57255	0.13239
C	4.58852	-0.4647	0.65675
C	5.22523	-1.57803	1.21612
C	6.61882	-1.6545	1.25587
C	7.3942	-0.61565	0.73765
C	6.76879	0.49963	0.1748
H	4.90214	1.44068	-0.31631
H	4.62603	-2.38756	1.61669
H	7.09726	-2.52375	1.69171
H	8.47578	-0.67426	0.76986
H	7.36469	1.30805	-0.23329
C	3.07802	-1.57727	-1.66749
C	4.00068	-1.29683	-2.68556
C	4.53106	-2.31758	-3.47533
C	4.13998	-3.63987	-3.25913
C	3.21847	-3.92887	-2.25035
C	2.69023	-2.90824	-1.45784
H	4.31385	-0.27221	-2.85649
H	5.24493	-2.08024	-4.25573
H	4.54756	-4.4361	-3.87133
H	2.90592	-4.95255	-2.07824

H	1.96749	-3.1348	-0.68589
C	3.06977	-0.37141	0.63869
C	2.54146	-0.41496	-0.83161
N	1.06486	-0.23863	-0.93127
N	2.6361	0.91997	1.27578
H	2.92913	0.50072	-1.29052
H	2.63788	-1.2062	1.19017
H	0.78975	0.506	-1.57595
C	0.03522	-0.85826	-0.29362
C	-2.96022	-1.70535	0.39432
C	-4.32442	-1.86924	0.61564
C	-5.26805	-0.98264	0.11442
C	-4.81312	0.10339	-0.62528
C	-3.45975	0.30004	-0.84787
C	-2.50395	-0.6033	-0.34361
H	-2.25995	-2.42436	0.78613
H	-6.32295	-1.13153	0.29326
H	-3.13638	1.16903	-1.40831
N	-1.1721	-0.30019	-0.65779
S	0.20123	-2.14907	0.8641
H	-1.08496	0.54178	-1.23937
F	-6.9374	1.12313	-0.49143
F	-6.17629	0.63918	-2.50724
F	-5.25227	2.29816	-1.38078
F	-3.92513	-4.07187	1.37743
F	-4.82966	-2.65282	2.80856
F	-6.04535	-3.4028	1.12405
C	-5.79221	1.03675	-1.2464
C	-4.77712	-2.99896	1.477
C	-0.08855	3.1443	-1.35866
C	0.09261	5.37327	-0.74436

C	1.00825	4.54202	0.11701
O	-0.45908	2.1194	-1.96482
H	1.4294	2.39401	0.1837
H	3.43346	1.53357	1.45189
C	1.70364	0.87334	2.34793
C	1.59899	1.75253	3.35788
C	2.52885	2.93284	3.55985
C	0.48255	1.62411	4.37553
H	1.00075	0.05123	2.26528
H	2.02982	3.87886	3.29957
H	2.82585	3.00533	4.61401
H	3.44684	2.86017	2.96515
H	0.89082	1.52648	5.39111
H	-0.16261	2.51466	4.36818
H	-0.14253	0.74903	4.17088
C	0.90833	3.25372	-0.23067
H	1.61847	4.98031	0.88925
O	-0.1085	6.57853	-0.74394
N	-0.53034	4.4275	-1.61151
H	-1.21607	4.67095	-2.31721

TS(Gas)

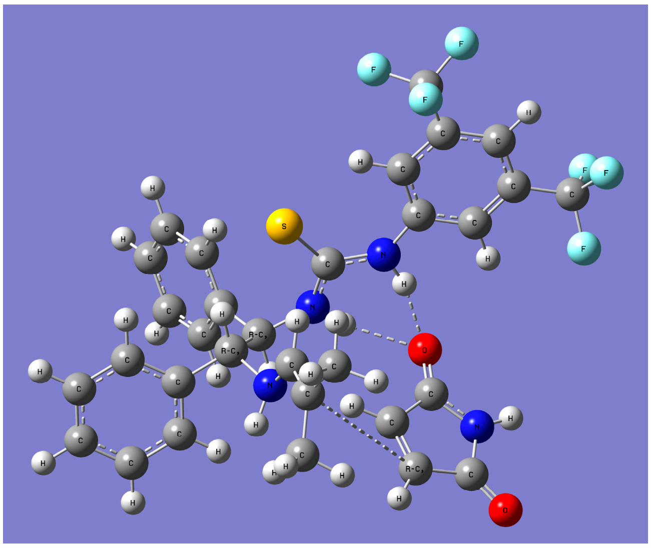

Calculation Type = FREQ

Calculation Method = RB3LYP

Basis Set = 6-31G(d,p)

Charge = 0

Spin = Singlet

Solvation = None

E(RB3LYP) = -2551.0871 Hartree

RMS Gradient Norm = 4.11e-07 Hartree/Bohr

Temperature = 298.15 Kelvin

Pressure = 1 atm

Frequencies scaled by = 1

Electronic Energy (EE) = -2551.0871 Hartree

Zero-point Energy Correction = 0.551515 Hartree

Thermal Correction to Energy = 0.592901 Hartree

Thermal Correction to Enthalpy = 0.593846 Hartree

Thermal Correction to Free Energy = 0.468285 Hartree

EE + Zero-point Energy = -2550.5356 Hartree

EE + Thermal Energy Correction = -2550.4942 Hartree

EE + Thermal Enthalpy Correction = -2550.4933 Hartree

EE + Thermal Free Energy Correction = -2550.6188 Hartree

E (Thermal) = 372.051 kcal/mol

Heat Capacity (Cv) = 153.154 cal/mol-kelvin

Entropy (S) = 264.265 cal/mol-kelvin

Symbolic Z-matrix:

Charge = 0 Multiplicity = 1

C	5.37602	0.57255	0.13239
C	4.58852	-0.4647	0.65675
C	5.22523	-1.57803	1.21612
C	6.61882	-1.6545	1.25587
C	7.3942	-0.61565	0.73765
C	6.76879	0.49963	0.1748
H	4.90214	1.44068	-0.31631
H	4.62603	-2.38756	1.61669
H	7.09726	-2.52375	1.69171
H	8.47578	-0.67426	0.76986
H	7.36469	1.30805	-0.23329
C	3.07802	-1.57727	-1.66749
C	4.00068	-1.29683	-2.68556
C	4.53106	-2.31758	-3.47533
C	4.13998	-3.63987	-3.25913
C	3.21847	-3.92887	-2.25035
C	2.69023	-2.90824	-1.45784
H	4.31385	-0.27221	-2.85649
H	5.24493	-2.08024	-4.25573
H	4.54756	-4.4361	-3.87133
H	2.90592	-4.95255	-2.07824
H	1.96749	-3.1348	-0.68589

C	3.06977	-0.37141	0.63869
C	2.54146	-0.41496	-0.83161
N	1.06486	-0.23863	-0.93127
N	2.6361	0.91997	1.27578
H	2.92913	0.50072	-1.29052
H	2.63788	-1.2062	1.19017
H	0.78975	0.506	-1.57595
C	0.03522	-0.85826	-0.29362
C	-2.96022	-1.70535	0.39432
C	-4.32442	-1.86924	0.61564
C	-5.26805	-0.98264	0.11442
C	-4.81312	0.10339	-0.62528
C	-3.45975	0.30004	-0.84787
C	-2.50395	-0.6033	-0.34361
H	-2.25995	-2.42436	0.78613
H	-6.32295	-1.13153	0.29326
H	-3.13638	1.16903	-1.40831
N	-1.1721	-0.30019	-0.65779
S	0.20123	-2.14907	0.8641
H	-1.08496	0.54178	-1.23937
F	-6.9374	1.12313	-0.49143
F	-6.17629	0.63918	-2.50724
F	-5.25227	2.29816	-1.38078
F	-3.92513	-4.07187	1.37743
F	-4.82966	-2.65282	2.80856
F	-6.04535	-3.4028	1.12405
C	-5.79221	1.03675	-1.2464
C	-4.77712	-2.99896	1.477
C	-0.08855	3.1443	-1.35866
C	0.09261	5.37327	-0.74436
C	1.00825	4.54202	0.11701

O	-0.45908	2.1194	-1.96482
H	1.4294	2.39401	0.1837
H	3.43346	1.53357	1.45189
C	1.70364	0.87334	2.34793
C	1.59899	1.75253	3.35788
C	2.52885	2.93284	3.55985
C	0.48255	1.62411	4.37553
H	1.00075	0.05123	2.26528
H	2.02982	3.87886	3.29957
H	2.82585	3.00533	4.61401
H	3.44684	2.86017	2.96515
H	0.89082	1.52648	5.39111
H	-0.16261	2.51466	4.36818
H	-0.14253	0.74903	4.17088
C	0.90833	3.25372	-0.23067
H	1.61847	4.98031	0.88925
O	-0.1085	6.57853	-0.74394
N	-0.53034	4.4275	-1.61151
H	-1.21607	4.67095	-2.31721

TS(Gas)+3H_2O

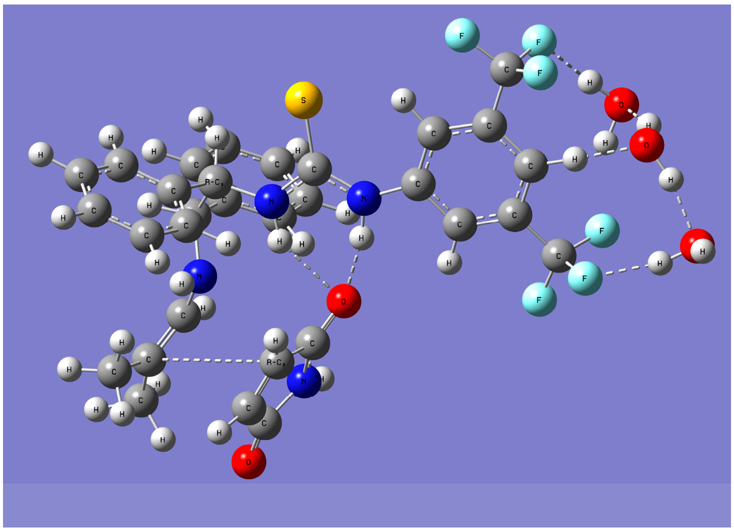

Calculation Type = FREQ

Calculation Method = RB3LYP

Basis Set = 6-31G(d,p)

Charge = 0

Spin = Singlet

Solvation = None

E(RB3LYP) = -2779.1179 Hartree

RMS Gradient Norm = 2.45e-07 Hartree/Bohr

Temperature = 298.15 Kelvin

Pressure = 1 atm

Frequencies scaled by = 1

Electronic Energy (EE) = -2779.1179 Hartree

Zero-point Energy Correction = 0.626939 Hartree

Thermal Correction to Energy = 0.675005 Hartree

Thermal Correction to Enthalpy = 0.675949 Hartree

Thermal Correction to Free Energy = 0.539008 Hartree

EE + Zero-point Energy = -2778.4909 Hartree

EE + Thermal Energy Correction = -2778.4429 Hartree

EE + Thermal Enthalpy Correction = -2778.4419 Hartree

EE + Thermal Free Energy Correction = -2778.5789 Hartree

E (Thermal) = 423.572 kcal/mol

Heat Capacity (Cv) = 178.166 cal/mol-kelvin

Entropy (S) = 288.218 cal/mol-kelvin

Symbolic Z-matrix:

Charge = 0 Multiplicity = 1

C	-2.31938	0.37882	2.66547
C	-3.31181	-0.58515	2.42974
C	-3.89683	-1.23303	3.52691
C	-3.49912	-0.93829	4.83143
C	-2.50971	0.01964	5.05749
C	-1.9255	0.67573	3.97201
H	-1.84194	0.8968	1.84508
H	-4.67206	-1.97326	3.35695
H	-3.96179	-1.4522	5.66585
H	-2.19617	0.25231	6.06834
H	-1.15222	1.41643	4.1411
C	-3.38857	-2.54405	-0.92274
C	-4.36022	-3.5409	-0.7552
C	-5.03558	-4.06732	-1.85587
C	-4.74826	-3.603	-3.14291
C	-3.77294	-2.62082	-3.31971
C	-3.09469	-2.0968	-2.21529
H	-4.57735	-3.91709	0.23961
H	-5.77763	-4.84414	-1.71236
H	-5.27	-4.01422	-3.99898
H	-3.52791	-2.27336	-4.31698
H	-2.30668	-1.3666	-2.35207
C	-3.75747	-0.99895	1.03466
C	-2.72449	-1.93348	0.31619

N	-1.5568	-1.11951	-0.00133
N	-3.99603	0.17174	0.12485
H	-2.4152	-2.73186	0.99655
H	-4.68468	-1.57904	1.13908
H	-1.7527	-0.12376	-0.09662
C	-0.26252	-1.55888	0.00278
C	2.87814	-1.56139	-0.30529
C	4.24714	-1.35356	-0.44264
C	4.79316	-0.08384	-0.57731
C	3.92731	1.00423	-0.55688
C	2.5595	0.82805	-0.41936
C	2.00705	-0.46152	-0.29611
H	2.48574	-2.56262	-0.22306
H	5.85461	0.05294	-0.72381
H	1.90901	1.69442	-0.42692
N	0.61223	-0.51564	-0.19573
S	0.13163	-3.22474	0.23546
H	0.16527	0.41036	-0.23629
F	5.63957	2.41502	-1.36285
F	4.78111	2.899	0.61328
F	3.5699	3.2508	-1.19867
F	4.54523	-3.66009	-0.87544
F	6.30097	-2.29632	-1.12504
F	5.56954	-2.8198	0.89203
C	4.47774	2.38513	-0.62791
C	5.15943	-2.5316	-0.39018
C	-1.90819	2.42179	-0.64265
C	-3.56717	2.8898	-2.18279
C	-3.71101	3.83217	-1.02093
O	-0.94361	1.8759	-0.06898
C	-2.52144	2.0814	-1.96805

O	-4.52373	4.7308	-0.83442
N	-2.64252	3.47957	-0.14791
H	-2.48105	3.91511	0.75255
H	-2.1336	1.29717	-2.59659
H	-4.19477	-0.16628	-0.82146
C	-4.89222	1.17144	0.57129
C	-5.93286	1.69974	-0.10337
C	-6.44758	1.18882	-1.43649
C	-6.70218	2.86538	0.48213
H	-4.65616	1.53938	1.56423
H	-7.47707	0.82573	-1.31338
H	-6.47934	1.99301	-2.18216
H	-5.85078	0.36678	-1.84324
H	-6.45344	3.78979	-0.05872
H	-7.78351	2.69933	0.40122
H	-6.45176	3.01745	1.5376
H	-4.23361	2.91908	-3.02779
O	8.13333	-0.78187	1.99614
H	7.17861	-0.69147	2.04028
H	8.36615	-1.71281	1.96878
O	8.25845	1.26837	0.73659
H	7.30374	1.35877	0.78073
H	8.49127	0.33743	0.70923
O	7.66637	3.37291	-0.68953
H	6.71166	3.46331	-0.64538
H	7.89919	2.44198	-0.71689

TS(Water) +3H_2O

Calculation Type = FREQ

Calculation Method = RB3LYP

Basis Set = 6-31G(d,p)

Charge = 0

Spin = Singlet

Solvation = scrf=solvent=water

E(RB3LYP) = -2779.171 Hartree

RMS Gradient Norm = 3.9e-07 Hartree/Bohr

Temperature = 298.15 Kelvin

Pressure = 1 atm

Frequencies scaled by = 1

Electronic Energy (EE) = -2779.171 Hartree

Zero-point Energy Correction = 0.626631 Hartree

Thermal Correction to Energy = 0.674753 Hartree

Thermal Correction to Enthalpy = 0.675697 Hartree

Thermal Correction to Free Energy = 0.539009 Hartree

EE + Zero-point Energy = -2778.5444 Hartree

EE + Thermal Energy Correction = -2778.4963 Hartree

EE + Thermal Enthalpy Correction = -2778.4953 Hartree

EE + Thermal Free Energy Correction = -2778.632 Hartree

E (Thermal) = 423.414 kcal/mol

Heat Capacity (Cv) = 177.86 cal/mol-kelvin

Entropy (S) = 287.685 cal/mol-kelvin

Symbolic Z-matrix:

Charge = 0 Multiplicity = 1

C	-2.73659	0.12686	2.5497
C	-3.7398	-0.78489	2.18601
C	-4.40162	-1.5037	3.19167
C	-4.06692	-1.32521	4.53593

C	-3.0669	-0.41648	4.89136
C	-2.40685	0.30889	3.89517
H	-2.21735	0.6985	1.79189
H	-5.18469	-2.20415	2.92017
H	-4.58888	-1.88829	5.30084
H	-2.80746	-0.27136	5.93355
H	-1.6328	1.01815	4.16514
C	-3.62157	-2.59368	-1.24001
C	-4.62985	-3.56852	-1.19778
C	-5.21026	-4.03928	-2.37627
C	-4.79014	-3.5396	-3.61389
C	-3.78231	-2.5745	-3.66309
C	-3.19775	-2.10525	-2.48175
H	-4.95685	-3.96381	-0.24127
H	-5.98498	-4.79584	-2.33044
H	-5.24095	-3.90442	-4.52928
H	-3.44556	-2.18852	-4.61829
H	-2.40019	-1.37374	-2.52581
C	-4.09942	-1.07401	0.73402
C	-3.05912	-2.05723	0.0782
N	-1.82315	-1.29462	-0.09544
N	-4.11027	0.14436	-0.11205
H	-2.86965	-2.8871	0.76265
H	-5.07056	-1.58722	0.71698
H	-1.98178	-0.28518	-0.21422
C	-0.56307	-1.78222	-0.02469
C	2.63732	-1.86873	-0.12341
C	4.01142	-1.65104	-0.22008
C	4.57606	-0.38658	-0.35953
C	3.69374	0.69474	-0.33753
C	2.32695	0.51765	-0.23573

C	1.7671	-0.77173	-0.15977
H	2.24809	-2.86793	-0.0149
H	5.67039	-0.247	-0.45861
H	1.66878	1.37229	-0.20065
N	0.37115	-0.78723	-0.10632
S	-0.22475	-3.4884	0.16155
H	-0.02433	0.16508	-0.12554
F	4.97022	2.37496	-1.46876
F	5.15501	2.22915	0.72009
F	3.28126	3.03864	-0.18235
F	4.28591	-4.00347	-0.03387
F	5.93034	-2.83701	-0.97782
F	5.59274	-2.69726	1.20028
C	4.24309	2.07103	-0.31916
C	4.93658	-2.79902	-0.03664
C	-1.09748	2.85369	-0.27946
C	-0.73421	5.09489	-0.69915
C	-2.22229	4.83231	-0.60882
O	-0.88777	1.6474	-0.05512
C	-0.08	3.94517	-0.50767
O	-3.1588	5.61844	-0.72249
N	-2.33594	3.44849	-0.35391
H	-3.22592	2.94463	-0.23966
H	0.98267	3.76161	-0.49544
H	-4.19964	-0.08033	-1.10506
C	-4.77266	1.313	0.3046
C	-5.25614	2.2977	-0.48416
C	-5.25876	2.24753	-2.00172
C	-5.82986	3.56785	0.12179
H	-4.82096	1.42012	1.3831
H	-6.25785	2.4976	-2.38122

H	-4.55872	2.9837	-2.4212
H	-4.98751	1.26391	-2.39796
H	-5.2237	4.43914	-0.16522
H	-6.85211	3.73984	-0.24102
H	-5.85585	3.51163	1.21558
H	-0.33287	6.07658	-0.88637
O	6.4475	-0.07721	2.13977
H	5.77729	0.62394	1.91901
H	5.99788	-0.95168	1.97726
O	7.48507	0.03124	-0.32121
H	7.65191	1.00733	-0.56035
H	7.30818	0.00937	0.67684
O	7.73605	2.6156	-1.01964
H	6.77041	2.74508	-1.22158
H	8.20129	2.64861	-1.89737

TS(Water) +4H_2O

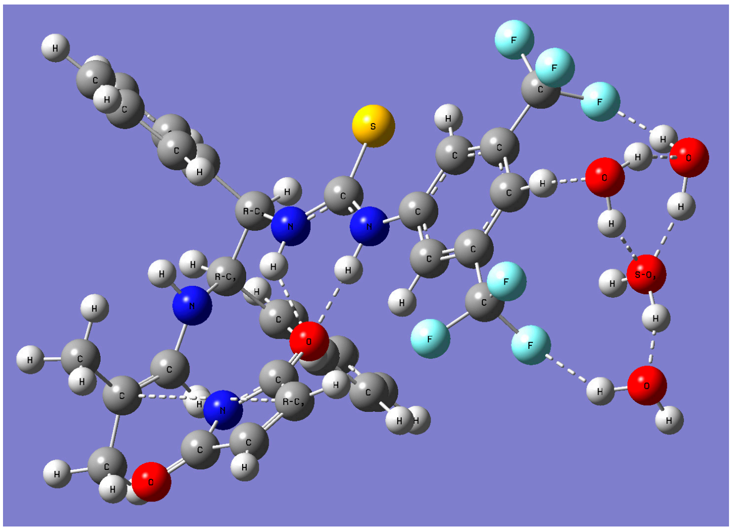

Calculation Type = FREQ

Calculation Method = RB3LYP

Basis Set = 6-31G(d,p)

Charge = 0

Spin = Singlet

Solvation = scrf=solvent=water

E(RB3LYP) = -2855.1919 Hartree

RMS Gradient Norm = 3.93e-07 Hartree/Bohr

Temperature = 298.15 Kelvin

Pressure = 1 atm

Frequencies scaled by = 1

Electronic Energy (EE) = -2855.1919 Hartree

Zero-point Energy Correction = 0.652279 Hartree

Thermal Correction to Energy = 0.702153 Hartree

Thermal Correction to Enthalpy = 0.703097 Hartree

Thermal Correction to Free Energy = 0.563221 Hartree

EE + Zero-point Energy = -2854.5396 Hartree

EE + Thermal Energy Correction = -2854.4897 Hartree

EE + Thermal Enthalpy Correction = -2854.4888 Hartree

EE + Thermal Free Energy Correction = -2854.6287 Hartree

E (Thermal) = 440.608 kcal/mol

Heat Capacity (Cv) = 184.729 cal/mol-kelvin

Entropy (S) = 294.394 cal/mol-kelvin

Symbolic Z-matrix:

Charge = 0 Multiplicity = 1

C	-2.82374	0.34655	2.59229
C	-3.86205	-0.56515	2.34542
C	-4.49101	-1.18894	3.43239
C	-4.0898	-0.9175	4.74248
C	-3.05517	-0.00945	4.98146
C	-2.42747	0.6217	3.90359
H	-2.32836	0.8465	1.77046
H	-5.30079	-1.88823	3.25135
H	-4.58717	-1.40783	5.57121
H	-2.744	0.2079	5.9966
H	-1.62675	1.3301	4.08294
C	-3.94657	-2.62976	-0.93577
C	-4.97517	-3.57062	-0.77565
C	-5.61971	-4.11263	-1.88833
C	-5.2442	-3.71921	-3.17753
C	-4.21636	-2.78889	-3.34394
C	-3.56751	-2.24834	-2.22845
H	-5.26773	-3.88414	0.22143
H	-6.40951	-4.84205	-1.75115
H	-5.74472	-4.13932	-4.04196
H	-3.91375	-2.48576	-4.33959
H	-2.75441	-1.5455	-2.36305
C	-4.29688	-0.95399	0.93775
C	-3.31195	-2.01203	0.3122

N	-2.06718	-1.29831	0.02921
N	-4.31891	0.19587	0.00115
H	-3.11223	-2.79378	1.04851
H	-5.2795	-1.44069	1.00512
H	-2.20588	-0.29555	-0.15227
C	-0.81687	-1.81376	0.08017
C	2.35915	-2.00569	-0.2531
C	3.73675	-1.83622	-0.40419
C	4.34917	-0.58669	-0.50519
C	3.50119	0.5225	-0.44336
C	2.13153	0.38906	-0.29778
C	1.53167	-0.87918	-0.20082
H	1.93067	-2.99349	-0.19644
H	5.44256	-0.47349	-0.68999
H	1.5	1.26451	-0.26215
N	0.13723	-0.85189	-0.09456
S	-0.51412	-3.51399	0.36511
H	-0.2333	0.10887	-0.15046
F	5.14555	1.95852	-1.36659
F	4.50604	2.39121	0.70471
F	3.12744	2.82031	-0.95637
F	3.88063	-4.17216	-0.82175
F	5.67901	-2.9147	-1.24386
F	5.10644	-3.37338	0.83363
C	4.06125	1.89479	-0.53426
C	4.5789	-3.05959	-0.43955
C	-1.24962	2.78255	-0.53884
C	-0.85404	4.93435	-1.27489
C	-2.34062	4.73634	-1.06687
O	-1.05909	1.6103	-0.16539
C	-0.21946	3.79833	-0.9687

O	-3.26281	5.53031	-1.23185
N	-2.47432	3.40269	-0.62405
H	-3.36947	2.94538	-0.40358
H	0.83624	3.57827	-0.99652
H	-4.46149	-0.1023	-0.96576
C	-4.92908	1.41331	0.35215
C	-5.42519	2.34421	-0.49231
C	-5.50563	2.17055	-1.99858
C	-5.93244	3.67829	0.02925
H	-4.92011	1.60948	1.41913
H	-6.51978	2.40509	-2.34707
H	-4.81965	2.86014	-2.51032
H	-5.26627	1.15428	-2.32715
H	-5.32438	4.50192	-0.37203
H	-6.97022	3.84683	-0.28844
H	-5.89403	3.72089	1.12323
H	-0.43941	5.86817	-1.61536
O	7.88566	-1.91377	1.39852
H	7.72367	-1.38615	2.22463
H	7.12341	-2.55219	1.33998
O	7.46651	-0.21406	-0.64777
H	7.48662	0.69207	-0.17292
H	7.69162	-0.86306	0.10849
O	7.28255	1.92869	0.92393
H	6.97062	2.79559	0.49167
H	6.44551	1.56962	1.32158
O	6.45065	4.35166	0.46941
H	6.76208	4.39931	1.41606
H	5.45978	4.33143	0.5609

TS(Water) +5H_2O

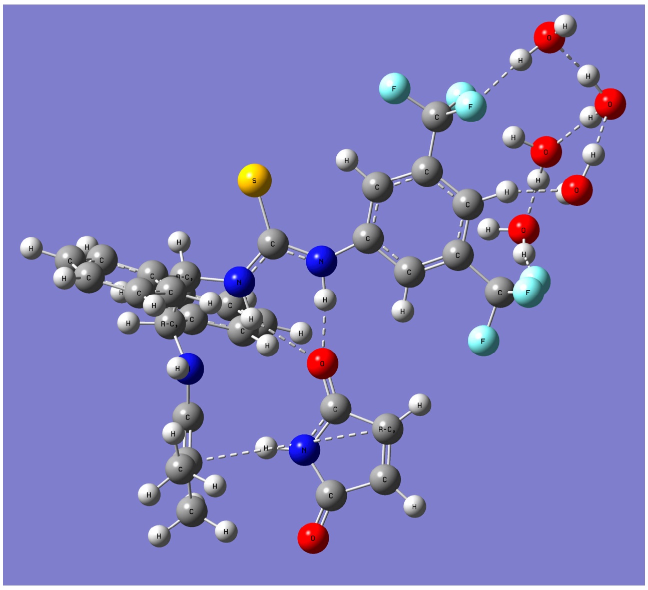

Calculation Type = FREQ

Calculation Method = RB3LYP

Basis Set = 6-31G(d,p)

Charge = 0

Spin = Singlet

Solvation = scrf=solvent=water

E(RB3LYP) = -2931.2015 Hartree

RMS Gradient Norm = 6.94e-07 Hartree/Bohr

Temperature = 298.15 Kelvin

Pressure = 1 atm

Frequencies scaled by = 1

Electronic Energy (EE) = -2931.2015 Hartree

Zero-point Energy Correction = 0.677019 Hartree

Thermal Correction to Energy = 0.729465 Hartree

Thermal Correction to Enthalpy = 0.730409 Hartree

Thermal Correction to Free Energy = 0.584429 Hartree

EE + Zero-point Energy = -2930.5245 Hartree

EE + Thermal Energy Correction = -2930.472 Hartree
EE + Thermal Enthalpy Correction = -2930.4711 Hartree
EE + Thermal Free Energy Correction = -2930.6171 Hartree
E (Thermal) = 457.746 kcal/mol
Heat Capacity (Cv) = 192.946 cal/mol-kelvin
Entropy (S) = 307.243 cal/mol-kelvin

Symbolic Z-matrix:
Charge = 0 Multiplicity = 1

C	-2.85292	0.35827	2.63683
C	-3.85632	-0.58871	2.37822
C	-4.47943	-1.23081	3.45797
C	-4.10643	-0.94326	4.77282
C	-3.10644	-0.00029	5.02383
C	-2.48524	0.64922	3.9532
H	-2.36141	0.87383	1.82202
H	-5.26261	-1.95736	3.26724
H	-4.59881	-1.44827	5.59567
H	-2.81723	0.22975	6.04266
H	-1.71154	1.38467	4.14204
C	-3.83861	-2.67278	-0.89168
C	-4.841	-3.64369	-0.74627
C	-5.45626	-4.20092	-1.86808
C	-5.07719	-3.793	-3.15166
C	-4.07515	-2.83253	-3.30344
C	-3.4557	-2.27651	-2.17892
H	-5.13616	-3.96852	0.24641
H	-6.22606	-4.95339	-1.74228
H	-5.55498	-4.22491	-4.0231
H	-3.76982	-2.51765	-4.29459
H	-2.6622	-1.54972	-2.30259

C	-4.25898	-0.99777	0.96682
C	-3.23982	-2.03757	0.36529
N	-2.00761	-1.29565	0.1013
N	-4.29415	0.14433	0.01966
H	-3.03658	-2.8116	1.10875
H	-5.23096	-1.50672	1.02173
H	-2.17118	-0.29734	-0.08132
C	-0.74629	-1.78167	0.15334
C	2.42697	-1.89155	-0.25461
C	3.78501	-1.68502	-0.49409
C	4.34589	-0.42145	-0.6662
C	3.48379	0.66895	-0.53903
C	2.13314	0.49897	-0.28973
C	1.57178	-0.78525	-0.16968
H	2.0336	-2.89013	-0.15071
H	5.41238	-0.29736	-0.91474
H	1.48329	1.35724	-0.19833
N	0.18416	-0.79233	-0.00247
S	-0.4019	-3.4766	0.41216
H	-0.20848	0.16112	-0.02563
F	5.14173	2.16842	-1.34938
F	4.35301	2.52086	0.68788
F	3.07069	2.95458	-1.06305
F	4.02334	-4.03352	-0.76349
F	5.71918	-2.72339	-1.39576
F	5.3037	-3.04547	0.74667
C	4.00733	2.05747	-0.59668
C	4.68864	-2.86551	-0.50198
C	-1.26025	2.77906	-0.55089
C	-0.97981	4.81936	-1.5956
C	-2.45326	4.57419	-1.34893

O	-1.00804	1.67885	-0.02407
C	-0.28551	3.77441	-1.13337
O	-3.42208	5.27412	-1.63351
N	-2.51381	3.32045	-0.70516
H	-3.38907	2.85694	-0.42485
H	0.77972	3.60511	-1.14323
H	-4.42806	-0.16885	-0.94382
C	-4.94605	1.34602	0.35706
C	-5.42875	2.27622	-0.4951
C	-5.44044	2.13733	-2.00727
C	-6.0051	3.58343	0.021
H	-4.98213	1.53197	1.42518
H	-6.47423	2.13158	-2.37851
H	-4.93679	2.99922	-2.46588
H	-4.94757	1.2279	-2.36389
H	-5.43504	4.42845	-0.39012
H	-7.0486	3.70043	-0.30223
H	-5.97376	3.63493	1.11451
H	-0.61573	5.7125	-2.07475
O	7.65068	-1.37927	1.01536
H	7.32021	-0.4732	1.37224
H	6.86961	-1.99351	1.05601
O	7.2328	0.04668	-1.14458
H	7.02767	0.76326	-0.47071
H	7.51959	-0.69758	-0.49498
O	6.62367	0.98424	1.37894
H	6.87655	1.97523	1.57484
H	5.64483	0.92634	1.5151
O	6.80736	3.50844	1.65478
H	6.76881	3.867	2.57993
H	5.90749	3.65099	1.25385

O	6.40225	-4.56686	-0.53906
H	7.36225	-4.56686	-0.53906
H	6.0818	-3.66192	-0.53906

TS(CH_2Cl_2) +5H_2O

Calculation Type = FREQ

Calculation Method = RB3LYP

Basis Set = 6-31G(d,p)

Charge = 0

Spin = Singlet

Solvation = scrf=solvent=dichloromethane

E(RB3LYP) = -2931.1925 Hartree

RMS Gradient Norm = 1.293e-06 Hartree/Bohr

Temperature = 298.15 Kelvin

Pressure = 1 atm

Frequencies scaled by = 1

Electronic Energy (EE) = -2931.1925 Hartree

Zero-point Energy Correction = 0.67728 Hartree

Thermal Correction to Energy = 0.729695 Hartree

Thermal Correction to Enthalpy = 0.730639 Hartree

Thermal Correction to Free Energy = 0.584691 Hartree

EE + Zero-point Energy = -2930.5152 Hartree

EE + Thermal Energy Correction = -2930.4628 Hartree

EE + Thermal Enthalpy Correction = -2930.4618 Hartree

EE + Thermal Free Energy Correction = -2930.6078 Hartree

E (Thermal) = 457.89 kcal/mol

Heat Capacity (Cv) = 192.928 cal/mol-kelvin

Entropy (S) = 307.172 cal/mol-kelvin

Symbolic Z-matrix:

Charge = 0 Multiplicity = 1

C	-3.12237	0.21549	2.56869
C	-4.02674	-0.82673	2.31271
C	-4.6002	-1.50913	3.39487
C	-4.27494	-1.1673	4.70921
C	-3.37401	-0.12898	4.95721
C	-2.80283	0.56073	3.8841
H	-2.67237	0.76188	1.75033
H	-5.30746	-2.31036	3.20641
H	-4.72764	-1.70505	5.53406
H	-3.12263	0.14308	5.97569
H	-2.10596	1.36986	4.07099
C	-3.77053	-2.92878	-0.93502
C	-4.6699	-3.99436	-0.78125
C	-5.22138	-4.62367	-1.89775
C	-4.88117	-4.19385	-3.18486
C	-3.98127	-3.13863	-3.34514
C	-3.42555	-2.51035	-2.22565
H	-4.93211	-4.33763	0.21454
H	-5.91024	-5.44986	-1.76527
H	-5.30855	-4.68261	-4.05243
H	-3.70441	-2.80715	-4.33929
H	-2.70659	-1.71061	-2.35496
C	-4.37045	-1.28988	0.90291
C	-3.24445	-2.22152	0.31594
N	-2.09645	-1.35794	0.04528
N	-4.51826	-0.16641	-0.05544
H	-2.96162	-2.96288	1.06669
H	-5.28459	-1.897	0.95581
H	-2.35857	-0.383	-0.14776

C	-0.79195	-1.71608	0.10495
C	2.3812	-1.5075	-0.24877
C	3.71768	-1.16206	-0.45637
C	4.14989	0.14589	-0.65378
C	3.17478	1.14306	-0.58794
C	1.84354	0.8381	-0.37143
C	1.41474	-0.49477	-0.22374
H	2.0923	-2.53722	-0.11087
H	5.20789	0.41528	-0.82925
H	1.10767	1.62582	-0.31955
N	0.03343	-0.63892	-0.07698
S	-0.27942	-3.35754	0.39774
H	-0.45475	0.26686	-0.12747
F	4.24932	2.85176	-1.86084
F	4.41648	2.962	0.33312
F	2.47502	3.41596	-0.65005
F	4.22439	-3.48673	-0.4178
F	5.68165	-2.10926	-1.40803
F	5.50906	-2.13703	0.78521
C	3.56841	2.57205	-0.71523
C	4.74367	-2.22844	-0.38107
C	-1.78804	2.81248	-0.54317
C	-1.67648	4.99213	-1.29771
C	-3.12585	4.59127	-1.12274
O	-1.4431	1.68118	-0.15344
C	-0.89854	3.9583	-0.96174
O	-4.14477	5.24597	-1.3232
N	-3.08413	3.25646	-0.6624
H	-3.91536	2.68384	-0.45815
H	0.1778	3.8871	-0.96041
H	-4.60743	-0.50018	-1.01727

C	-5.29007	0.96571	0.26363
C	-5.8631	1.82904	-0.60343
C	-5.85758	1.65588	-2.11184
C	-6.57167	3.07796	-0.10666
H	-5.3516	1.15968	1.32925
H	-6.88739	1.65654	-2.49268
H	-5.33435	2.49263	-2.5953
H	-5.3799	0.72702	-2.43841
H	-6.07319	3.97834	-0.49306
H	-7.61151	3.09694	-0.45991
H	-6.57818	3.12616	0.98767
H	-1.38737	5.97126	-1.64085
O	8.72488	0.10311	0.29812
H	8.09547	0.2106	1.08851
H	8.66537	-0.87553	0.00872
O	6.74511	1.39203	-0.74668
H	6.46087	1.44625	0.21386
H	7.63602	0.8738	-0.59199
O	6.56884	0.4029	1.81587
H	5.94014	0.98249	2.39802
H	6.08279	-0.41947	1.55286
O	4.81444	1.92497	2.91551
H	4.03252	1.4398	3.28657
H	4.5115	2.36794	2.07597
O	8.38874	-2.37376	-0.595
H	8.93514	-2.47995	-1.41764
H	7.4415	-2.33431	-0.8979

TS(THF) +5H_2O

Calculation Type = FREQ

Calculation Method = RB3LYP

Basis Set = 6-31G(d,p)

Charge = 0

Spin = Singlet

Solvation = scrf=solvent=thf

E(RB3LYP) = -2931.1907 Hartree

RMS Gradient Norm = 1.46e-06 Hartree/Bohr

Temperature = 298.15 Kelvin

Pressure = 1 atm

Frequencies scaled by = 1

Electronic Energy (EE) = -2931.1907 Hartree

Zero-point Energy Correction = 0.677318 Hartree

Thermal Correction to Energy = 0.729724 Hartree

Thermal Correction to Enthalpy = 0.730668 Hartree

Thermal Correction to Free Energy = 0.585007 Hartree

EE + Zero-point Energy = -2930.5134 Hartree

EE + Thermal Energy Correction = -2930.461 Hartree

EE + Thermal Enthalpy Correction = -2930.46 Hartree

EE + Thermal Free Energy Correction = -2930.6057 Hartree

E (Thermal) = 457.909 kcal/mol

Heat Capacity (Cv) = 192.946 cal/mol-kelvin

Entropy (S) = 306.568 cal/mol-kelvin

Symbolic Z-matrix:

Charge = 0 Multiplicity = 1

C	-3.13167	0.21987	2.56732
C	-4.03515	-0.82283	2.31028
C	-4.61319	-1.50222	3.39186
C	-4.29329	-1.15712	4.7066

C	-3.39325	-0.11834	4.95563
C	-2.81758	0.56846	3.88311
H	-2.67808	0.76403	1.74948
H	-5.31984	-2.3038	3.2026
H	-4.74942	-1.69271	5.53096
H	-3.14599	0.15626	5.97442
H	-2.12131	1.37792	4.07085
C	-3.76821	-2.93182	-0.93215
C	-4.66871	-3.99646	-0.77895
C	-5.2175	-4.62749	-1.89575
C	-4.87341	-4.20033	-3.18267
C	-3.97225	-3.14616	-3.34241
C	-3.41931	-2.51616	-2.22256
H	-4.93352	-4.338	0.21676
H	-5.90709	-5.45312	-1.76368
H	-5.29854	-4.69055	-4.05052
H	-3.69201	-2.81711	-4.33642
H	-2.69879	-1.7177	-2.35115
C	-4.37347	-1.28952	0.9004
C	-3.24542	-2.22221	0.31891
N	-2.09706	-1.35887	0.04986
N	-4.51868	-0.16841	-0.06133
H	-2.96439	-2.9621	1.07183
H	-5.28772	-1.89666	0.9515
H	-2.3591	-0.38446	-0.14514
C	-0.79235	-1.71677	0.11361
C	2.38008	-1.50677	-0.24316
C	3.71622	-1.16068	-0.45187
C	4.14836	0.14778	-0.64646
C	3.17336	1.14475	-0.5767
C	1.84233	0.83925	-0.35914

C	1.41367	-0.49396	-0.21437
H	2.09112	-2.5368	-0.10744
H	5.20639	0.41814	-0.82357
H	1.10635	1.62674	-0.30483
N	0.03255	-0.63865	-0.06749
S	-0.27955	-3.35654	0.40991
H	-0.45591	0.26675	-0.11746
F	4.24449	2.8578	-1.84729
F	4.41634	2.96153	0.34683
F	2.47271	3.41752	-0.63081
F	4.22292	-3.48548	-0.41832
F	5.67527	-2.10728	-1.41441
F	5.51392	-2.13718	0.7796
C	3.56679	2.5741	-0.70123
C	4.74243	-2.22735	-0.38277
C	-1.78554	2.81473	-0.54136
C	-1.67297	4.99166	-1.30374
C	-3.12272	4.58971	-1.13418
O	-1.44074	1.68526	-0.14617
C	-0.89539	3.96007	-0.96004
O	-4.14147	5.24207	-1.34243
N	-3.08152	3.25671	-0.66813
H	-3.91334	2.68399	-0.46588
H	0.18102	3.88999	-0.95338
H	-4.60595	-0.50532	-1.02224
C	-5.29097	0.96473	0.25278
C	-5.85774	1.82869	-0.61779
C	-5.84314	1.65591	-2.12622
C	-6.5708	3.07679	-0.12545
H	-5.35887	1.15889	1.31799
H	-6.87107	1.64291	-2.51201

H	-5.32918	2.50014	-2.60654
H	-5.35126	0.73389	-2.45111
H	-6.07247	3.97766	-0.51082
H	-7.60914	3.09338	-0.48326
H	-6.58244	3.12609	0.96879
H	-1.38347	5.96979	-1.64941
O	8.72994	0.10329	0.27787
H	8.11025	0.20888	1.07578
H	8.66892	-0.875	-0.01166
O	6.73951	1.39173	-0.74472
H	6.46577	1.44511	0.21884
H	7.63344	0.87546	-0.6
O	6.58666	0.39481	1.81733
H	5.95717	0.97089	2.40129
H	6.10097	-0.42646	1.55012
O	4.82394	1.90684	2.92025
H	4.04274	1.41729	3.28696
H	4.52166	2.35458	2.08299
O	8.38672	-2.37232	-0.6177
H	8.92869	-2.47593	-1.44356
H	7.4378	-2.33142	-0.91511

Figure 3.

TS(Gas) +5H_2O

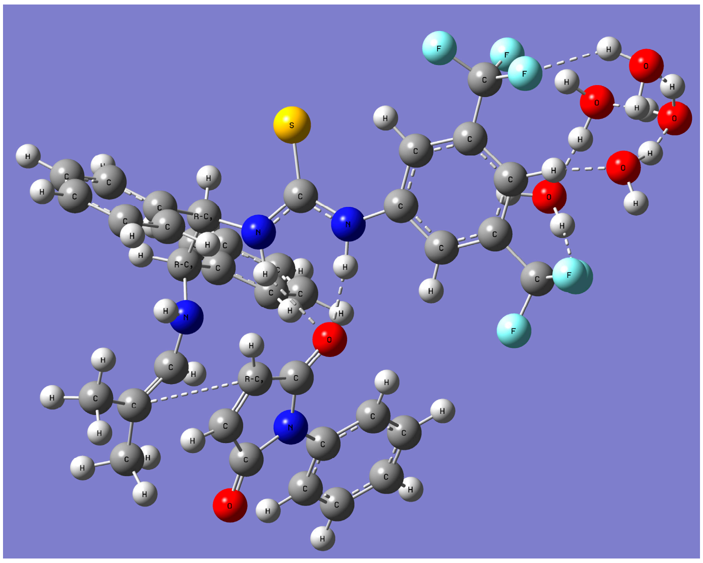

Calculation Type = FREQ

Calculation Method = RB3LYP

Basis Set = 6-31G(d,p)

Charge = 0

Spin = Singlet

Solvation = None

E(RB3LYP) = -3160.9475 Hartree

RMS Gradient Norm = 1.42e-07 Hartree/Bohr

Temperature = 298.15 Kelvin

Pressure = 1 atm

Frequencies scaled by = 1

Electronic Energy (EE) = -3160.9475 Hartree

Zero-point Energy Correction = 0.75884 Hartree

Thermal Correction to Energy = 0.814779 Hartree

Thermal Correction to Enthalpy = 0.815723 Hartree

Thermal Correction to Free Energy = 0.660379 Hartree

EE + Zero-point Energy = -3160.1886 Hartree

EE + Thermal Energy Correction = -3160.1327 Hartree

EE + Thermal Enthalpy Correction = -3160.1317 Hartree

EE + Thermal Free Energy Correction = -3160.2871 Hartree

E (Thermal) = 511.282 kcal/mol

Heat Capacity (Cv) = 209.304 cal/mol-kelvin

Entropy (S) = 326.949 cal/mol-kelvin

Symbolic Z-matrix:

Charge = 0 Multiplicity = 1

C	2.67135	-0.37866	2.27695
C	3.46932	0.7741	2.32576
C	4.01466	1.16863	3.55649
C	3.76706	0.43327	4.71773
C	2.97651	-0.71749	4.66011
C	2.43376	-1.12065	3.43705
H	2.23258	-0.70878	1.34584
H	4.64036	2.05392	3.60405
H	4.19706	0.75284	5.65985
H	2.78972	-1.29634	5.5571
H	1.82883	-2.01824	3.37907
C	2.97449	3.69365	-0.1753
C	3.74277	4.75668	0.32284
C	4.22791	5.74413	-0.53557
C	3.95166	5.68035	-1.9057
C	3.18164	4.62889	-2.40687
C	2.69178	3.64124	-1.54538
H	3.95657	4.81431	1.38547
H	4.81625	6.5626	-0.13738
H	4.32856	6.44641	-2.573
H	2.95575	4.57715	-3.46564
H	2.07471	2.84179	-1.93635

C	3.73623	1.64744	1.10737
C	2.52633	2.59581	0.79248
N	1.4668	1.74626	0.24901
N	4.00052	0.84791	-0.13506
H	2.175	3.04869	1.72236
H	4.59319	2.29492	1.33384
H	1.82661	0.89845	-0.19711
C	0.1343	1.94905	0.36347
C	-2.98852	1.60667	0.00795
C	-4.32	1.25505	-0.21104
C	-4.69608	0.02995	-0.75013
C	-3.66877	-0.86185	-1.06217
C	-2.33406	-0.54837	-0.85315
C	-1.9686	0.70173	-0.31486
H	-2.74146	2.57397	0.414
H	-5.74605	-0.23964	-0.95571
H	-1.5656	-1.26447	-1.11491
N	-0.59247	0.91882	-0.17128
S	-0.50681	3.38302	1.13564
H	-0.01959	0.12482	-0.50294
F	-5.00827	-2.15595	-2.5483
F	-4.52801	-3.04714	-0.5919
F	-2.93743	-2.85411	-2.12265
F	-4.91467	3.50705	0.24341
F	-6.47204	2.16958	-0.63433
F	-5.86997	1.9532	1.48192
C	-4.019	-2.20461	-1.59459
C	-5.37239	2.21924	0.20243
C	2.12938	-1.47314	-1.46164
C	3.68678	-1.28128	-3.14593
C	4.03995	-2.4621	-2.29753

O	1.15575	-1.20941	-0.72425
C	2.57708	-0.71421	-2.66633
O	4.98727	-3.23266	-2.42183
N	3.01586	-2.53797	-1.28167
H	2.04071	0.14572	-3.02994
H	4.07898	1.47879	-0.93913
C	5.05025	-0.10902	-0.07585
C	6.07951	-0.24033	-0.93432
C	6.38763	0.71708	-2.07009
C	7.04461	-1.4	-0.79066
H	4.94762	-0.80391	0.75057
H	7.39575	1.13153	-1.93467
H	6.38028	0.19772	-3.03634
H	5.68601	1.55415	-2.12948
H	6.90336	-2.11293	-1.61476
H	8.08295	-1.04648	-0.8205
H	6.88369	-1.93856	0.14951
H	4.28026	-0.99785	-3.99798
O	-8.51282	-1.27177	0.74586
H	-7.80545	-1.02147	1.49375
H	-8.9445	-0.39523	0.4263
O	-7.35031	-0.95339	-1.38066
H	-7.17521	-1.65982	-2.05026
H	-7.78112	-1.33992	-0.43332
O	-6.69572	-0.62583	2.40492
H	-5.89955	-1.28357	2.30299
H	-6.37643	0.27083	2.12687
O	-4.70943	-2.23599	2.06595
H	-3.83727	-1.84166	2.32264
H	-4.62985	-2.53607	1.12016
O	-9.03486	0.83283	-0.73073

H	-8.4381	0.2015	-1.2972
H	-8.38917	1.52306	-0.41957
C	2.93846	-3.53536	-0.25208
C	1.72242	-3.78625	0.40676
C	4.07623	-4.27906	0.10316
C	1.65584	-4.75644	1.40703
H	0.84636	-3.21365	0.15208
C	3.99335	-5.24841	1.10283
H	5.00522	-4.11738	-0.41731
C	2.78786	-5.49248	1.76309
H	0.70976	-4.93605	1.90397
H	4.88099	-5.81299	1.36274
H	2.73047	-6.24493	2.54016

TS(Toluene) +5H_2O

Calculation Type = FREQ

Calculation Method = RB3LYP

Basis Set = 6-31G(d,p)

Charge = 0

Spin = Singlet

Solvation = scrf=solvent=toluene

E(RB3LYP) = -3160.9659 Hartree

RMS Gradient Norm = 3.53e-07 Hartree/Bohr

Temperature = 298.15 Kelvin

Pressure = 1 atm

Frequencies scaled by = 1

Electronic Energy (EE) = -3160.9659 Hartree

Zero-point Energy Correction = 0.759077 Hartree

Thermal Correction to Energy = 0.814822 Hartree

Thermal Correction to Enthalpy = 0.815766 Hartree

Thermal Correction to Free Energy = 0.662255 Hartree

EE + Zero-point Energy = -3160.2069 Hartree

EE + Thermal Energy Correction = -3160.1511 Hartree

EE + Thermal Enthalpy Correction = -3160.1502 Hartree

EE + Thermal Free Energy Correction = -3160.3037 Hartree

E (Thermal) = 511.308 kcal/mol

Heat Capacity (Cv) = 209.089 cal/mol-kelvin

Entropy (S) = 323.092 cal/mol-kelvin

Symbolic Z-matrix:

Charge = 0 Multiplicity = 1

C	2.75062	-0.45199	2.23246
C	3.49809	0.72989	2.33721
C	4.00745	1.09906	3.59048
C	3.77365	0.31151	4.71877

C	3.03523	-0.86833	4.60472
C	2.52903	-1.24707	3.35929
H	2.33948	-0.76473	1.28285
H	4.59563	2.00657	3.68166
H	4.17419	0.61338	5.67947
H	2.86027	-1.48829	5.47617
H	1.96561	-2.1673	3.25752
C	2.99138	3.69755	-0.10376
C	3.75229	4.74873	0.42799
C	4.25444	5.75193	-0.40069
C	4.00235	5.71732	-1.77579
C	3.23636	4.68064	-2.31092
C	2.73071	3.67719	-1.47834
H	3.94097	4.78794	1.49626
H	4.8341	6.56279	0.02479
H	4.38989	6.49771	-2.41991
H	3.02073	4.65787	-3.37302
H	2.10572	2.89465	-1.88999
C	3.75566	1.65485	1.15646
C	2.53067	2.57604	0.83118
N	1.50181	1.71933	0.24405
N	4.08011	0.9167	-0.11053
H	2.13899	3.00615	1.75633
H	4.58694	2.3202	1.42472
H	1.87846	0.87145	-0.1973
C	0.16063	1.90524	0.37088
C	-2.94169	1.5997	-0.10294
C	-4.27507	1.2446	-0.30251
C	-4.66453	-0.02818	-0.70804
C	-3.64578	-0.95959	-0.91501
C	-2.30814	-0.64314	-0.72269

C	-1.93102	0.64963	-0.31025
H	-2.68389	2.60092	0.20317
H	-5.71881	-0.30879	-0.89995
H	-1.54677	-1.39136	-0.90434
N	-0.55563	0.86997	-0.17726
S	-0.49275	3.30792	1.15791
H	0.01584	0.07575	-0.49175
F	-4.97945	-2.39943	-2.27112
F	-4.53859	-3.06583	-0.21545
F	-2.92478	-3.06581	-1.7334
F	-4.83616	3.54397	-0.13793
F	-6.40114	2.13424	-0.87675
F	-5.84158	2.16915	1.26254
C	-4.00873	-2.34756	-1.30312
C	-5.31777	2.27005	-0.03098
C	2.19113	-1.50967	-1.45341
C	3.82789	-1.23462	-3.04691
C	4.11689	-2.47951	-2.2695
O	1.1969	-1.27679	-0.74662
C	2.7084	-0.67944	-2.57906
O	5.06437	-3.26114	-2.4211
N	3.03708	-2.60041	-1.30681
H	2.20184	0.21318	-2.90495
H	4.15726	1.58786	-0.88165
C	5.1537	-0.00641	-0.06999
C	6.20031	-0.06126	-0.91519
C	6.49822	0.96454	-1.99245
C	7.20584	-1.18776	-0.80914
H	5.06124	-0.73845	0.72472
H	7.481	1.41684	-1.80291
H	6.54847	0.4933	-2.98196

H	5.76175	1.77275	-2.03391
H	7.1138	-1.85717	-1.67543
H	8.2297	-0.79333	-0.79268
H	7.04471	-1.78266	0.09601
H	4.46792	-0.9092	-3.84868
O	-8.75774	-1.01476	0.66872
H	-8.12943	-0.68078	1.45037
H	-9.13684	-0.18321	0.19355
O	-7.29773	-1.00876	-1.2722
H	-7.17896	-1.77656	-1.8844
H	-7.91129	-1.25287	-0.34714
O	-7.04314	-0.18818	2.35096
H	-6.24763	-0.84546	2.34414
H	-6.69539	0.68065	2.0243
O	-4.97307	-1.73539	2.19348
H	-4.12201	-1.26046	2.36808
H	-4.86969	-2.2286	1.33629
O	-8.99831	0.88706	-1.09287
H	-8.33835	0.18632	-1.48075
H	-8.39764	1.62386	-0.80066
C	2.85973	-3.65845	-0.34672
C	1.61939	-3.813	0.29243
C	3.9003	-4.55415	-0.04149
C	1.43038	-4.83477	1.22123
H	0.81718	-3.12659	0.07441
C	3.695	-5.57221	0.88941
H	4.84475	-4.4698	-0.54722
C	2.46398	-5.71985	1.52883
H	0.46408	-4.93684	1.70113
H	4.50862	-6.25321	1.1098
H	2.31138	-6.51231	2.2513

TS(Diethyl ether) +5H_2O

Calculation Type = FREQ

Calculation Method = RB3LYP

Basis Set = 6-31G(d,p)

Charge = 0

Spin = Singlet

Solvation = scrf=solvent=diethylether

E(RB3LYP) = -3160.9752 Hartree

RMS Gradient Norm = 1.034e-06 Hartree/Bohr

Temperature = 298.15 Kelvin

Pressure = 1 atm

Frequencies scaled by = 1

Electronic Energy (EE) = -3160.9752 Hartree

Zero-point Energy Correction = 0.758779 Hartree

Thermal Correction to Energy = 0.814619 Hartree

Thermal Correction to Enthalpy = 0.815564 Hartree

Thermal Correction to Free Energy = 0.661501 Hartree

EE + Zero-point Energy = -3160.2164 Hartree

EE + Thermal Energy Correction = -3160.1606 Hartree

EE + Thermal Enthalpy Correction = -3160.1597 Hartree

EE + Thermal Free Energy Correction = -3160.3137 Hartree

E (Thermal) = 511.181 kcal/mol

Heat Capacity (Cv) = 209.223 cal/mol-kelvin

Entropy (S) = 324.253 cal/mol-kelvin

Symbolic Z-matrix:

Charge = 0 Multiplicity = 1

C	2.55028	-0.28891	2.33655
C	3.3537	0.85963	2.375
C	3.86078	1.28859	3.61008
C	3.57033	0.59166	4.7844

C	2.77585	-0.55636	4.73613
C	2.27092	-0.99414	3.50945
H	2.13933	-0.64465	1.40316
H	4.4917	2.17063	3.65103
H	3.97058	0.93841	5.72999
H	2.55681	-1.10644	5.6438
H	1.66397	-1.89074	3.45835
C	2.98659	3.687	-0.26185
C	3.80593	4.73315	0.18728
C	4.32923	5.66128	-0.71304
C	4.03994	5.55523	-2.07754
C	3.21651	4.52295	-2.53035
C	2.68948	3.5947	-1.62628
H	4.02591	4.82708	1.24606
H	4.95531	6.46875	-0.35171
H	4.44475	6.27676	-2.77729
H	2.97445	4.4441	-3.58398
H	2.0234	2.81517	-1.97622
C	3.67504	1.68807	1.13964
C	2.49562	2.64905	0.7508
N	1.43019	1.79774	0.22627
N	3.97168	0.83766	-0.06279
H	2.13431	3.15661	1.64823
H	4.53804	2.32558	1.37288
H	1.78133	0.93405	-0.17616
C	0.09598	2.00381	0.34065
C	-3.02495	1.58891	0.03257
C	-4.35047	1.22066	-0.19481
C	-4.71018	-0.00736	-0.73877
C	-3.66979	-0.88353	-1.05136
C	-2.33946	-0.55209	-0.84016

C	-1.99081	0.70006	-0.29469
H	-2.79054	2.55949	0.43938
H	-5.75764	-0.29147	-0.95229
H	-1.56121	-1.25592	-1.10691
N	-0.61815	0.9309	-0.1403
S	-0.55048	3.46355	1.03687
H	-0.03409	0.12704	-0.43481
F	-4.9717	-2.18751	-2.56443
F	-4.5222	-3.07304	-0.5978
F	-2.90394	-2.86913	-2.09905
F	-4.98908	3.46532	0.23656
F	-6.52884	2.07877	-0.59597
F	-5.88555	1.90697	1.51327
C	-4.00218	-2.22676	-1.59352
C	-5.41803	2.1685	0.21977
C	2.1858	-1.419	-1.42362
C	3.78467	-1.10504	-3.05311
C	4.15531	-2.30789	-2.24659
O	1.16366	-1.19103	-0.72016
C	2.64312	-0.59724	-2.58305
O	5.12053	-3.03139	-2.38242
N	3.11148	-2.46799	-1.26117
H	2.08596	0.25733	-2.92782
H	4.08882	1.43848	-0.88469
C	5.00422	-0.13194	0.07415
C	6.06944	-0.31609	-0.73111
C	6.45282	0.59242	-1.88369
C	7.0046	-1.48534	-0.49232
H	4.85597	-0.7911	0.92273
H	7.45149	1.01144	-1.70086
H	6.50752	0.03184	-2.82563

H	5.7607	1.42803	-2.02286
H	6.89076	-2.23232	-1.29105
H	8.04968	-1.15129	-0.48781
H	6.79017	-1.97869	0.46145
H	4.39214	-0.76822	-3.87524
O	-8.60079	-1.35811	0.66694
H	-7.93256	-1.08644	1.44112
H	-9.05709	-0.49699	0.33674
O	-7.33014	-1.0056	-1.38014
H	-7.18826	-1.69058	-2.07955
H	-7.82746	-1.40613	-0.45365
O	-6.83622	-0.65371	2.35543
H	-6.03202	-1.2985	2.27793
H	-6.51637	0.24904	2.09994
O	-4.79226	-2.20355	2.03249
H	-3.9305	-1.79257	2.29579
H	-4.69844	-2.52421	1.09519
O	-9.08873	0.74686	-0.80048
H	-8.44002	0.14023	-1.33647
H	-8.48986	1.4607	-0.45212
C	3.07084	-3.52854	-0.28856
C	1.88588	-3.85699	0.397
C	4.23651	-4.26351	-0.02364
C	1.88294	-4.8926	1.33225
H	0.98743	-3.29682	0.21293
C	4.21697	-5.29681	0.9117
H	5.14086	-4.04366	-0.56806
C	3.04542	-5.61786	1.59805
H	0.96115	-5.13205	1.84951
H	5.12827	-5.85204	1.09954
H	3.03724	-6.4211	2.32469

TS(THF) +5H_2O

Calculation Type = FREQ

Calculation Method = RB3LYP

Basis Set = 6-31G(d,p)

Charge = 0

Spin = Singlet

Solvation = scrf=solvent=thf

E(RB3LYP) = -3160.9817 Hartree

RMS Gradient Norm = 1.18e-06 Hartree/Bohr

Temperature = 298.15 Kelvin

Pressure = 1 atm

Frequencies scaled by = 1

Electronic Energy (EE) = -3160.9817 Hartree

Zero-point Energy Correction = 0.758585 Hartree

Thermal Correction to Energy = 0.814441 Hartree

Thermal Correction to Enthalpy = 0.815385 Hartree

Thermal Correction to Free Energy = 0.661171 Hartree

EE + Zero-point Energy = -3160.2231 Hartree

EE + Thermal Energy Correction = -3160.1672 Hartree

EE + Thermal Enthalpy Correction = -3160.1663 Hartree

EE + Thermal Free Energy Correction = -3160.3205 Hartree

E (Thermal) = 511.069 kcal/mol

Heat Capacity (Cv) = 209.254 cal/mol-kelvin

Entropy (S) = 324.57 cal/mol-kelvin

Symbolic Z-matrix:

Charge = 0 Multiplicity = 1

C	2.65968	-0.38054	2.27692
C	3.45798	0.77176	2.33033
C	3.99762	1.1651	3.56394
C	3.74416	0.42899	4.72342

C	2.95349	-0.72143	4.66116
C	2.41637	-1.12338	3.43526
H	2.22513	-0.7098	1.34355
H	4.6236	2.04998	3.61516
H	4.16981	0.74767	5.66782
H	2.76231	-1.30094	5.55678
H	1.81147	-2.02075	3.37366
C	2.98046	3.69132	-0.17466
C	3.75093	4.75188	0.32534
C	4.24229	5.73714	-0.532
C	3.97008	5.6737	-1.90295
C	3.19777	4.62484	-2.40599
C	2.70174	3.63937	-1.54554
H	3.96142	4.80939	1.38863
H	4.83221	6.55375	-0.13236
H	4.35173	6.43813	-2.56942
H	2.97473	4.57361	-3.46539
H	2.08259	2.84223	-1.93794
C	3.73201	1.64533	1.11373
C	2.52542	2.59558	0.79232
N	1.46643	1.74779	0.24516
N	4.00237	0.84613	-0.12773
H	2.17068	3.05031	1.72001
H	4.58869	2.29152	1.3449
H	1.82567	0.89853	-0.19841
C	0.13375	1.95224	0.35672
C	-2.98873	1.61032	-0.00151
C	-4.32024	1.25771	-0.21865
C	-4.69646	0.03091	-0.7539
C	-3.66907	-0.86124	-1.06463
C	-2.33426	-0.54675	-0.85742

C	-1.96874	0.70468	-0.32234
H	-2.74162	2.57877	0.40182
H	-5.74667	-0.23994	-0.95726
H	-1.56575	-1.26325	-1.118
N	-0.59267	0.92215	-0.17921
S	-0.50766	3.387	1.12622
H	-0.01959	0.12804	-0.51005
F	-5.01143	-2.16223	-2.54231
F	-4.52202	-3.04671	-0.58486
F	-2.93769	-2.85501	-2.12211
F	-4.91533	3.51093	0.22917
F	-6.47338	2.16986	-0.6419
F	-5.86812	1.96064	1.47413
C	-4.01877	-2.20606	-1.59215
C	-5.37252	2.22288	0.19298
C	2.13565	-1.46794	-1.46245
C	3.70458	-1.26917	-3.13518
C	4.05012	-2.4553	-2.29137
O	1.15708	-1.20644	-0.7306
C	2.59266	-0.70297	-2.65962
O	4.99661	-3.2271	-2.41461
N	3.01925	-2.53511	-1.28232
H	2.06005	0.15983	-3.02196
H	4.08622	1.47761	-0.93081
C	5.04996	-0.11252	-0.06331
C	6.08466	-0.24399	-0.91535
C	6.40264	0.71593	-2.04627
C	7.04671	-1.40556	-0.76746
H	4.94096	-0.8086	0.76127
H	7.40979	1.12953	-1.90131
H	6.40329	0.199	-3.01383

H	5.70201	1.55357	-2.10932
H	6.90917	-2.11693	-1.59356
H	8.08584	-1.05392	-0.7903
H	6.87888	-1.9453	0.1708
H	4.30405	-0.98213	-3.98179
O	-8.51131	-1.2693	0.75221
H	-7.8028	-1.01668	1.49815
H	-8.94452	-0.39389	0.43148
O	-7.35081	-0.95519	-1.37566
H	-7.1787	-1.66283	-2.04474
H	-7.78152	-1.33976	-0.42688
O	-6.69062	-0.61651	2.40434
H	-5.89299	-1.27246	2.30412
H	-6.37364	0.28023	2.12406
O	-4.69868	-2.22098	2.06911
H	-3.82757	-1.8224	2.32266
H	-4.6195	-2.52648	1.12505
O	-9.03614	0.83146	-0.72792
H	-8.43947	0.19942	-1.29369
H	-8.39111	1.52375	-0.42
C	2.93355	-3.53836	-0.25891
C	1.71444	-3.78673	0.39526
C	4.06623	-4.29057	0.09475
C	1.63989	-4.76283	1.38917
H	0.84225	-3.20779	0.14181
C	3.97533	-5.26569	1.08806
H	4.99743	-4.13083	-0.42228
C	2.76682	-5.50734	1.7436
H	0.6915	-4.94036	1.88247
H	4.85919	-5.83677	1.34668
H	2.70323	-6.26435	2.51573

TS(Cabon tetrachloride) +5H_2O

Calculation Type = FREQ

Calculation Method = RB3LYP

Basis Set = 6-31G(d,p)

Charge = 0

Spin = Singlet

Solvation = scrf=solvent=ccl4

E(RB3LYP) = -3160.9648 Hartree

RMS Gradient Norm = 3.97e-07 Hartree/Bohr

Temperature = 298.15 Kelvin

Pressure = 1 atm

Frequencies scaled by = 1

Electronic Energy (EE) = -3160.9648 Hartree

Zero-point Energy Correction = 0.759043 Hartree

Thermal Correction to Energy = 0.814809 Hartree

Thermal Correction to Enthalpy = 0.815753 Hartree

Thermal Correction to Free Energy = 0.66209 Hartree

EE + Zero-point Energy = -3160.2057 Hartree

EE + Thermal Energy Correction = -3160.15 Hartree

EE + Thermal Enthalpy Correction = -3160.149 Hartree

EE + Thermal Free Energy Correction = -3160.3027 Hartree

E (Thermal) = 511.3 kcal/mol

Heat Capacity (Cv) = 209.11 cal/mol-kelvin

Entropy (S) = 323.411 cal/mol-kelvin

Symbolic Z-matrix:

Charge = 0 Multiplicity = 1

C	2.62899	-0.40703	2.27324
C	3.39931	0.76144	2.357
C	3.9051	1.14994	3.60581
C	3.64545	0.39404	4.74997

C	2.88526	-0.77381	4.65644
C	2.38259	-1.17172	3.41565
H	2.21884	-0.73266	1.328
H	4.51082	2.04732	3.68116
H	4.0432	0.71061	5.70709
H	2.69091	-1.36992	5.54031
H	1.80434	-2.08418	3.32892
C	2.96528	3.67871	-0.16294
C	3.75152	4.72695	0.33669
C	4.27114	5.69599	-0.52136
C	4.01135	5.62959	-1.89392
C	3.22002	4.59594	-2.39715
C	2.69696	3.62682	-1.53516
H	3.9462	4.79127	1.40267
H	4.87039	6.50529	-0.12079
H	4.4125	6.38356	-2.56077
H	2.99808	4.54942	-3.45717
H	2.05211	2.84725	-1.92137
C	3.6876	1.6517	1.15751
C	2.48421	2.59228	0.80288
N	1.43896	1.74137	0.23913
N	4.01321	0.87241	-0.08566
H	2.10358	3.05602	1.71636
H	4.53086	2.30529	1.41729
H	1.79638	0.88124	-0.17904
C	0.09994	1.9449	0.35211
C	-3.01069	1.5934	-0.05488
C	-4.34014	1.23171	-0.27018
C	-4.71453	-0.01644	-0.75751
C	-3.68377	-0.91817	-1.0276
C	-2.3497	-0.59575	-0.8211

C	-1.9871	0.67466	-0.3306
H	-2.76446	2.57741	0.31146
H	-5.76641	-0.30202	-0.95871
H	-1.57916	-1.3203	-1.05323
N	-0.61428	0.90031	-0.18676
S	-0.54896	3.36672	1.10665
H	-0.03472	0.10907	-0.50515
F	-5.01078	-2.29108	-2.45734
F	-4.52918	-3.08194	-0.45608
F	-2.93641	-2.95199	-1.992
F	-4.94154	3.50602	0.03698
F	-6.49996	2.10587	-0.73534
F	-5.87925	2.03539	1.3857
C	-4.02772	-2.28464	-1.50149
C	-5.39513	2.21844	0.08522
C	2.20269	-1.42773	-1.46139
C	3.84584	-1.1267	-3.04567
C	4.16853	-2.3485	-2.24775
O	1.18026	-1.21032	-0.7739
C	2.70398	-0.59924	-2.59588
O	5.12659	-3.10486	-2.37732
N	3.09323	-2.489	-1.28897
H	2.17251	0.27091	-2.94255
H	4.10984	1.51858	-0.87526
C	5.06438	-0.07443	-0.00375
C	6.12416	-0.18668	-0.83006
C	6.47444	0.80191	-1.92658
C	7.09575	-1.33612	-0.6636
H	4.94287	-0.77801	0.81262
H	7.45267	1.25226	-1.71027
H	6.56123	0.30075	-2.89863

H	5.74794	1.61419	-2.02367
H	6.98559	-2.04755	-1.49416
H	8.13069	-0.97208	-0.66025
H	6.91263	-1.87996	0.26919
H	4.47961	-0.79478	-3.84965
O	-8.5656	-1.25223	0.76528
H	-7.89179	-0.96177	1.525
H	-9.026	-0.40344	0.40779
O	-7.33277	-1.02204	-1.3139
H	-7.22638	-1.74373	-1.98196
H	-7.82535	-1.36514	-0.34815
O	-6.77828	-0.48288	2.40137
H	-5.94884	-1.09168	2.33429
H	-6.4962	0.42412	2.11888
O	-4.63673	-1.91021	2.06908
H	-3.81641	-1.35657	2.11674
H	-4.62983	-2.36882	1.18601
O	-9.07444	0.7693	-0.79596
H	-8.44231	0.13074	-1.3136
H	-8.46825	1.50703	-0.51736
C	2.97794	-3.544	-0.31794
C	1.75623	-3.78488	0.33386
C	4.08471	-4.35484	-0.01684
C	1.6544	-4.81086	1.27279
H	0.90435	-3.16294	0.1186
C	3.96613	-5.37753	0.92326
H	5.0156	-4.20172	-0.53589
C	2.7557	-5.61235	1.57605
H	0.7033	-4.98321	1.76301
H	4.83081	-5.99334	1.14073
H	2.67083	-6.40829	2.3058

TS(Dichloromethane) +5H_2O

Calculation Type = FREQ

Calculation Method = RB3LYP

Basis Set = 6-31G(d,p)

Charge = 0

Spin = Singlet

Solvation = scrf=solvent=dichloromethane

E(RB3LYP) = -3160.9699 Hartree

RMS Gradient Norm = 1.382e-06 Hartree/Bohr

Temperature = 298.15 Kelvin

Pressure = 1 atm

Frequencies scaled by = 1

Electronic Energy (EE) = -3160.9699 Hartree

Zero-point Energy Correction = 0.758 Hartree

Thermal Correction to Energy = 0.814663 Hartree

Thermal Correction to Enthalpy = 0.815607 Hartree

Thermal Correction to Free Energy = 0.659533 Hartree

EE + Zero-point Energy = -3160.2119 Hartree

EE + Thermal Energy Correction = -3160.1552 Hartree

EE + Thermal Enthalpy Correction = -3160.1543 Hartree

EE + Thermal Free Energy Correction = -3160.3104 Hartree

E (Thermal) = 511.209 kcal/mol

Heat Capacity (Cv) = 211.428 cal/mol-kelvin

Entropy (S) = 328.485 cal/mol-kelvin

Symbolic Z-matrix:

Charge = 0 Multiplicity = 1

C	-3.12235	0.21549	2.56868
C	-4.02673	-0.82673	2.31271
C	-4.60018	-1.50913	3.39487
C	-4.27491	-1.1673	4.70921

C	-3.37397	-0.12898	4.95721
C	-2.8028	0.56073	3.88409
H	-2.67236	0.76188	1.75032
H	-5.30744	-2.31035	3.20642
H	-4.7276	-1.70505	5.53406
H	-3.12259	0.14308	5.97568
H	-2.10593	1.36986	4.07098
C	-3.77053	-2.92878	-0.93502
C	-4.66991	-3.99435	-0.78125
C	-5.2214	-4.62367	-1.89774
C	-4.8812	-4.19385	-3.18485
C	-3.98129	-3.13863	-3.34514
C	-3.42556	-2.51035	-2.22565
H	-4.93211	-4.33763	0.21454
H	-5.91026	-5.44985	-1.76526
H	-5.30858	-4.6826	-4.05242
H	-3.70443	-2.80716	-4.33929
H	-2.7066	-1.71061	-2.35497
C	-4.37044	-1.28988	0.90291
C	-3.24445	-2.22152	0.31593
N	-2.09645	-1.35794	0.04527
N	-4.51826	-0.16641	-0.05544
H	-2.96162	-2.96288	1.06669
H	-5.28458	-1.897	0.95582
H	-2.35857	-0.38301	-0.14777
C	-0.79194	-1.71608	0.10493
C	2.3812	-1.5075	-0.24879
C	3.71768	-1.16206	-0.45638
C	4.1499	0.14589	-0.65379
C	3.17478	1.14306	-0.58795
C	1.84354	0.83809	-0.37144

C	1.41474	-0.49477	-0.22375
H	2.09231	-2.53722	-0.11088
H	5.2079	0.41528	-0.82924
H	1.10767	1.62582	-0.31957
N	0.03343	-0.63892	-0.07701
S	-0.27942	-3.35754	0.39772
H	-0.45475	0.26686	-0.12749
F	4.24934	2.85176	-1.86085
F	4.41647	2.962	0.33312
F	2.47503	3.41596	-0.65008
F	4.22439	-3.48673	-0.41781
F	5.68166	-2.10926	-1.40802
F	5.50906	-2.13704	0.78522
C	3.56842	2.57205	-0.71524
C	4.74367	-2.22844	-0.38107
C	-2.69432	3.05603	-0.80583
C	-3.57889	4.74432	-2.10961
C	-3.70529	5.11578	-0.64742
O	-2.14563	1.98901	-0.47246
C	-2.99208	3.54684	-2.20216
O	-4.18438	6.1251	-0.13874
N	-3.14029	4.02955	0.05704
H	-2.74076	2.97699	-3.08289
H	-4.60743	-0.50018	-1.01726
C	-5.29007	0.96571	0.26363
C	-5.86311	1.82904	-0.60342
C	-5.8576	1.65587	-2.11183
C	-6.57166	3.07796	-0.10665
H	-5.35158	1.15968	1.32926
H	-6.88742	1.65656	-2.49266
H	-5.33435	2.4926	-2.5953

H	-5.37995	0.72699	-2.43839
H	-6.07319	3.97834	-0.49305
H	-7.61151	3.09694	-0.45989
H	-6.57817	3.12617	0.98768
H	-3.92609	5.389	-2.89947
O	8.72487	0.10311	0.29816
H	8.09545	0.21061	1.08855
H	8.66536	-0.87553	0.00876
O	6.74511	1.39203	-0.74666
H	6.46086	1.44625	0.21388
H	7.63602	0.8738	-0.59196
O	6.56881	0.4029	1.81589
H	5.9401	0.98249	2.39804
H	6.08277	-0.41947	1.55287
O	4.8144	1.92497	2.91552
H	4.03247	1.4398	3.28656
H	4.51147	2.36795	2.07597
O	8.38874	-2.37376	-0.59497
H	8.93516	-2.47994	-1.4176
H	7.44151	-2.33431	-0.89787
C	-3.05997	3.93904	1.52205
C	-1.93905	4.4316	2.19092
C	-4.10438	3.36085	2.24347
C	-1.86234	4.34534	3.58085
H	-1.11548	4.88668	1.62182
C	-4.02824	3.27537	3.63391
H	-4.98799	2.97274	1.71647
C	-2.90741	3.76736	4.30265
H	-0.97857	4.73299	4.10808
H	-4.85205	2.81981	4.20249
H	-2.84673	3.69951	5.39855

TS(DMSO) +5H_2O

Calculation Type = FREQ

Calculation Method = RB3LYP

Basis Set = 6-31G(d,p)

Charge = 0

Spin = Singlet

Solvation = scrf=solvent=dmso

E(RB3LYP) = -3160.991 Hartree

RMS Gradient Norm = 8.9e-07 Hartree/Bohr

Temperature = 298.15 Kelvin

Pressure = 1 atm

Frequencies scaled by = 1

Electronic Energy (EE) = -3160.991 Hartree

Zero-point Energy Correction = 0.757961 Hartree

Thermal Correction to Energy = 0.814018 Hartree

Thermal Correction to Enthalpy = 0.814962 Hartree

Thermal Correction to Free Energy = 0.659777 Hartree

EE + Zero-point Energy = -3160.233 Hartree

EE + Thermal Energy Correction = -3160.177 Hartree

EE + Thermal Enthalpy Correction = -3160.176 Hartree

EE + Thermal Free Energy Correction = -3160.3312 Hartree

E (Thermal) = 510.804 kcal/mol

Heat Capacity (Cv) = 209.505 cal/mol-kelvin

Entropy (S) = 326.615 cal/mol-kelvin

Symbolic Z-matrix:

Charge = 0 Multiplicity = 1

C	2.67135	-0.37866	2.27695
C	3.46932	0.7741	2.32576
C	4.01466	1.16863	3.55649
C	3.76706	0.43327	4.71773

C	2.97651	-0.7175	4.66011
C	2.43377	-1.12065	3.43705
H	2.23258	-0.70878	1.34585
H	4.64036	2.05392	3.60405
H	4.19706	0.75284	5.65986
H	2.78972	-1.29634	5.5571
H	1.82883	-2.01824	3.37907
C	2.97449	3.69365	-0.1753
C	3.74276	4.75669	0.32285
C	4.2279	5.74414	-0.53556
C	3.95165	5.68036	-1.90569
C	3.18164	4.62889	-2.40687
C	2.69178	3.64124	-1.54537
H	3.95655	4.81432	1.38548
H	4.81623	6.56261	-0.13737
H	4.32855	6.44642	-2.57299
H	2.95575	4.57715	-3.46564
H	2.07471	2.84178	-1.93635
C	3.73623	1.64744	1.10737
C	2.52633	2.59581	0.79248
N	1.4668	1.74626	0.24902
N	4.00052	0.84791	-0.13505
H	2.175	3.04869	1.72237
H	4.59319	2.29492	1.33384
H	1.82661	0.89845	-0.1971
C	0.1343	1.94905	0.36347
C	-2.98853	1.60667	0.00794
C	-4.32	1.25505	-0.21106
C	-4.69608	0.02994	-0.75014
C	-3.66877	-0.86186	-1.06217
C	-2.33406	-0.54838	-0.85315

C	-1.9686	0.70173	-0.31487
H	-2.74146	2.57398	0.41398
H	-5.74605	-0.23965	-0.95571
H	-1.5656	-1.26448	-1.1149
N	-0.59247	0.91882	-0.17129
S	-0.50681	3.38302	1.13564
H	-0.01959	0.12482	-0.50294
F	-5.00827	-2.15597	-2.54829
F	-4.52801	-3.04714	-0.59188
F	-2.93742	-2.85412	-2.12264
F	-4.91467	3.50705	0.24338
F	-6.47205	2.16957	-0.63435
F	-5.86997	1.95321	1.4819
C	-4.019	-2.20462	-1.59457
C	-5.37239	2.21924	0.20241
C	2.12938	-1.47314	-1.46164
C	3.68678	-1.28127	-3.14594
C	4.03995	-2.4621	-2.29754
O	1.15576	-1.20941	-0.72426
C	2.57707	-0.71421	-2.66633
O	4.98727	-3.23265	-2.42184
N	3.01587	-2.53797	-1.28168
H	2.04071	0.14573	-3.02994
H	4.07897	1.4788	-0.93913
C	5.05025	-0.10902	-0.07585
C	6.07951	-0.24033	-0.93432
C	6.38763	0.71708	-2.07009
C	7.04461	-1.39999	-0.79066
H	4.94762	-0.8039	0.75057
H	7.39575	1.13154	-1.93466
H	6.38027	0.19773	-3.03634

H	5.68601	1.55416	-2.12947
H	6.90336	-2.11292	-1.61476
H	8.08295	-1.04647	-0.8205
H	6.88369	-1.93856	0.14951
H	4.28026	-0.99785	-3.99799
O	-8.51281	-1.27177	0.74587
H	-7.80544	-1.02146	1.49376
H	-8.94449	-0.39523	0.42631
O	-7.35031	-0.95341	-1.38066
H	-7.17521	-1.65984	-2.05025
H	-7.78111	-1.33993	-0.43331
O	-6.69571	-0.62582	2.40492
H	-5.89954	-1.28355	2.303
H	-6.37642	0.27084	2.12687
O	-4.70941	-2.23597	2.06596
H	-3.83725	-1.84163	2.32265
H	-4.62983	-2.53607	1.12017
O	-9.03486	0.83282	-0.73073
H	-8.43811	0.20148	-1.2972
H	-8.38917	1.52305	-0.41958
C	2.93847	-3.53535	-0.25209
C	1.72243	-3.78625	0.40675
C	4.07623	-4.27906	0.10315
C	1.65585	-4.75644	1.40702
H	0.84636	-3.21365	0.15208
C	3.99336	-5.24841	1.10282
H	5.00522	-4.11738	-0.41731
C	2.78787	-5.49249	1.76308
H	0.70977	-4.93605	1.90397
H	4.881	-5.81299	1.36273
H	2.73048	-6.24494	2.54015

TS(EtOH) +5H_2O

Calculation Type = FREQ

Calculation Method = RB3LYP

Basis Set = 6-31G(d,p)

Charge = 0

Spin = Singlet

Solvation = scrf=solvent=ethanol

E(RB3LYP) = -3160.9892 Hartree

RMS Gradient Norm = 9.13e-07 Hartree/Bohr

Temperature = 298.15 Kelvin

Pressure = 1 atm

Frequencies scaled by = 1

Electronic Energy (EE) = -3160.9892 Hartree

Zero-point Energy Correction = 0.758137 Hartree

Thermal Correction to Energy = 0.814152 Hartree

Thermal Correction to Enthalpy = 0.815096 Hartree

Thermal Correction to Free Energy = 0.660088 Hartree

EE + Zero-point Energy = -3160.2311 Hartree

EE + Thermal Energy Correction = -3160.175 Hartree

EE + Thermal Enthalpy Correction = -3160.1741 Hartree

EE + Thermal Free Energy Correction = -3160.3291 Hartree

E (Thermal) = 510.888 kcal/mol

Heat Capacity (Cv) = 209.442 cal/mol-kelvin

Entropy (S) = 326.241 cal/mol-kelvin

Symbolic Z-matrix:

Charge = 0 Multiplicity = 1

C	2.65968	-0.38054	2.27692
C	3.45798	0.77176	2.33033
C	3.99762	1.1651	3.56394
C	3.74416	0.42899	4.72342

C	2.95349	-0.72143	4.66116
C	2.41637	-1.12338	3.43526
H	2.22513	-0.7098	1.34355
H	4.6236	2.04998	3.61516
H	4.16981	0.74767	5.66782
H	2.76231	-1.30094	5.55678
H	1.81147	-2.02075	3.37366
C	2.98046	3.69132	-0.17466
C	3.75093	4.75188	0.32534
C	4.24229	5.73714	-0.532
C	3.97008	5.6737	-1.90295
C	3.19777	4.62484	-2.40599
C	2.70174	3.63937	-1.54554
H	3.96142	4.80939	1.38863
H	4.83221	6.55375	-0.13236
H	4.35173	6.43813	-2.56942
H	2.97473	4.57361	-3.46539
H	2.08259	2.84223	-1.93794
C	3.73201	1.64533	1.11373
C	2.52542	2.59558	0.79232
N	1.46643	1.74779	0.24516
N	4.00237	0.84613	-0.12773
H	2.17068	3.05031	1.72001
H	4.58869	2.29152	1.3449
H	1.82567	0.89853	-0.19841
C	0.13375	1.95224	0.35672
C	-2.98873	1.61032	-0.00151
C	-4.32024	1.25771	-0.21865
C	-4.69646	0.03091	-0.7539
C	-3.66907	-0.86124	-1.06463
C	-2.33426	-0.54675	-0.85742

C	-1.96874	0.70468	-0.32234
H	-2.74162	2.57877	0.40182
H	-5.74667	-0.23994	-0.95726
H	-1.56575	-1.26325	-1.118
N	-0.59267	0.92215	-0.17921
S	-0.50766	3.387	1.12622
H	-0.01959	0.12804	-0.51005
F	-5.01143	-2.16223	-2.54231
F	-4.52202	-3.04671	-0.58486
F	-2.93769	-2.85501	-2.12211
F	-4.91533	3.51093	0.22917
F	-6.47338	2.16986	-0.6419
F	-5.86812	1.96064	1.47413
C	-4.01877	-2.20606	-1.59215
C	-5.37252	2.22288	0.19298
C	2.13565	-1.46794	-1.46245
C	3.70458	-1.26917	-3.13518
C	4.05012	-2.4553	-2.29137
O	1.15708	-1.20644	-0.7306
C	2.59266	-0.70297	-2.65962
O	4.99661	-3.2271	-2.41461
N	3.01925	-2.53511	-1.28232
H	2.06005	0.15983	-3.02196
H	4.08622	1.47761	-0.93081
C	5.04996	-0.11252	-0.06331
C	6.08466	-0.24399	-0.91535
C	6.40264	0.71593	-2.04627
C	7.04671	-1.40556	-0.76746
H	4.94096	-0.8086	0.76127
H	7.40979	1.12953	-1.90131
H	6.40329	0.199	-3.01383

H	5.70201	1.55357	-2.10932
H	6.90917	-2.11693	-1.59356
H	8.08584	-1.05392	-0.7903
H	6.87888	-1.9453	0.1708
H	4.30405	-0.98213	-3.98179
O	-8.51131	-1.2693	0.75221
H	-7.8028	-1.01668	1.49815
H	-8.94452	-0.39389	0.43148
O	-7.35081	-0.95519	-1.37566
H	-7.1787	-1.66283	-2.04474
H	-7.78152	-1.33976	-0.42688
O	-6.69062	-0.61651	2.40434
H	-5.89299	-1.27246	2.30412
H	-6.37364	0.28023	2.12406
O	-4.69868	-2.22098	2.06911
H	-3.82757	-1.8224	2.32266
H	-4.6195	-2.52648	1.12505
O	-9.03614	0.83146	-0.72792
H	-8.43947	0.19942	-1.29369
H	-8.39111	1.52375	-0.42
C	2.93355	-3.53836	-0.25891
C	1.71444	-3.78673	0.39526
C	4.06623	-4.29057	0.09475
C	1.63989	-4.76283	1.38917
H	0.84225	-3.20779	0.14181
C	3.97533	-5.26569	1.08806
H	4.99743	-4.13083	-0.42228
C	2.76682	-5.50734	1.7436
H	0.6915	-4.94036	1.88247
H	4.85919	-5.83677	1.34668
H	2.70323	-6.26435	2.5157

TS(Formic acid) +5H_2O

Calculation Type = FREQ

Calculation Method = RB3LYP

Basis Set = 6-31G(d,p)

Charge = 0

Spin = Singlet

Solvation = scrf=solvent=formicacid

E(RB3LYP) = -3160.9911 Hartree

RMS Gradient Norm = 8.77e-07 Hartree/Bohr

Temperature = 298.15 Kelvin

Pressure = 1 atm

Frequencies scaled by = 1

Electronic Energy (EE) = -3160.9911 Hartree

Zero-point Energy Correction = 0.757932 Hartree

Thermal Correction to Energy = 0.813998 Hartree

Thermal Correction to Enthalpy = 0.814942 Hartree

Thermal Correction to Free Energy = 0.659706 Hartree

EE + Zero-point Energy = -3160.2332 Hartree

EE + Thermal Energy Correction = -3160.1772 Hartree

EE + Thermal Enthalpy Correction = -3160.1762 Hartree

EE + Thermal Free Energy Correction = -3160.3314 Hartree

E (Thermal) = 510.791 kcal/mol

Heat Capacity (Cv) = 209.516 cal/mol-kelvin

Entropy (S) = 326.722 cal/mol-kelvin

Symbolic Z-matrix:

Charge = 0 Multiplicity = 1

C	2.67135	-0.37866	2.27695
C	3.46932	0.7741	2.32576
C	4.01466	1.16863	3.55649
C	3.76706	0.43327	4.71773

C	2.97651	-0.7175	4.66011
C	2.43377	-1.12065	3.43705
H	2.23258	-0.70878	1.34585
H	4.64036	2.05392	3.60405
H	4.19706	0.75284	5.65986
H	2.78972	-1.29634	5.5571
H	1.82883	-2.01824	3.37907
C	2.97449	3.69365	-0.1753
C	3.74276	4.75669	0.32285
C	4.2279	5.74414	-0.53556
C	3.95165	5.68036	-1.90569
C	3.18164	4.62889	-2.40687
C	2.69178	3.64124	-1.54537
H	3.95655	4.81432	1.38548
H	4.81623	6.56261	-0.13737
H	4.32855	6.44642	-2.57299
H	2.95575	4.57715	-3.46564
H	2.07471	2.84178	-1.93635
C	3.73623	1.64744	1.10737
C	2.52633	2.59581	0.79248
N	1.4668	1.74626	0.24902
N	4.00052	0.84791	-0.13505
H	2.175	3.04869	1.72237
H	4.59319	2.29492	1.33384
H	1.82661	0.89845	-0.1971
C	0.1343	1.94905	0.36347
C	-2.98853	1.60667	0.00794
C	-4.32	1.25505	-0.21106
C	-4.69608	0.02994	-0.75014
C	-3.66877	-0.86186	-1.06217
C	-2.33406	-0.54838	-0.85315

C	-1.9686	0.70173	-0.31487
H	-2.74146	2.57398	0.41398
H	-5.74605	-0.23965	-0.95571
H	-1.5656	-1.26448	-1.1149
N	-0.59247	0.91882	-0.17129
S	-0.50681	3.38302	1.13564
H	-0.01959	0.12482	-0.50294
F	-5.00827	-2.15597	-2.54829
F	-4.52801	-3.04714	-0.59188
F	-2.93742	-2.85412	-2.12264
F	-4.91467	3.50705	0.24338
F	-6.47205	2.16957	-0.63435
F	-5.86997	1.95321	1.4819
C	-4.019	-2.20462	-1.59457
C	-5.37239	2.21924	0.20241
C	2.12938	-1.47314	-1.46164
C	3.68678	-1.28127	-3.14594
C	4.03995	-2.4621	-2.29754
O	1.15576	-1.20941	-0.72426
C	2.57707	-0.71421	-2.66633
O	4.98727	-3.23265	-2.42184
N	3.01587	-2.53797	-1.28168
H	2.04071	0.14573	-3.02994
H	4.07897	1.4788	-0.93913
C	5.05025	-0.10902	-0.07585
C	6.07951	-0.24033	-0.93432
C	6.38763	0.71708	-2.07009
C	7.04461	-1.39999	-0.79066
H	4.94762	-0.8039	0.75057
H	7.39575	1.13154	-1.93466
H	6.38027	0.19773	-3.03634

H	5.68601	1.55416	-2.12947
H	6.90336	-2.11292	-1.61476
H	8.08295	-1.04647	-0.8205
H	6.88369	-1.93856	0.14951
H	4.28026	-0.99785	-3.99799
O	-8.51281	-1.27177	0.74587
H	-7.80544	-1.02146	1.49376
H	-8.94449	-0.39523	0.42631
O	-7.35031	-0.95341	-1.38066
H	-7.17521	-1.65984	-2.05025
H	-7.78111	-1.33993	-0.43331
O	-6.69571	-0.62582	2.40492
H	-5.89954	-1.28355	2.303
H	-6.37642	0.27084	2.12687
O	-4.70941	-2.23597	2.06596
H	-3.83725	-1.84163	2.32265
H	-4.62983	-2.53607	1.12017
O	-9.03486	0.83282	-0.73073
H	-8.43811	0.20148	-1.2972
H	-8.38917	1.52305	-0.41958
C	2.93847	-3.53535	-0.25209
C	1.72243	-3.78625	0.40675
C	4.07623	-4.27906	0.10315
C	1.65585	-4.75644	1.40702
H	0.84636	-3.21365	0.15208
C	3.99336	-5.24841	1.10282
H	5.00522	-4.11738	-0.41731
C	2.78787	-5.49249	1.76308
H	0.70977	-4.93605	1.90397
H	4.881	-5.81299	1.36273
H	2.73048	-6.24494	2.54015

TS(Water) +5H_2O

Calculation Type = FREQ

Calculation Method = RB3LYP

Basis Set = 6-31G(d,p)

Charge = 0

Spin = Singlet

Solvation = scrf=solvent=water

E(RB3LYP) = -3160.9918 Hartree

RMS Gradient Norm = 8.18e-07 Hartree/Bohr

Temperature = 298.15 Kelvin

Pressure = 1 atm

Frequencies scaled by = 1

Electronic Energy (EE) = -3160.9918 Hartree

Zero-point Energy Correction = 0.757812 Hartree

Thermal Correction to Energy = 0.813916 Hartree

Thermal Correction to Enthalpy = 0.81486 Hartree

Thermal Correction to Free Energy = 0.659441 Hartree

EE + Zero-point Energy = -3160.234 Hartree

EE + Thermal Energy Correction = -3160.1779 Hartree

EE + Thermal Enthalpy Correction = -3160.177 Hartree

EE + Thermal Free Energy Correction = -3160.3324 Hartree

E (Thermal) = 510.74 kcal/mol

Heat Capacity (Cv) = 209.564 cal/mol-kelvin

Entropy (S) = 327.108 cal/mol-kelvin

Symbolic Z-matrix:

Charge = 0 Multiplicity = 1

C	2.67135	-0.37866	2.27695
C	3.46932	0.7741	2.32576
C	4.01466	1.16863	3.55649
C	3.76706	0.43327	4.71773

C	2.97651	-0.71749	4.66011
C	2.43376	-1.12065	3.43705
H	2.23258	-0.70878	1.34584
H	4.64036	2.05392	3.60405
H	4.19706	0.75284	5.65985
H	2.78972	-1.29634	5.5571
H	1.82883	-2.01824	3.37907
C	2.97449	3.69365	-0.1753
C	3.74277	4.75668	0.32284
C	4.22791	5.74413	-0.53557
C	3.95166	5.68035	-1.9057
C	3.18164	4.62889	-2.40687
C	2.69178	3.64124	-1.54538
H	3.95657	4.81431	1.38547
H	4.81625	6.5626	-0.13738
H	4.32856	6.44641	-2.573
H	2.95575	4.57715	-3.46564
H	2.07471	2.84179	-1.93635
C	3.73623	1.64744	1.10737
C	2.52633	2.59581	0.79248
N	1.4668	1.74626	0.24901
N	4.00052	0.84791	-0.13506
H	2.175	3.04869	1.72236
H	4.59319	2.29492	1.33384
H	1.82661	0.89845	-0.19711
C	0.1343	1.94905	0.36347
C	-2.98852	1.60667	0.00795
C	-4.32	1.25505	-0.21104
C	-4.69608	0.02995	-0.75013
C	-3.66877	-0.86185	-1.06217
C	-2.33406	-0.54837	-0.85315

C	-1.9686	0.70173	-0.31486
H	-2.74146	2.57397	0.414
H	-5.74605	-0.23964	-0.95571
H	-1.5656	-1.26447	-1.11491
N	-0.59247	0.91882	-0.17128
S	-0.50681	3.38302	1.13564
H	-0.01959	0.12482	-0.50294
F	-5.00827	-2.15595	-2.5483
F	-4.52801	-3.04714	-0.5919
F	-2.93743	-2.85411	-2.12265
F	-4.91467	3.50705	0.24341
F	-6.47204	2.16958	-0.63433
F	-5.86997	1.9532	1.48192
C	-4.019	-2.20461	-1.59459
C	-5.37239	2.21924	0.20243
C	2.12938	-1.47314	-1.46164
C	3.68678	-1.28128	-3.14593
C	4.03995	-2.4621	-2.29753
O	1.15575	-1.20941	-0.72425
C	2.57708	-0.71421	-2.66633
O	4.98727	-3.23266	-2.42183
N	3.01586	-2.53797	-1.28167
H	2.04071	0.14572	-3.02994
H	4.07898	1.47879	-0.93913
C	5.05025	-0.10902	-0.07585
C	6.07951	-0.24033	-0.93432
C	6.38763	0.71708	-2.07009
C	7.04461	-1.4	-0.79066
H	4.94762	-0.80391	0.75057
H	7.39575	1.13153	-1.93467
H	6.38028	0.19772	-3.03634

H	5.68601	1.55415	-2.12948
H	6.90336	-2.11293	-1.61476
H	8.08295	-1.04648	-0.8205
H	6.88369	-1.93856	0.14951
H	4.28026	-0.99785	-3.99798
O	-8.51282	-1.27177	0.74586
H	-7.80545	-1.02147	1.49375
H	-8.9445	-0.39523	0.4263
O	-7.35031	-0.95339	-1.38066
H	-7.17521	-1.65982	-2.05026
H	-7.78112	-1.33992	-0.43332
O	-6.69572	-0.62583	2.40492
H	-5.89955	-1.28357	2.30299
H	-6.37643	0.27083	2.12687
O	-4.70943	-2.23599	2.06595
H	-3.83727	-1.84166	2.32264
H	-4.62985	-2.53607	1.12016
O	-9.03486	0.83283	-0.73073
H	-8.4381	0.2015	-1.2972
H	-8.38917	1.52306	-0.41957
C	2.93846	-3.53536	-0.25208
C	1.72242	-3.78625	0.40676
C	4.07623	-4.27906	0.10316
C	1.65584	-4.75644	1.40703
H	0.84636	-3.21365	0.15208
C	3.99335	-5.24841	1.10283
H	5.00522	-4.11738	-0.41731
C	2.78786	-5.49248	1.76309
H	0.70976	-4.93605	1.90397
H	4.88099	-5.81299	1.36274
H	2.73047	-6.24493	2.54016

Figure 7.

Isobutyraldehyde(1)

Calculation Type = FREQ

Calculation Method = RB3LYP

Basis Set = 6-31G(d,p)

Charge = 0

Spin = Singlet

Solvation = scrf=solvent=water

E(RB3LYP) = -231.19058 Hartree

RMS Gradient Norm = 1.3092e-05 Hartree/Bohr

Temperature = 298.15 Kelvin

Pressure = 1 atm

Frequencies scaled by = 1

Electronic Energy (EE) = -231.19058 Hartree

Zero-point Energy Correction = 0.113758 Hartree

Thermal Correction to Energy = 0.119987 Hartree

Thermal Correction to Enthalpy = 0.120931 Hartree

Thermal Correction to Free Energy = 0.084489 Hartree

EE + Zero-point Energy = -231.07683 Hartree

EE + Thermal Energy Correction = -231.0706 Hartree

EE + Thermal Enthalpy Correction = -231.06965 Hartree

EE + Thermal Free Energy Correction = -231.10609 Hartree

E (Thermal) = 75.293 kcal/mol

Heat Capacity (Cv) = 21.001 cal/mol-kelvin

Entropy (S) = 76.699 cal/mol-kelvin

Symbolic Z-matrix:

Charge = 0 Multiplicity = 1

O	-1.8992	-0.10315	-0.26595
H	-0.91113	-1.73269	0.47759
C	0.43632	0.01567	0.43676

C	1.54479	-0.81135	-0.26368
H	0.61992	-0.02198	1.52255
H	1.40682	-0.78259	-1.35008
H	2.52952	-0.39606	-0.02739
H	1.5247	-1.85767	0.06339
C	-0.90422	-0.65864	0.19693
C	0.40064	1.47363	-0.04454
H	-0.41764	2.01768	0.43591
H	1.34567	1.97845	0.17845
H	0.23052	1.50422	-1.12573

***N*-Phenylmaleimide(2)**

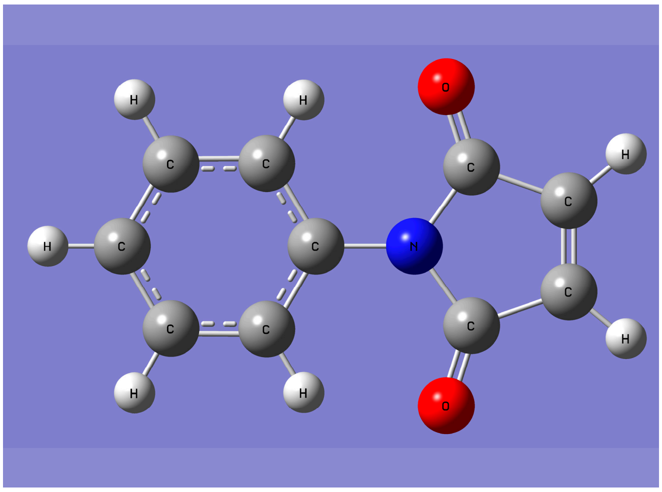

Calculation Type = FREQ

Calculation Method = RB3LYP

Basis Set = 6-31G(d,p)

Charge = 0

Spin = Singlet

Solvation = scrf=solvent=water

E(RB3LYP) = -587.23576 Hartree

RMS Gradient Norm = 6.6e-08 Hartree/Bohr

Temperature = 298.15 Kelvin

Pressure = 1 atm

Frequencies scaled by = 1

Electronic Energy (EE) = -587.23576 Hartree

Zero-point Energy Correction = 0.150857 Hartree

Thermal Correction to Energy = 0.160386 Hartree

Thermal Correction to Enthalpy = 0.16133 Hartree

Thermal Correction to Free Energy = 0.114807 Hartree

EE + Zero-point Energy = -587.08491 Hartree

EE + Thermal Energy Correction = -587.07538 Hartree

EE + Thermal Enthalpy Correction = -587.07443 Hartree

EE + Thermal Free Energy Correction = -587.12095 Hartree

E (Thermal) = 100.644 kcal/mol

Heat Capacity (Cv) = 37.562 cal/mol-kelvin

Entropy (S) = 97.917 cal/mol-kelvin

Symbolic Z-matrix:

Charge = 0 Multiplicity = 1

C	1.59603	-1.15994	-0.01622
C	3.01362	-0.66487	-0.00952
C	3.01305	0.66641	0.01062
C	1.59507	1.16022	0.01577
H	3.84643	-1.34763	-0.0194
H	3.84529	1.34985	0.02159
O	1.2295	-2.33409	-0.02985
O	1.22711	2.33387	0.0299
N	0.7625	-0.00029	-0.00113
C	-0.67254	-0.00045	-0.00018
C	-1.38986	-1.21078	0.03381
C	-1.38954	1.21009	-0.03432
C	-2.78428	-1.20165	0.03416
H	-0.85001	-2.14195	0.05294
C	-2.78395	1.20138	-0.03373
H	-0.84971	2.14123	-0.05437
C	-3.49376	-0.00004	0.00054
H	-3.31439	-2.1468	0.06073
H	-3.3138	2.14668	-0.06036
H	-4.57709	0.00013	0.00086

1b Cat. (3)

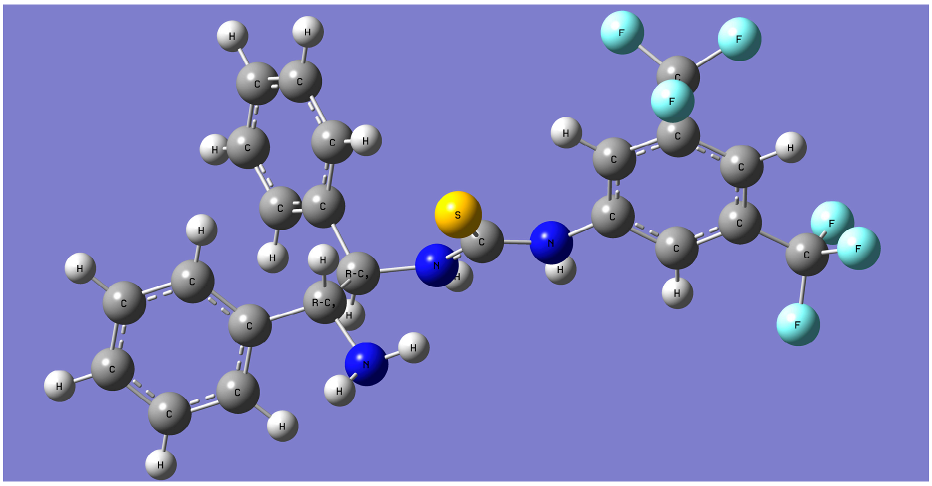

Calculation Type = FREQ

Calculation Method = RB3LYP

Basis Set = 6-31G(d,p)

Charge = 0

Spin = Singlet

Solvation = scrf=solvent=water

E(RB3LYP) = -2038.4563 Hartree

RMS Gradient Norm = 4.83e-07 Hartree/Bohr

Temperature = 298.15 Kelvin

Pressure = 1 atm

Frequencies scaled by = 1

Electronic Energy (EE) = -2038.4563 Hartree

Zero-point Energy Correction = 0.389951 Hartree

Thermal Correction to Energy = 0.419074 Hartree

Thermal Correction to Enthalpy = 0.420018 Hartree

Thermal Correction to Free Energy = 0.324348 Hartree

EE + Zero-point Energy = -2038.0664 Hartree

EE + Thermal Energy Correction = -2038.0373 Hartree

EE + Thermal Enthalpy Correction = -2038.0363 Hartree

EE + Thermal Free Energy Correction = -2038.132 Hartree

E (Thermal) = 262.973 kcal/mol

Heat Capacity (Cv) = 110.61 cal/mol-kelvin

Entropy (S) = 201.355 cal/mol-kelvin

Symbolic Z-matrix:

Charge = 0 Multiplicity = 1

C	5.95702	-2.0257	-0.99747
C	5.31343	-0.78977	-1.15311
C	6.08417	0.35455	-1.3923
C	7.4772	0.26852	-1.46758
C	8.11327	-0.96582	-1.30522
C	7.34877	-2.11364	-1.07154
H	5.34877	-2.90778	-0.84029
H	5.59286	1.31319	-1.51709
H	8.06328	1.16099	-1.65527
H	9.19349	-1.03345	-1.36527
H	7.83615	-3.07477	-0.95197
C	3.89545	0.58264	1.1739
C	4.94237	0.48821	2.10013
C	5.41299	1.6259	2.76155
C	4.83664	2.8724	2.50476
C	3.78736	2.97356	1.5853
C	3.31822	1.83683	0.92364
H	5.39328	-0.47753	2.30146
H	6.22394	1.53746	3.47528
H	5.19895	3.75603	3.01732
H	3.33395	3.93758	1.38433
H	2.50024	1.91008	0.21708
C	3.79434	-0.70344	-1.05643
C	3.40676	-0.67122	0.45678
N	1.9588	-0.92522	0.7652

N	3.20105	-1.93013	-1.64968
H	3.90898	-1.5372	0.89565
H	3.45423	0.22643	-1.5242
H	3.5871	-2.07576	-2.59057
H	1.85504	-1.45948	1.62979
C	0.82062	-0.50323	0.18225
C	-2.09556	0.64191	0.19432
C	-3.45486	0.89091	0.02454
C	-4.42239	-0.08562	0.23553
C	-3.98211	-1.35313	0.6043
C	-2.63084	-1.6402	0.76007
C	-1.66918	-0.63958	0.56089
H	-1.38016	1.43301	0.04259
H	-5.49698	0.11274	0.11464
H	-2.32892	-2.64378	1.02737
N	-0.31601	-0.96901	0.80595
H	-0.18708	-1.68947	1.51789
F	-5.87206	-2.51813	-0.24276
F	-5.77538	-2.18874	1.9346
F	-4.40485	-3.65399	0.98784
F	-2.95742	3.20037	-0.2284
F	-4.0937	2.21453	-1.85513
F	-5.0932	2.6085	0.07102
C	-4.99283	-2.42396	0.82923
C	-3.88315	2.22416	-0.46421
S	0.74853	0.4812	-1.26775
H	2.18444	-1.80902	-1.73222

H_2O

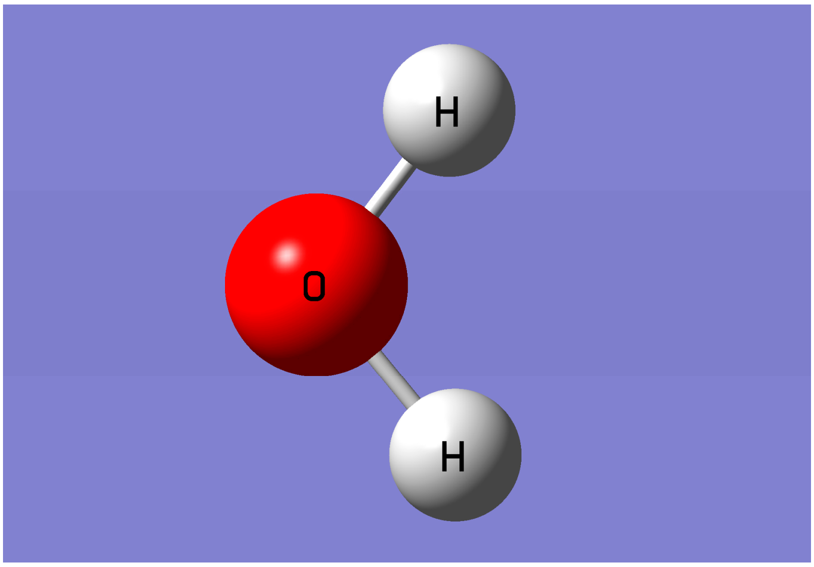

Calculation Type = FREQ

Calculation Method = RB3LYP

Basis Set = 6-31G(d,p)

Charge = 0

Spin = Singlet

Solvation = scrf=solvent=water

E(RB3LYP) = -75.981704 Hartree

RMS Gradient Norm = 0.000103805 Hartree/Bohr

Temperature = 298.15 Kelvin

Pressure = 1 atm

Frequencies scaled by = 1

Electronic Energy (EE) = -75.981704 Hartree

Zero-point Energy Correction = 0.019862 Hartree

Thermal Correction to Energy = 0.022697 Hartree

Thermal Correction to Enthalpy = 0.023641 Hartree

Thermal Correction to Free Energy = 0.002118 Hartree

EE + Zero-point Energy = -75.961842 Hartree

EE + Thermal Energy Correction = -75.959007 Hartree

EE + Thermal Enthalpy Correction = -75.958063 Hartree

EE + Thermal Free Energy Correction = -75.979586 Hartree

E (Thermal) = 14.243 kcal/mol

Heat Capacity (Cv) = 5.998 cal/mol-kelvin

Entropy (S) = 45.3 cal/mol-kelvin

Symbolic Z-matrix:

Charge = 0 Multiplicity = 1

O	-2.30435	1.26087	0.
H	-1.34435	1.26087	0.
H	-2.6248	2.16581	0.

TS

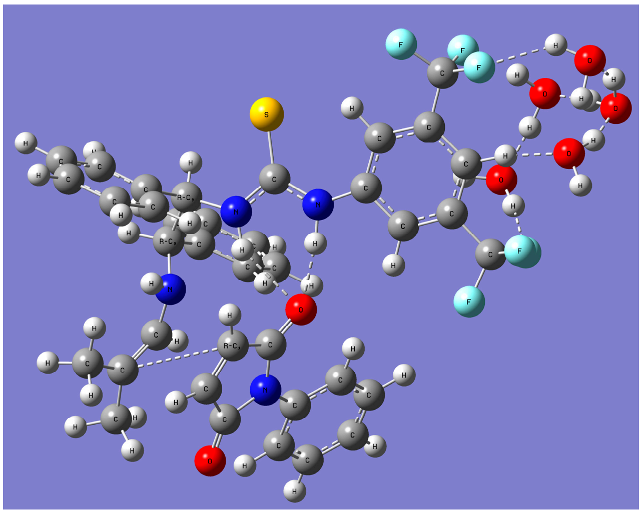

Calculation Type = FREQ

Calculation Method = RB3LYP

Basis Set = 6-31G(d,p)

Charge = 0

Spin = Singlet

Solvation = scrf=solvent=water

E(RB3LYP) = -3160.9918 Hartree

RMS Gradient Norm = 8.18e-07 Hartree/Bohr

Temperature = 298.15 Kelvin

Pressure = 1 atm

Frequencies scaled by = 1

Electronic Energy (EE) = -3160.9918 Hartree

Zero-point Energy Correction = 0.757812 Hartree

Thermal Correction to Energy = 0.813916 Hartree

Thermal Correction to Enthalpy = 0.81486 Hartree

Thermal Correction to Free Energy = 0.659441 Hartree

EE + Zero-point Energy = -3160.234 Hartree

EE + Thermal Energy Correction = -3160.1779 Hartree

EE + Thermal Enthalpy Correction = -3160.177 Hartree

EE + Thermal Free Energy Correction = -3160.3324 Hartree

E (Thermal) = 510.74 kcal/mol

Heat Capacity (Cv) = 209.564 cal/mol-kelvin

Entropy (S) = 327.108 cal/mol-kelvin

Symbolic Z-matrix:

Charge = 0 Multiplicity = 1

C	2.67135	-0.37866	2.27695
C	3.46932	0.7741	2.32576
C	4.01466	1.16863	3.55649
C	3.76706	0.43327	4.71773
C	2.97651	-0.71749	4.66011
C	2.43376	-1.12065	3.43705
H	2.23258	-0.70878	1.34584
H	4.64036	2.05392	3.60405
H	4.19706	0.75284	5.65985
H	2.78972	-1.29634	5.5571
H	1.82883	-2.01824	3.37907
C	2.97449	3.69365	-0.1753
C	3.74277	4.75668	0.32284
C	4.22791	5.74413	-0.53557
C	3.95166	5.68035	-1.9057
C	3.18164	4.62889	-2.40687
C	2.69178	3.64124	-1.54538
H	3.95657	4.81431	1.38547
H	4.81625	6.5626	-0.13738
H	4.32856	6.44641	-2.573
H	2.95575	4.57715	-3.46564
H	2.07471	2.84179	-1.93635
C	3.73623	1.64744	1.10737
C	2.52633	2.59581	0.79248

N	1.4668	1.74626	0.24901
N	4.00052	0.84791	-0.13506
H	2.175	3.04869	1.72236
H	4.59319	2.29492	1.33384
H	1.82661	0.89845	-0.19711
C	0.1343	1.94905	0.36347
C	-2.98852	1.60667	0.00795
C	-4.32	1.25505	-0.21104
C	-4.69608	0.02995	-0.75013
C	-3.66877	-0.86185	-1.06217
C	-2.33406	-0.54837	-0.85315
C	-1.9686	0.70173	-0.31486
H	-2.74146	2.57397	0.414
H	-5.74605	-0.23964	-0.95571
H	-1.5656	-1.26447	-1.11491
N	-0.59247	0.91882	-0.17128
S	-0.50681	3.38302	1.13564
H	-0.01959	0.12482	-0.50294
F	-5.00827	-2.15595	-2.5483
F	-4.52801	-3.04714	-0.5919
F	-2.93743	-2.85411	-2.12265
F	-4.91467	3.50705	0.24341
F	-6.47204	2.16958	-0.63433
F	-5.86997	1.9532	1.48192
C	-4.019	-2.20461	-1.59459
C	-5.37239	2.21924	0.20243
C	2.12938	-1.47314	-1.46164
C	3.68678	-1.28128	-3.14593
C	4.03995	-2.4621	-2.29753
O	1.15575	-1.20941	-0.72425
C	2.57708	-0.71421	-2.66633

O	4.98727	-3.23266	-2.42183
N	3.01586	-2.53797	-1.28167
H	2.04071	0.14572	-3.02994
H	4.07898	1.47879	-0.93913
C	5.05025	-0.10902	-0.07585
C	6.07951	-0.24033	-0.93432
C	6.38763	0.71708	-2.07009
C	7.04461	-1.4	-0.79066
H	4.94762	-0.80391	0.75057
H	7.39575	1.13153	-1.93467
H	6.38028	0.19772	-3.03634
H	5.68601	1.55415	-2.12948
H	6.90336	-2.11293	-1.61476
H	8.08295	-1.04648	-0.8205
H	6.88369	-1.93856	0.14951
H	4.28026	-0.99785	-3.99798
O	-8.51282	-1.27177	0.74586
H	-7.80545	-1.02147	1.49375
H	-8.9445	-0.39523	0.4263
O	-7.35031	-0.95339	-1.38066
H	-7.17521	-1.65982	-2.05026
H	-7.78112	-1.33992	-0.43332
O	-6.69572	-0.62583	2.40492
H	-5.89955	-1.28357	2.30299
H	-6.37643	0.27083	2.12687
O	-4.70943	-2.23599	2.06595
H	-3.83727	-1.84166	2.32264
H	-4.62985	-2.53607	1.12016
O	-9.03486	0.83283	-0.73073
H	-8.4381	0.2015	-1.2972
H	-8.38917	1.52306	-0.41957

C	2.93846	-3.53536	-0.25208
C	1.72242	-3.78625	0.40676
C	4.07623	-4.27906	0.10316
C	1.65584	-4.75644	1.40703
H	0.84636	-3.21365	0.15208
C	3.99335	-5.24841	1.10283
H	5.00522	-4.11738	-0.41731
C	2.78786	-5.49248	1.76309
H	0.70976	-4.93605	1.90397
H	4.88099	-5.81299	1.36274
H	2.73047	-6.24493	2.54016

4. References

1. Miura, T.; Masuda, A.; Ina, M.; Nakashima, K.; Nishida, S.; Tada, N.; Itoh, A. Asymmetric Michael reactions of α, α-disubstituted aldehydes with maleimides using a primary amine thiourea organocatalyst. *Tetrahedron Asymmetry* **2011**, *22*, 1605–1609.

2. Bai, J.F.; Peng, L.; Wang, L.L.; Wang, L.X.; Xu, X.Y. Chiral primary amine thiourea promoted highly enantioselective Michael reactions of isobutylaldehyde with maleimides. *Tetrahedron* **2010**, *66*, 8928–8932.

3. Ma, Z.-w.; Liu, Y.-x.; Li, P.l.; Ren, H.; Zhu, Y.; Tao, J.C. A highly efficient large-scale asymmetric Michael addition of isobu-tyraldehyde to maleimides promoted by a novel multifunctional thiourea. *Tetrahedron: Asymmetry* **2011**, *22*, 1740–1748.

4. Yu, F.; Jin, Z.; Huang, H.; Ye, T.; Liang, X.; Ye, J. A highly efficient asymmetric Michael addition of α, α-disubstituted aldehydes to maleimides catalyzed by primary amine thiourea salt. *Org. Biomol. Chem.* **2010**, *8*, 4767–4774.

5. Xue, F.; Liu, L.; Zhang, S.; Duan, W.; Wang, W. A simple primary amine thiourea catalyzed highly enantioselective conjugate addition of α,α-disubstituted aldehydes to maleimides. *Chem. Eur. J.* **2010**, *16*, 7979–7892.

6. Kokotos, C.G. An Asymmetric Michael Addition of α,α-Disubstituted Aldehydes to Maleimides Leading to a One-Pot Enan-tioselective Synthesis of Lactones Catalyzed by Amino Acids. *Org. Lett.* **2013**, *15*, 2406–2409.

7. Ma, Z.W.; Liu, X.F.; Liu, J.T.; Liu, Z.J.; Tao, J.C. Highly enantioselective Michael addition of α, α-disubstituted aldehydes to maleimides catalyzed by new primary amine-squaramide bifunctional organocatalysts. *Tetrahedron Lett.* **2017**, *58*, 4487–4490.
